# Vascular smooth muscle cell RNA-binding protein U2AF2 induces copper death by regulating C1qbp expression, delaying development of atherosclerosise

**DOI:** 10.1186/s40659-026-00672-3

**Published:** 2026-01-28

**Authors:** Yang Yang, Hongxu Chen, Qijun Yu, Qihui Chen, Xinyue Zhou, Shanshan Zhou, Tingjiao Liu, Jinghan Lin

**Affiliations:** Department of Neurology, The First Affiliated Hospital of Harbin Medical University, No. 23, Postal Street, Nangang District, Harbin, 150001 Heilongjiang China

**Keywords:** Atherosclerosis, Vascular smooth muscle cells, Copper death, U2AF2, C1qbp

## Abstract

**Background:**

Atherosclerosis (AS) is the main pathological basis of atherosclerosis-related cardiovascular and cerebrovascular diseases. The phenotypic conversion and death mechanisms of vascular smooth muscle cells (VSMCs) are crucial during its development. This study reveals the molecular mechanisms of the C1qbp-DLAT axis and the U2AF2 (U2 Small Nuclear RNA Auxiliary Factor 2)—NEAT1 network in regulating cuproptosis in AS.

**Methods:**

In this study, an ApoE^−/−^ mouse model was constructed by high-fat diet (HFD) induction. Cell culture, molecular biology, immunology and histology methods were employed to explore the role of the U2AF2-C1qbp-copper death regulatory axis in the development of AS. Techniques such as qRT-PCR, Western blot, immunoprecipitation, RNA pull-down and RIP were used to detect the expression of related genes and proteins and analyze their functions.

**Results:**

The study revealed elevated copper ion levels and dysregulated cuproptosis-related genes in an AS model. U2AF2 stabilized C1qbp mRNA, enhancing C1qbp protein expression, which promoted DLAT oligomerization to regulate cuproptosis. LncRNA NEAT1 facilitated this process by scaffolding U2AF2-C1qbp mRNA interaction. Targeted inhibition of U2AF2 significantly improved AS pathological characteristics, reduced lipid deposition, collagen deposition and macrophage infiltration within the plaque, increased smooth muscle cell content and lowered serum levels of total cholesterol (TC), total triglyceride (TG) and low-density lipoprotein cholesterol (LDL-C).

**Conclusion:**

This study revealed the role of the U2AF2-C1qbp-copper death regulatory axis in the development of AS, providing new targets and a theoretical basis for the treatment of AS. Targeted inhibition of U2AF2 may become an effective strategy to delay progression of AS.

**Supplementary Information:**

The online version contains supplementary material available at 10.1186/s40659-026-00672-3.

## Introduction

AS, characterized by lipid deposition, chronic inflammation, and vascular wall remodeling, is the underlying pathology of most cardiovascular and cerebrovascular diseases [1]. VSMCs play a dual role in the development of AS: in the early stage, they migrate to the intima through phenotypic transformation (from contractile to synthetic type), engulf lipids to form foam cells and promote the evolution of lipid streaks into fibrous plaques [2]; in the late stage, they maintain the stability of the fibrous cap by secreting extracellular matrix (collagen) to prevent plaque rupture [3]. Studies indicate that 40–90% of plaque cells originate from VSMCs and their phenotypic plasticity (differentiation into macrophage-like cells or myofibroblasts) is a central driver of AS progression [4, 5]. However, the mechanisms regulating VSMC death within the atherosclerotic microenvironment, particularly involving novel forms of programmed cell death, remain incompletely understood.

In recent years, cuproptosis, a novel copper-dependent regulated cell death mode characterized by mitochondrial protein aggregation and metabolic collapse, has gradually attracted attention for its role in AS [6]. Excessive copper ions trigger abnormal mitochondrial lipid acylation, protein aggregation and oxidative stress, leading to cellular energy failure. Copper homeostasis imbalance is closely linked to atherosclerosis-related atherosclerosis related cardiovascular and cerebrovascular diseases, as elevated serum copper levels correlate positively with AS severity and plaque vulnerability, likely through oxidative stress and low-density lipoprotein (LDL) oxidation [7]. Copper chelators can significantly attenuate atherosclerotic lesions, while in the AS microenvironment, copper ions accelerate plaque formation by promoting LDL oxidation, inflammatory cytokine release and endothelial dysfunction [8, 9]. Notably, key cuproptosis-related genes (FDX1 and SLC31A1) are dysregulated in AS plaques [9, 10], suggesting cuproptosis may represent a novel pathogenic mechanism in AS. Nevertheless, whether copper directly triggers VSMC death and how it integrates with other programmed death pathways (e.g., apoptosis, pyroptosis) or modulates VSMC function remain unresolved scientific questions. RNA-binding protein U2AF2 is a core factor in pre-mRNA splicing, ensuring the precise assembly of the spliceosome by recognizing the polypyrimidine region (Py-tract) at the 3' splice site [11]. Its conformational dynamics (switching between open/closed states) determines splicing efficiency and affects the expression of downstream gene isoforms [12]. In the atherosclerosis-related cardiovascular and cerebrovascular diseases system, mutations or abnormal expression of U2AF2 can lead to splicing errors, thereby participating in pathological processes [13]. Notably, U2AF2 can regulate target gene expression by binding to specific RNA sequences [14], but its post-transcriptional regulatory mechanism on VSMCs in AS remains unexplored.

Complement C1q binding protein (C1qbp/p32) is localized in the mitochondrial matrix and is a key molecule for maintaining oxidative phosphorylation (OXPHOS) and cellular metabolic homeostasis [15]. C1qbp regulates the tricarboxylic acid cycle by binding to dihydrolipoamide S-acetyltransferase (DLAT), affecting the activity of mitochondrial respiratory chain complexes. Under pathological conditions, upregulation of C1qbp promotes cell survival, migration and invasion (e.g., in hepatocellular carcinoma), while knockdown of C1qbp induces apoptosis and inhibits energy metabolism [16]. In cardiac tissue, the absence of C1qbp leads to contractile dysfunction in cardiomyocytes and exacerbates oxidative stress, indicating its role in regulating cell fate by maintaining mitochondrial integrity [17]. However, whether C1qbp participates in the copper death pathway, especially its association with VSMC death remains unknown.

Research on the copper death signaling pathway in atherosclerosis still has some gaps. Although studies have shown that copper death is closely related to the occurrence and development of atherosclerosis and its role in cell death, inflammatory response and oxidative stress has been revealed, the specific mechanisms still need to be further explored [18]. This study is the first to identify the “U2AF2-C1qbp-cuproptosis” regulatory axis in AS. Under atherosclerotic conditions, risk factors upregulate U2AF2 in VSMCs, which binds to specific sequences of C1qbp mRNA to modulate its splicing and expression. The subsequent increase in C1qbp protein disrupts mitochondrial copper homeostasis, induces lipoylated protein aggregation, energy crisis and ultimately triggers VSMC cuproptosis, exacerbating plaque instability. Elucidating this mechanism provides novel therapeutic targets for AS and advances precision medicine strategies targeting cuproptosis [19].

## Materials and methods

### Animals

Eight-week-old male C57BL/6J and ApoE^−/−^ mice (purchased from Beijing SF Biotechnology Co., Ltd.) were housed in an SPF environment with controlled temperature (21–26 °C), humidity (40–70%), and a 12-h light/dark cycle. All mice had free access to food and water, and the experiment was started after 1 week of acclimatization. All experimental protocols have been reviewed and approved by the Animal Ethics Committee of The First Affiliated Hospital of Harbin Medical University (YS289).

### Establishment of the mouse model of AS

The AS mouse model was induced using a HFD. After 12 weeks of HFD feeding, sh-U2AF2 lentiviral particles were injected into the tail vein for experimental treatment. At the end of the experiment, all mice were sacrificed. Blood was collected from the abdominal aorta and centrifuged to separate plasma for the measurement of TC, TG, LDL-C and high-density lipoprotein cholesterol (HDL-C). The aorta was collected for subsequent experiments.

C57BL/6J mice were used as the normal control group and the mice were randomly divided into four groups (*n* = 6): (1) Control group: fed a normal diet. (2) AS group: ApoE^−/−^ male mice were fed an HFD. (3) AS + LV-sh-NC group: ApoE^−/−^ male mice were injected with sh-NC lentiviral particles via the tail vein and after 4 weeks, they were fed an HFD for 12 weeks. (4) AS + LV-sh-U2AF2 group: ApoE^−/−^ male mice were injected with sh-U2AF2 lentiviral particles via the tail vein and after 4 weeks, they were fed an HFD for 12 weeks.

### Cell culture and treatment

Human aortic vascular smooth muscle cells (HAVSMCs) were purchased from the Shanghai Cell Bank. The cells were cultured in Dulbecco's Modified Eagle Medium (DMEM) (G4520-500ML, Servier) containing 10% fetal bovine serum (C04001-500, Xiaopeng Biological) and 1% penicillin–streptomycin (G4003, Servier).

HAVSMC cells were treated with the copper ion carrier Elesclomol-Cu^2+^ (ES-Cu^2+^) and placed in an incubator until the cells grew to approximately 70–80%. In the transfection experiments, Lipofectamine 8000 (4 μL) was used to transfect cells with empty vectors and plasmid vectors encoding si-C1qbp (2.5 μL), si-U2AF2 (2.5 μL), si-lncRNA NEAT1 (2.5 μL), si-NC/lncRNA (2.5 μL), OE-U2AF2 (2.5 μL) and OE-NC (2.5 μL). After 24–48 h of transfection, the cells were collected for subsequent experiments.

### Histological staining

Mouse aortic tissue was fixed with 4% paraformaldehyde, embedded in paraffin and sectioned at 4 µm. The following histological staining methods were used to evaluate the pathological changes in the aortic tissue:

HE Staining: Tissue sections were deparaffinized and hydrated. They were stained with hematoxylin (G1004, Servier) for 5 min and then with eosin (G1001, Servier) for 2 min. The slides were then dehydrated, cleared and coverslipped. Microscopic examination was used to evaluate the structure and inflammatory response of mouse aortic tissue.

Masson's Trichrome Staining: Using a Masson's trichrome staining kit (G1340, Solarbio), sections were deparaffinized and hydrated. They were stained with Weigert's iron hematoxylin for 5–10 min, differentiated in an acidic differentiation solution for 10 s and then stained with ponceau red for 8 min. Following this, they were treated with phosphomolybdic acid solution and finally stained with aniline blue staining solution for 1–2 min. Microscopy was used to observe collagen content in the aortic tissue after treatment.

Immunohistochemistry: Immunohistochemistry was performed using a universal kit (PV-6000, Zhongshan Jinqiao). After deparaffinization and hydration, aortic tissue sections were treated with an antigen retrieval solution and endogenous peroxidase inhibitor. Then, primary antibodies against α-SMA (1:200, BM4172, BOSTER), CD68 (1:200, BA3638, BOSTER), C1QBP (1:200, PB0991, BOSTER) and U2AF2 (1:200, M03639-2, BOSTER) were incubated overnight at 4 °C. After incubation, sections were treated with HRP-conjugated goat anti-mouse IgG secondary polymer (100 µL) at 37 °C for 20 min. DAB chromogen was used for visualization, followed by counterstaining with hematoxylin. Aortic tissues were observed and analyzed under a light microscope.

### Immunofluorescence staining

After dewaxing and rehydration, sections were placed in a microwave oven at P100 power. Following boiling of the antigen retrieval solution, sections were heated for 10 min to restore antigens. Add 100 μL of 0.2% Triton X-100 (P0096-100ML, Beyotime) and incubate at room temperature for 15 min to permeabilize antigens. Block with 3% BSA for 30 min. incubate overnight with CD68 (E3O7V) Mouse Chimeric Monoclonal Antibody (1:200, 41077 T, CST). Incubate for 1 h in the dark with Cy3-labeled goat anti-mouse IgG (H + L) (A0521, Beyotime). Incubate again with anti-a-SMA/ACTA2 antibody (1:200, BM3902, Boster) and FITC-labeled goat anti-rabbit IgG (H + L) (A0562, Beyotime). Add DAPI (G1012-100L, Servicebio) and incubate in the dark for 15 min. Mount the slides with an anti-fade mounting medium (G1401-5ML, Servicebio). Finally, observe under a fluorescence microscope (BZ-X800, Keyence Corporation).

### Terminal deoxynucleotidyl transferase dUTP nick end labeling (TUNEL) staining

Aortic tissues were processed according to the TUNEL Apoptosis Assay Kit (G1507-50 T, Servier), followed by fixation with 4% paraformaldehyde for 30 min (G1101, Servier). PBS containing 0.3% Triton X-100 was added and incubated at room temperature for 5 min. Then, 100 μL of TUNEL detection solution was added to the samples and incubated at 37 °C in the dark for 60 min. Meanwhile, DAPI solution was used to stain the cell nuclei. Finally, images were collected using a fluorescence microscope.

### Oil red O staining

Mouse aortic tissues from each group were collected and prepared into cry sections with a thickness of 8–10 μm (CRYOSTAR NX50, Thermo). The sections were thoroughly washed with distilled water to remove the fixative color. A small amount of Oil Red O staining solution (G1015, Servier) was added to the tissue sections and stained for 5–10 min. Excess staining solution was washed off with 60% isopropanol (80109218, National Medicine), followed by washing with distilled water. The cell nuclei were counterstained with hematoxylin (G1101, Servier), differentiated in hydrochloric acid alcohol for 1–5 s and then rinsed in running water to return to blue (G1040, Servier). The sections were then cover-slipped with glycerol and photographed for observation. Five random locations per sample were selected for photography to evaluate lipid droplet accumulation in AS plaques.

### JC-1 staining analysis of mitochondrial membrane potential

Mitochondrial membrane potential was detected using the JC-1 fluorescent probe method (C2006, Beyotime). After different treatments, the culture medium was removed and the cells were gently washed once with PBS (C0221A, Beyotime), followed by the addition of 1 mL of fresh culture medium. Subsequently, an equal volume of pre-prepared JC-1 staining working solution was added and the cells were incubated at 37 °C in the dark for 20 min. After incubation, the supernatant was discarded and the cells were washed twice with precooled 1 × JC-1 staining buffer. Immediately, images were observed and collected under a fluorescence microscope (BZ-X800, Keyence) and the changes in mitochondrial membrane potential were evaluated by the ratio of fluorescence intensities.

### Fluorescence intensity detection of reactive oxygen species (ROS) by DCFH-DA probe

The level of intracellular ROS was detected using the DCFH-DA fluorescent probe method (S0033S, Beyotime). After different treatments, the culture medium was removed and cells were incubated with 10 μM DCFH-DA for 20 min at 37 °C in the dark. Subsequently, the cells were washed three times with serum-free culture medium to remove any residual probe. In the positive control group, a significant increase in ROS levels was observed 20–30 min after stimulation. Finally, fluorescence signals were observed and collected under a fluorescence microscope (BZ-X800, Keyence) and the intracellular ROS levels were quantitatively analyzed by measuring fluorescence intensity.

### Staining with Hoechst and PI solutions

Apoptosis detection was performed using the Hoechst 33342/PI double staining method (Hoechst 33342 staining solution, C1025, Beyotime; PI staining solution, C1352S, Beyotime). After different treatments, cells were digested and collected with 0.25% trypsin (without EDTA), adjusted to a density of 2 × 10^5^ cells/mL and seeded in a 48-well plate for overnight culture. After washing three times with PBS, PI staining solution was added first and incubated at 37 °C in the dark for 20 min. After washing, Hoechst 33342 staining solution was added and stained at room temperature in the dark for 10 min. Following three washes with PBS, images were observed and collected under a fluorescence microscope (BZ-X800, Keyence). Hoechst 33342 labeled all nuclei (blue fluorescence), while PI labeled necrotic nuclei (red fluorescence). Apoptosis was analyzed based on the dual staining results.

### Determination of the activity of TCA cycle rate-limiting enzymes α-ketoglutarate dehydrogenase and citrate synthase

The activities of key enzymes in the tricarboxylic acid cycle (TCA) were detected using microassay kits (8482, CheKine). The activity of citrate synthase (CS) was measured using the KTB1023 kit and the activity of α-ketoglutarate dehydrogenase (α-KGDH) was measured using the KTB1240 kit. A microplate reader (Thermo Fisher Scientific Multiskan FC) was preheated for 30 min and the detection wavelength was set to 412 nm. 10 μL of cell sample was mixed with 220 μL of prewarmed (37 °C) Working Reagent and 10 μL of Working Reagent Ⅵ was immediately added and thoroughly mixed. Absorbance values were measured at A1 and A2, respectively and the change in absorbance (ΔA = A2-A1) was calculated. Enzyme activities were determined according to the instructions provided in the kit. All operations were performed on ice to ensure the stability of enzyme activities.

### RNA immunoprecipitation (RIP) assay

RIP was performed to detect the binding of C1qbp mRNA to U2AF2 protein and lncRNA NEAT1 to U2AF2 using the GenSeq RIP Kit (GS-ET-006, Yunxi Bio). After treatment with Cu^2+^ and cholesterol, cells were lysed with Complete Lysis Buffer. The lysate was incubated overnight at 4 °C with magnetic beads coupled to U2AF2 antibody (IPD-ANP10294, APT Bio). Following six washes with RIP Wash Buffer, the complexes were dissociated with Proteinase K buffer and RNA was extracted using phenol–chloroform. Reverse transcription was carried out with the PrimeScript RT reagent Kit (RR037Q, Takara) and qRT-PCR was performed using TB Green Premix Ex Taq II (RR820Q, Takara). Data were analyzed using the 2^−ΔΔCT^ method.

### C1qbp mRNA stability assay using actinomycin D

The transcription inhibitor actinomycin D (S8964, Selleck) was used to block new RNA synthesis and detect the stability of C1qbp mRNA. After treatment with Cu^2+^ and cholesterol, cells were treated with 2–3 μg/mL actinomycin D and collected at 0, 4, 8 and 12 h. Total RNA was extracted using the TRIzol method (15,596,018, Invitrogen). Reverse transcription was performed with the Prime Script RT reagent Kit (RR037Q, Takara) and qRT-PCR was conducted using TB Green Premix Ex Taq II (RR820Q, Takara). β-tubulin was used as an internal reference and the relative expression level of C1qbp mRNA was calculated using the 2^−ΔΔCT^ method. The stability of C1qbp mRNA was analyzed by examining the degradation curve of mRNA over different time points.

### Quantitative real-time fluorescence PCR (qRT-PCR)

Total RNA was extracted from mouse aortic tissue samples and HAVSMC cells (G3013, Servicebio) using TRIzol reagent. The extracted RNA was used as a template for PCR amplification following the instructions of the TaqMan one-step Prime Script RT reagent Kit (RR037Q, Takara). Table 1 provides detailed information on all primers used in this experiment. To accurately evaluate the expression levels of DLAT, SLC31A1, C1qbp, U2AF2 mRNA and lncRNA NEAT1 in each group, GAPDH was used as an internal reference for all experimental data. Each reaction was repeated three times and quantitative analysis was performed using the 2^−∆∆CT^ method.

**Table 1:** Primers used in qRT-PCR

Gene	Forward sequence (5′–3′)	Reverse sequence (5′–3′)
DLAT Human	GAGATGTCCCTCTAGGAACCC	ACAAACACCCTTCCCTTTGGT
SLC31A1 Human	GGGGATGAGCTATATGGACTCC	TCACCAAACCGGAAAACAGTAG
C1qbp Human	AGAAGCGAAATTAGTGCGGAA	CCACGAAATTGGGAGTTGATGTC
U2AF2 Human	ATGACCCCTGACGGTCTGG	GAGCGGAACTCCAAAAAGGC
LncRNA NEAT1 Human	TAAGAGGACCCTGAGGTGGG	CCCACCACCCACACTGTATC
β-tubulin Human	TCCATGAAGGAGGTCGATGA	CAGACGGCTGTCTTGACATT
LncRNA NEAT1 Mouse	ATTGTAGGAGCCAACCTGCC	TACCAGACCGCTGACACAAC
U2AF2 Mouse	ACAGGAAGCGTAGTCACAGTC	TTCGTCGTCTCCTATCCCGAG
C1qbp Mouse β-tubulin Mouse	ATCAAGGAAGTTAGCTTTCAGGC CACGCAGCAGATGTTCGATG	CCATTAGGTGGTCATACAAGGC GTGGACTCACATGGAGTGGG

### Western blotting (WB)

Mouse aortic tissue samples and HAVSMC cells were lysed using RIPA lysis buffer (BL504A, Bio Sharp), followed by protein quantification with the Pierce BCA Protein Assay Kit (BL521A, Bio Sharp). Denature proteins by heating in a 95 °C water bath for 5 min. Specifically, to detect DLAT oligomerization, the protein lysate was not subjected to boiling or ultrasonication, nor were reducing agents added to the buffer to generate non-reducing protein indicators. Proteins were separated by 10% SDS-PAGE (P0015L, Beyotime) and transferred to PVDF membranes via wet transfer. The membranes were blocked with 5% skim milk at room temperature for 2 h. Antibodies were diluted to the target concentration in blocking solution and incubated with the membrane overnight at 4 °C. The primary antibodies used included: DLAT (1:1,000, 12362 T, Cell Signaling), DLST (1:1000, ab72790, Abcam), anti-lipoic acid antibody (1:1000, ab58724, Abcam), SLC31A1 (1:1000, M03447, BOSTER), ATP7A (1:1000, ab308524, Abcam), LIAS (1:1,000, ab96302, Abcam), FDX1 (1:1000, 12592-1-AP, Proteintech), C1qbp (1:1,000, 68084-1-Ig, Proteintech), acylated DLAT (1:1000, 12362 T, Cell Signaling), oligomerized DLAT (1:1000, 12362 T, Cell Signaling), U2AF2 (1:1,000, 68166-1-Ig, Proteintech) and β-tubulin (1:1,000, 10094-1-AP, Proteintech). Subsequently, HRP-conjugated secondary antibodies mouse IgG (1:5000, ab6789, Abcam) and rabbit IgG (1:5000, ab6721, Abcam) were incubated at 37 °C for 2 h. Finally, imaging was performed using an integrated chemiluminescence imaging system (ChampChemi 910, SINSAGE).

### Co-immunoprecipitation (Co-IP)

Cell pellets from each group of HAVSMC cells were collected and treated with RIPA lysis buffer (BL504A, Biosharp). Subsequently, the immunoprecipitation kit was used following the instructions (P2179S, Beyotime). C1qbp primary antibody (1:1000, 68084-1-Ig, Proteintech) and DLAT primary antibody (1:1000, 12362 T, Cell Signaling) were incubated with Protein A + G magnetic beads for 2 h. The cell lysate was then incubated overnight at 4 °C with the primary antibody-bead complex on a shaker. After centrifugation, the protein-bead complex was resuspended in protein lysis buffer. Finally, the immunoprecipitated proteins were analyzed by Western blotting.

### Statistical analysis

All experimental data are presented as mean ± standard deviation (SD) from three independent replicates. Statistical analyses were performed using SPSS 16.0 software (IBM, USA), with intergroup comparisons conducted by one-way ANOVA. Graphical representations were generated using GraphPad Prism 8.0 (GraphPad Software, USA). A *p* < 0.05 was considered statistically significant.

## Results

### Elevated copper death in the AS model and ES-Cu^2+^ treated HAVSMC cell

**Fig. 1:**
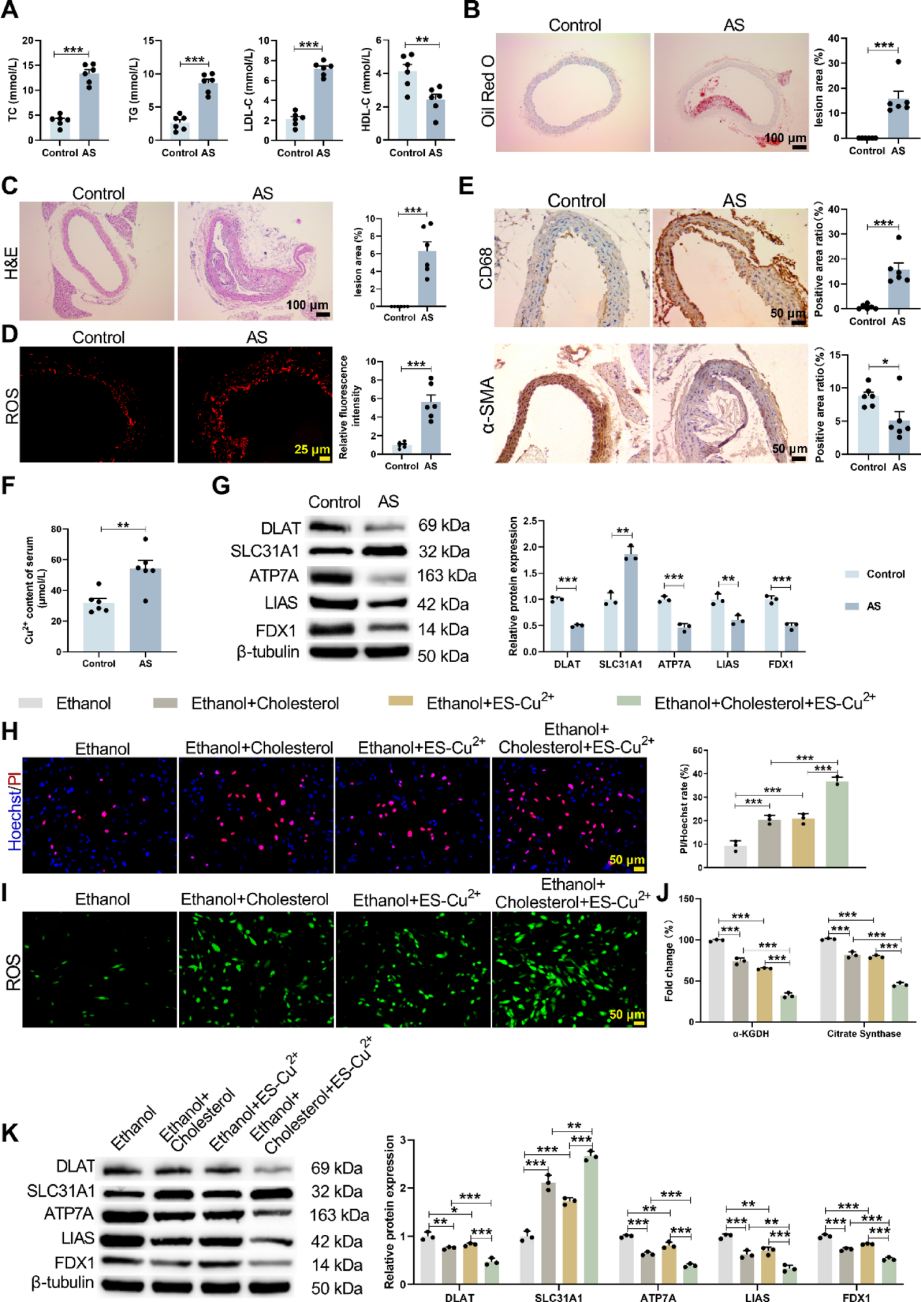
Elevated copper-induced cell death in the AS model. ApoE^−/−^ mice were fed an HFD for 12 weeks to establish the AS model and their aortas were used for experiments. **A** A commercial kit was used to measure plasma levels of TC, TG, LDL-C and HDL-C. HDL-C: Mean_Control_ = 4.128; Mean_AS_ = 2.427; 95% CI [− 2.855, − 0.5484]. LDL-C: Mean_Control_ = 2.115; Mean_AS_ = 7.168; 95% CI [4.171, 5.936]. TC: Mean_Control_ = 3.910; Mean_AS_ = 13.32; 95% CI [7.415, 11.41]. TG: Mean_Control_ = 2.608; Mean_AS_ = 8.608; 95% CI [4.362, 7.638]. **B** Lipid accumulation in aortic plaques was analyzed by Oil Red O staining. Mean_Control_ = 0.000; Mean_AS_ = 15.81; 95% CI [9.120, 22.51]. **C** Plaque morphology, size and necrotic core area in the aortas were assessed by HE staining. Mean_Control_ = 0.000; Mean_AS_ = 6.285; 95% CI [3.876, 8.694]. **D** ROS levels were detected using the fluorescent probe DCFH-DA. Mean_Control_ = 9.453; Mean_AS_ = 22.27; 95% CI [5.498, 20.13]. **E** Immunohistochemistry was performed to evaluate the expression of α-SMA and CD68 in mouse aortas (200X). α-SMA: Mean_Control_ = 8.767; Mean_AS_ = 5.098; 95% CI [− 6.919, − 0.4181]. CD68: Mean_Control_ = 0.7033; Mean_AS_ = 15.76; 95% CI [9.006, 21.10]. **F** Copper ion content was measured using a dedicated assay kit. Mean_Control_ = 32.10; Mean_AS_ = 54.26; 95% CI [8.858, 35.47]. **G** WB was used to analyze the expression of copper-induced cell death-related genes (DLAT, SLC31A1, ATP7A, LIAS and FDX1) in mouse aortas. HAVSMCs were treated with the copper ionophore ES-Cu^2+^. DLAT: Mean_Control_ = 1; Mean_AS_ = 0.5003; 95% CI [− 0.5785, − 0.4208]. SLC31A1: Mean_Control_ = 1; Mean_AS_ = 1.864; 95% CI [0.5517, 1.177]. ATP7A: Mean_Control_ = 1; Mean_AS_ = 0.4682; 95% CI [− 0.6814, − 0.3821]. LIAS: Mean_Control_ = 1; Mean_AS_ = 0.6063; 95% CI [− 0.6198, − 0.1676]. FDX1: Mean_Control_ = 1; Mean_AS_ = 0.4903; 95% CI [− 0.6642, − 0.3553]. **H** Hoechst/PI staining was used to quantify the red/blue fluorescence intensity ratio (indicating cell death) in HAVSMCs. **I** ROS levels were detected using the DCFH-DA probe. **J** The activity of TCA cycle rate-limiting enzymes (α-ketoglutarate dehydrogenase and citrate synthase) in HAVSMCs was measured using assay kits. **K** WB was performed to assess the expression of copper detoxification-related genes (DLAT, SLC31A1, ATP7A, LIAS and FDX1) in HAVSMCs. *p < 0.05; **p < 0.01; ***p < 0.001 versus Control/Ethanol/Ethanol + Cholesterol/Ethanol + ES-Cu^2+^

To investigate the role of copper death in AS, we established an AS model by feeding ApoE^−/−^ mice an HFD for 12 weeks. Compared with the Control group, the AS group exhibited significantly elevated levels of TC, TG and LDL-C in plasma, along with reduced HDL-C levels (Fig. 1A). Oil Red O staining, HE staining and Masson’s trichrome staining further revealed that, compared with the Control group, the AS group displayed increased lipid deposition in aortic plaques (Fig. 1B), exhibited thickened vascular walls with prominent atherosclerotic plaques and foam cells (Fig. 1C), and markedly enhanced collagen deposition (Supplementary Fig. 1A). ROS and TUNEL assays demonstrated significantly higher ROS levels and apoptotic cell counts in the AS group versus controls (Fig. 1D, Supplementary Fig. 1B). Immunohistochemistry analysis revealed increased expression of CD68 and reduced expression of α-SMA in the AS group (Fig. 1E), furthermore, colocalization of CD68 and α-SMA was observed (Supplementary Fig. 1C). Atomic absorption spectrometry showed elevated copper ion content (Fig. 1F). Western blot analysis indicated significant upregulation of the copper death-related gene SLC31A1 in the AS group. Conversely, expression levels of DLAT, ATP7A, LIAS and FDX1 were markedly downregulated compared to controls (Fig. 1G).

To further explore the underlying mechanisms, we treated HAVSMCs with the copper ionophore ES-Cu^2+^ in combination with cholesterol. CCK8 assays confirmed that ethanol treatment had no significant effect on cell proliferation, whereas treatment with Ethanol + Cholesterol reduced cell proliferative capacity. Compared to the Ethanol + ES-Cu^2+^ group, the Ethanol + Cholesterol + ES-Cu^2+^ group exhibited a further reduction in cell proliferation (Supplementary Fig. 1D). TUNEL assays further validated that combined treatment with cholesterol and ES-Cu^2+^ significantly promoted apoptosis (Supplementary Fig. 1E, F). Hoechst/PI staining and ROS detection revealed that, compared to the Ethanol group, treatment with cholesterol or ES-Cu^2+^ significantly increased cell mortality and ROS levels. Moreover, combined treatment with Cholesterol + ES-Cu^2+^ further increased cell mortality and ROS levels (Fig. 1H, I). Additionally, TCA cycle enzyme activity and JC-1 staining assays showed that, compared to the Ethanol group, treatment with Cholesterol or ES-Cu^2+^ significantly reduced α-KGDH, Citrate synthase activity and mitochondrial membrane potential. However, combined treatment with Cholesterol + ES-Cu^2+^ further decreased these parameters (Fig. 1J, Supplementary Fig. 1G, H). Western blot and qRT-PCR results further confirmed that, after treatment with cholesterol or ES-Cu^2+^, SLC31A1 expression was significantly upregulated, while the expression levels of DLAT, ATP7A, LIAS and FDX1 were significantly downregulated. Furthermore, co-treatment with cholesterol + ES-Cu^2+^ deepened these changes (Fig. 1K, Supplementary Fig. 1I). These results indicate that the copper ionophore ES-Cu^2+^ can induce copper death in HAVSMCs, accompanied by elevated ROS levels and impaired mitochondrial function.

### Elevated C1qbp promotes DLAT oligomerization and regulates copper death in vitro

**Fig. 2:**
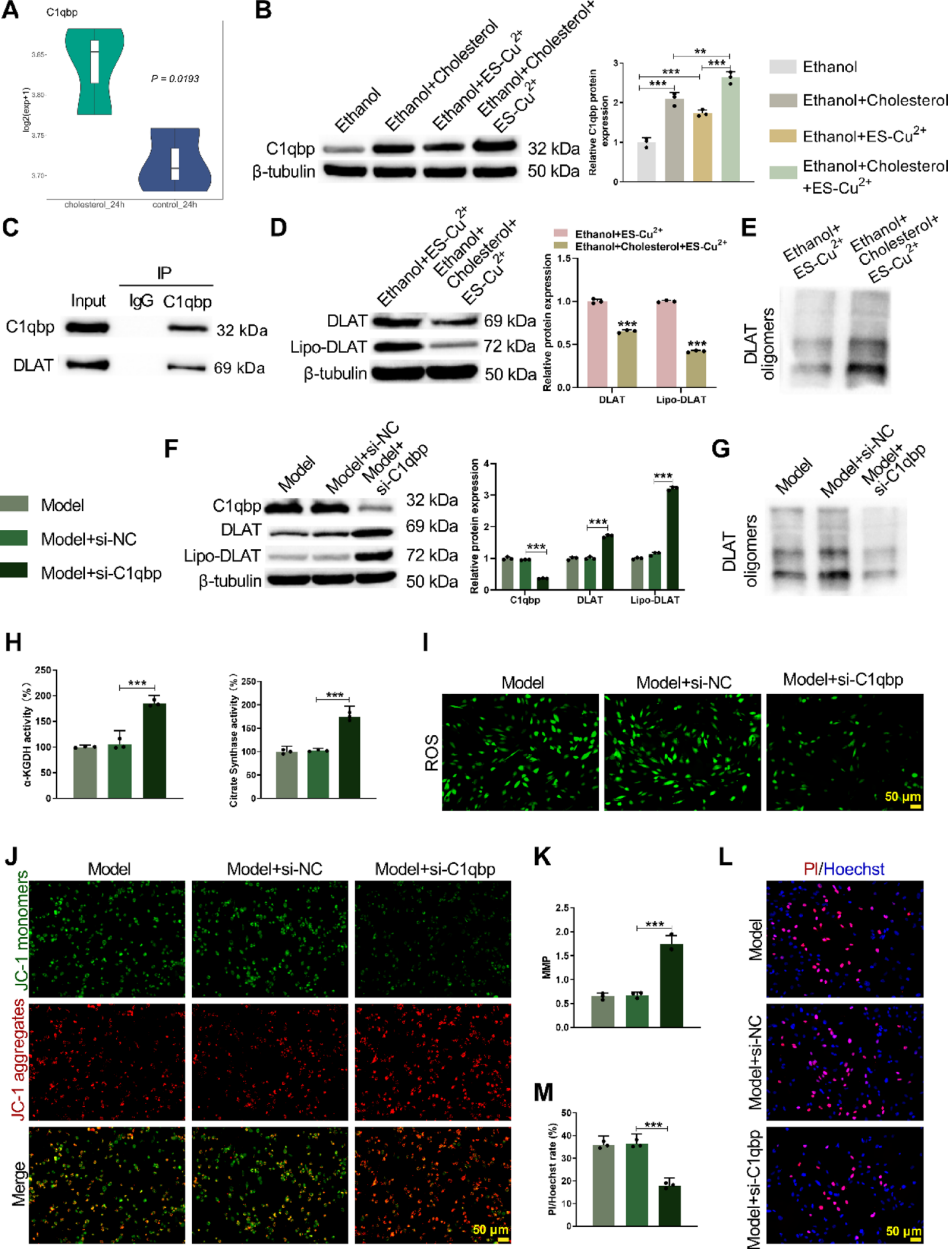
Elevated C1qbp expression promotes DLAT oligomerization and modulates copper-induced cell death. **A** Analysis of the dataset GSE174029 revealed that smooth muscle cells stimulated with cholesterol for 24 h exhibited higher C1qbp mRNA levels compared to control cells. The copper ion carrier ES-Cu^2+^ was used to treat HAVSMC cells. **B** WB detected the protein expression level of C1qbp in HAVSMC cells. **C** Co-IP detected the interaction between C1qbp and DLAT in HAVSMC cells. **D**, **E** WB detected the expression levels of DLAT protein, acylated DLAT protein and oligomerized DLAT protein in HAVSMC cells. The HAVSMC cell line was jointly treated with cholesterol and ES-Cu^2+^ and si-C1QBP were carried out. **F**, **G** WB detected the expression levels of C1qbp protein, DLAT protein, acylated DLAT protein and oligomerized DLAT protein. (H) A commercial kit was used to measure the activity of TCA cycle rate-limiting enzymes, α-ketoglutarate dehydrogenase and citrate synthase. (I) ROS levels were detected using the fluorescent probe DCFH-DA. **J**, **K** Mitochondrial membrane potential was evaluated via JC-1 staining. **L**, **M** Hoechst/PI staining quantified the ratio of red to blue fluorescence intensity in HAVSMC cells. *p < 0.05; **p < 0.01; ***p < 0.001 versus Ethanol/Ethanol + Cholesterol/Ethanol + ES-Cu^2+^/Model + si-NC. NC means negative control of si-C1qbp

Analysis of the GSE174029 dataset revealed a significant upregulation of C1qbp mRNA expression in HAVSMCs after 24 h of cholesterol stimulation (Fig. 2A). Subsequently, HAVSMCs were treated with cholesterol or/and copper ionophore ES-Cu^2+^. Western blot and qRT-PCR analysis confirmed that, compared to the Ethanol group, C1qbp expression was significantly increased after treatment with cholesterol or ES-Cu^2+^. Furthermore, the Ethanol + Cholesterol + ES-Cu^2+^ group exhibited even higher C1qbp expression (Fig. 2B, Supplementary Fig. 2A). Co-IP experiments demonstrated a direct interaction between C1qbp and DLAT proteins (Fig. 2C). Notably, Western blot analysis further showed that, combined treatment with cholesterol + ES-Cu^2+^ significantly reduced DLAT protein expression and its lipoylated form (Lipo-DLAT), while promoting DLAT oligomer formation (Fig. 2D, E). The lipidation and oligomerization of mitochondrial proteins DLAT and dihydrolipoamide S-succinyltransferase (DLST) affect mitochondrial function [20]. Therefore, we also detected the expression of the DLST protein and its lipidated form (Lipo-DLST), observing the same trend with DLAT protein expression and Lipo-DLAT levels (Supplementary Fig. 2B). After transfection with si-C1qbp, knockdown efficiency was validated (Supplementary Fig. 6A, B). Western blot and qRT-PCR analysis revealed that, after treatment with si-C1qbp, DLAT protein expression and Lipo-DLAT levels were significantly increased, while C1qbp expression was markedly reduced (Fig. 2F, Supplementary Fig. 2C), accompanied by reduced DLAT oligomer formation (Fig. 2G). DLST protein expression and Lipo-DLST levels show the same trend with DLAT protein expression and Lipo-DLAT levels (Supplementary Fig. 2D). Functional rescue experiments demonstrated that, si-C1qbp reversed copper death phenotypes, including restoring the activity of TCA cycle rate-limiting enzymes (α-ketoglutarate dehydrogenase and citrate synthase) (Fig. 2H), reducing intracellular ROS levels (Fig. 2I), improving mitochondrial membrane potential (Fig. 2J, K), and decreasing cell death (Fig. 2L, M). These results indicate that C1qbp plays a critical role in regulating copper death by promoting DLAT oligomerization in AS.

### U2AF2 regulates C1qbp mRNA stability to modulate copper death

**Fig. 3:**
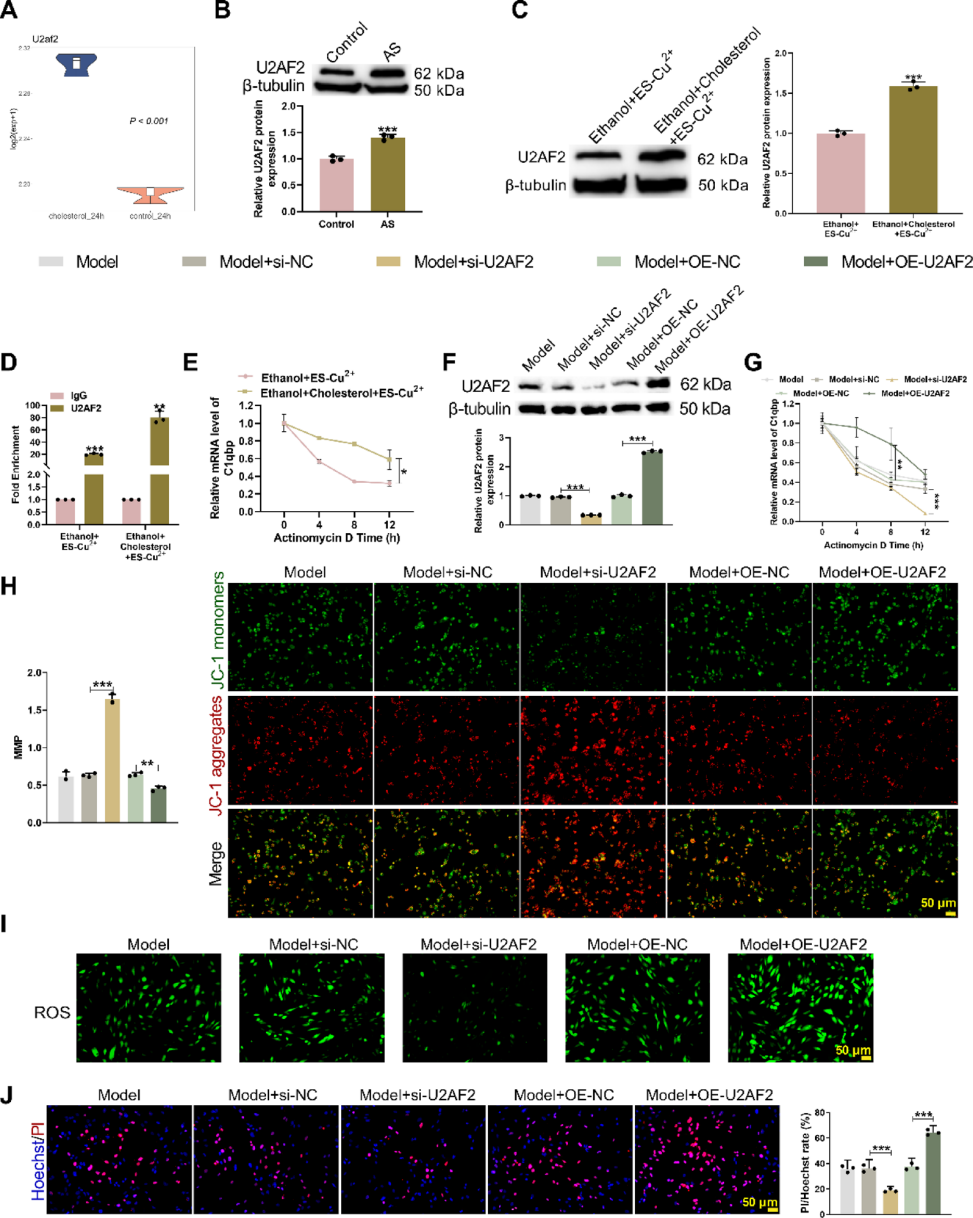
U2AF2 regulates the stability of C1qbp mRNA, affecting cuproptosis. **A** The differentially expressed RNA-binding protein U2AF2 for C1qbp mRNA was identified in GSE174029. ApoE^−/−^ male mice were fed an HFD for 12 weeks. **B** WB detected the expression of U2AF2 in mouse aortas. Mean_Control_ = 1.000; Mean_AS_ = 1.409; 95% CI [0.2908, 0.5272]. The HAVSMC cell line was adopted and treated with Ethanol + Cholesterol + ES-Cu^2+^. **C** WB detected the expression level of U2AF2 in HAVSMC cells. **D** RNA immunoprecipitation (RIP) detected the binding between C1qbp mRNA and the U2AF2 protein. **E** The stability of C1qbp mRNA was detected by actinomycin D. The HAVSMC cell line was jointly treated with cholesterol and ES-Cu^2+^ and si-U2AF2 and OE-U2AF2 were carried out. **F** WB experiment detected the expression of the U2AF2 protein in HAVSMC cells. **G** C1qbp mRNA stability was assessed using actinomycin D. **H** JC-1 staining was used to measure mitochondrial membrane potential in HAVSMC cells. **I** The fluorescent probe DCFH-DA detected ROS levels in HAVSMC cells. **J** Hoechst/PI staining quantified the ratio of red to blue fluorescence intensity in HAVSMC cells. *p < 0.05; **p < 0.01; ***p < 0.001 versus Control/Ethanol + ES-Cu^2+^/IgG/Model + si-NC/Model + OE-NC. NC means negative control of si-U2AF2/OE-U2AF2

Analysis of the GSE174029 dataset identified U2AF2 as a key regulator of C1qbp mRNA (Fig. 3A). To establish an AS model, ApoE^−/−^ mice were fed an HFD for 12 weeks. Western blot and qRT-PCR results confirmed that U2AF2 expression levels were significantly elevated in the AS group (Fig. 3B, Supplementary Fig. 3A). Subsequently, in HAVSMCs, compared to the Ethanol + ES-Cu^2+^ group, the Ethanol + Cholesterol + ES-Cu^2+^ group exhibited significantly higher U2AF2 expression (Fig. 3C, Supplementary Fig. 3B). RIP assays demonstrated that U2AF2 directly binds to C1qbp mRNA (Fig. 3D) and actinomycin D stability assays revealed that Ethanol + Cholesterol + ES-Cu^2+^ treatment enhanced C1qbp mRNA stability (Fig. 3E). In cholesterol-free conditions, U2AF2 regulation experiments were conducted by treating HAVSMC cells with ES-Cu^2+^. ES-Cu^2+^-treated HAVSMC cells were transfected with si-U2AF2 or OE-U2AF2 and knockdown and overexpression efficiency was validated (Supplementary Fig. 6C–F). PCR and WB assays detected U2AF2 and C1qbp expression, while actinomycin D was used to assess C1qbp mRNA stability. Results indicated C1qbp mRNA stability increased following U2AF2 overexpression, suggesting that U2AF2 regulates the stability of C1qbp under copper-depletion conditions without requiring cholesterol exposure (Supplementary Fig. 3C–F). Next, HAVSMCs were co-treated with Cholesterol + ES-Cu^2+^ as Model group. Subsequently, this model group was transfected with si-U2AF2 or OE-U2AF2. Western blot, qRT-PCR and actinomycin D stability assays confirmed that, si-U2AF2 treatment reduced U2AF2 expression and C1qbp mRNA stability, while U2AF2-OE had the opposite effect (Fig. 3F, G, Supplementary Fig. 3G). Functional experiments further showed that, si-U2AF2 improved mitochondrial dysfunction (Fig. 3H), reduced ROS accumulation (Fig. 3I) and decreased cell mortality (Fig. 3J), whereas U2AF2-OE reversed these changes. These results suggest that U2AF2 promotes copper death and AS progression by stabilizing C1qbp mRNA.

### U2AF2 regulates C1qbp mRNA stability via lncRNA NEAT1 scaffolding in AS

**Fig. 4:**
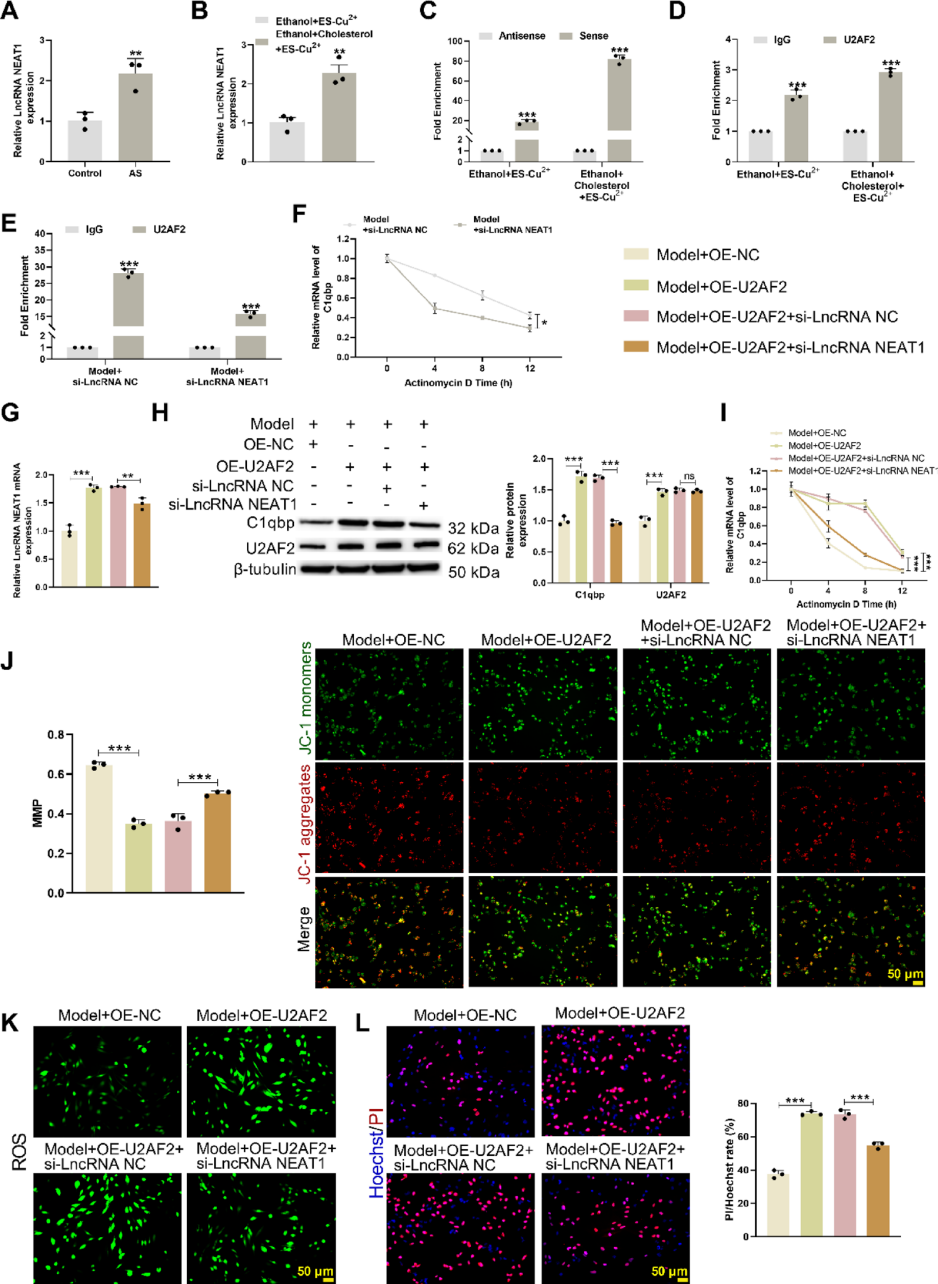
U2AF2 is recruited to C1qbp mRNA via lncRNA NEAT1 to regulate mRNA stability. ApoE^−/−^ male mice were fed an HFD for 12 weeks. **A** qRT-PCR was used to detect the expression level of lncRNA NEAT1 in the aorta of mice. Mean_Control_ = 1.014; Mean_AS_ = 2.171; 95% CI [0.4765, 1.838]. The HAVSMC cell line was selected and treated with Ethanol + Cholesterol + ES-Cu^2+^. **B** qRT-PCR was used to detect the expression level of lncRNA NEAT1 in HAVSMC cells. **C** RNA-RNA pull-down assay was performed to detect the interaction between lncRNA NEAT1 and C1qbp mRNA. **D** RIP assay was conducted to detect the interaction between lncRNA NEAT1 and the U2AF2 protein. The HAVSMC cell line was selected and transfected with si-lncRNA NEAT1. **E** RIP assay was used to detect the binding of C1qbp mRNA to the U2AF2 protein. **F** Actinomycin D treatment was used to assess the stability of C1qbp mRNA. **G** qRT-PCR was used to detect the expression of lncRNA NEAT1. **H** Western blot analysis was performed to detect the expression of U2AF2 and C1qbp. **I** Actinomycin D treatment was used to assess the stability of C1qbp mRNA. **J** JC-1 staining was used to measure mitochondrial membrane potential. **K** Fluorescent probe DCFH-DA was used to detect reactive oxygen species (ROS). **L** Hoechst/PI staining was used to quantify the ratio of red to blue fluorescence intensity in HAVSMCs. *p < 0.05; **p < 0.01; ***p < 0.001 versus Control/Ethanol + ES-Cu^2+^/Antisense/IgG/Model + si-lncRNA NC/Model + OE-NC/Model + OE-U2AF2 + si-lncRNA NC. NC means negative control of OE-U2AF2/si-lncRNA NEAT1

ApoE^−/−^ mice were fed an HFD for 12 weeks to establish an AS model. qRT-PCR results confirmed that lncRNA NEAT1 expression was significantly upregulated in the AS model (Fig. 4A). Subsequently, in HAVSMCs, qRT-PCR results further demonstrated that, compared to the Ethanol + ES-Cu^2+^ group, the Ethanol + Cholesterol + ES-Cu^2+^ group exhibited significantly higher lncRNA NEAT1 expression (Fig. 4B). RNA-RNA pull-down and RIP assays confirmed that lncRNA NEAT1 could simultaneously bind to C1qbp mRNA and U2AF2 protein (Fig. 4C, D), suggesting its role as a molecular scaffold to facilitate U2AF2-mediated regulation of C1qbp mRNA. Next, si-lncRNA NEAT1 was transfected into HAVSMCs and knockdown efficiency was validated (Supplementary Fig. 4A). Mechanistically, RIP and actinomycin D stability assays revealed that si-lncRNA NEAT1 significantly reduced the binding capacity of U2AF2 to C1qbp mRNA (Fig. 4E) and decreased C1qbp mRNA stability (Fig. 4F). Subsequently, OE-U2AF2 or/and si-lncRNA NEAT1 vectors were transfected into HAVSMCs. Western blot, qRT-PCR and actinomycin D stability assays confirmed that, OE-U2AF2 treatment enhanced the expression and stability of lncRNA NEAT1, U2AF2 and C1qbp expression, while OE-U2AF2 + si-lncRNA NEAT1 treatment reversed these effects, with no significant change in U2AF2 expression (Fig. 4G–I, Supplementary Fig. 4B, C). Functional experiments further showed that OE-U2AF2 promoted mitochondrial dysfunction (Fig. 4J), increased reactive oxygen species (ROS) accumulation (Fig. 4K) and elevated cell mortality (Fig. 4L), whereas OE-U2AF2 + si-lncRNA NEAT1 ameliorated these phenotypes. These results indicate that U2AF2 enhances C1qbp mRNA stability by recruiting lncRNA NEAT1, thereby promoting AS progression.

### U2AF2 knockdown stabilizes plaques and attenuates atherosclerosis progression

**Fig. 5:**
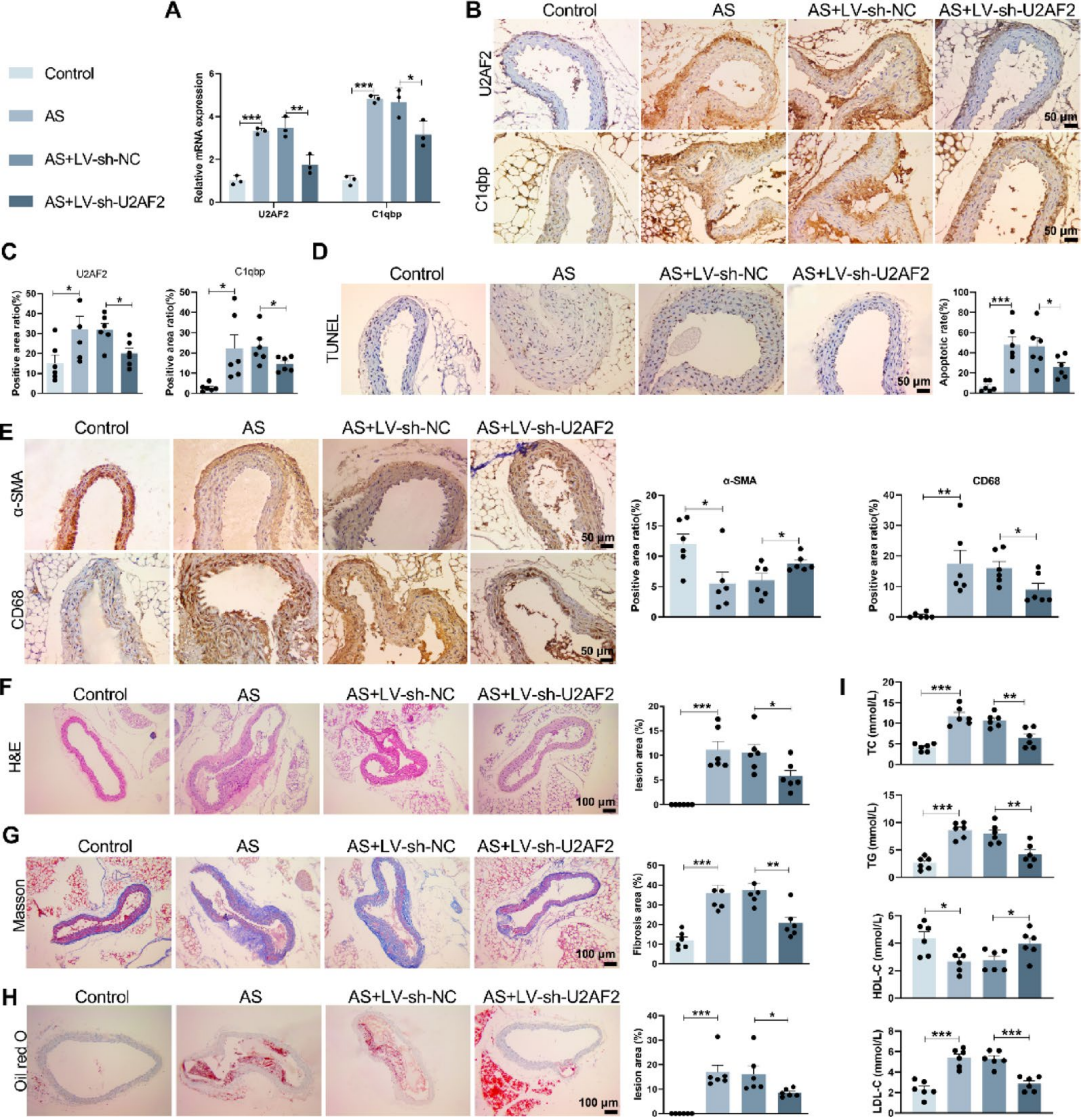
Knockdown of U2AF2 has a stabilizing effect on plaques and delays the progression of atherosclerosis. ApoE^−/−^ male mice were injected with lentivirus carrying sh-U2AF2 via the tail vein, followed by an HFD for 12 weeks after 4 weeks. Aortic tissues were collected for subsequent experiments. **A** qRT-PCR was used to detect the expression of U2AF2 and C1qbp mRNA in aortic tissues. U2AF2: Mean_Control_ = 1.015; Mean_AS_ = 3.319; Mean_AS+LV-sh-NC_ = 3.480; Mean_AS+LV-sh-U2AF2_ = 1.748; 95% CI_Control/AS_ [− 3.245, 3.264]; 95% CI_AS+LV-sh-NC/AS+LV-sh-U2AF2_ [0.7917, 2.673]; C1qbp: Mean_Control_ = 1.016; Mean_AS_ = 4.814; Mean_AS+LV-sh-NC_ = 4.691; Mean_AS+LV-sh-U2AF2_ = 3.163; 95% CI_Control/AS_ [− 5.030, − 2.566]; 95% CI_AS+LV-sh-NC/AS+LV-sh-U2AF2_ [0.2966, 2.761]. **B**, **C** Immunohistochemistry was performed to detect the protein expression of U2AF2 and C1qbp in aortic tissues (200X). U2AF2: Mean_Control_ = 15.37; Mean_AS_ = 32.36; Mean_AS+LV-sh-NC_ = 31.96; Mean_AS+LV-sh-U2AF2_ = 20.04; 95% CI_Control/AS_ [0.4048, 33.56]; 95% CI_AS+LV-sh-NC/AS+LV-sh-U2AF2_ [− 21.06, − 2.773]; C1qbp: Mean_Control_ = 2.739; Mean_AS_ = 22.2; Mean_AS+LV-sh-NC_ = 23.20; Mean_AS+LV-sh-U2AF2_ = 14.55; 95% CI_Control/AS_ [4.433, 34.43]; 95% CI_AS+LV-sh-NC/AS+LV-sh-U2AF2_ [− 17.14, − 0.1504]. **D** TUNEL staining was used to show dead cells in the fibrous cap of the plaque surface. Mean_Control_ = 6.432; Mean_AS_ = 47.72; Mean_AS+LV-sh-NC_ = 46.46; Mean_AS+LV-sh-U2AF2_ = 25.77; 95% CI_Control/AS_ [22.21, 60.36]; 95% CI_AS+LV-sh-NC/AS+LV-sh-U2AF2_ [− 41.35, − 0.048]. **E** Immunohistochemistry was used to detect the expression of α-SMA and CD68 in aortic tissues (200X). α-SMA: Mean_Control_ = 12.01; Mean_AS_ = 5.505; Mean_AS+LV-sh-NC_ = 6.047; Mean_AS+LV-sh-U2AF2_ = 8.808; 95% CI_Control/AS_ [− 12, − 1.011]; 95% CI_AS+LV-sh-NC/AS+LV-sh-U2AF2_ [0.0090, 5.514]; CD68: Mean_Control_ = 0.525; Mean_AS_ = 17.44; Mean_AS+LV-sh-NC_ = 16.03; Mean_AS+LV-sh-U2AF2_ = 8.998; 95% CI_Control/AS_[6.959, 26.87]; 95% CI_AS+LV-sh-NC/AS+LV-sh-U2AF2_ [− 13.70, − 0.3661]. **F** HE staining was used to observe atherosclerotic plaques and foam cells in aortic tissues. Mean_Control_ = 0; Mean_AS_ = 11.13; Mean_AS+LV-sh-NC_ = 10.59; Mean_AS+LV-sh-U2AF2_ = 5.783; 95% CI_Control/AS_ [7.233, 15.02]; 95% CI_AS+LV-sh-NC/AS+LV-sh-U2AF2_ [− 9.37, − 0.24]. **G** Masson’s trichrome staining was used to measure collagen content. Mean_Control_ = 12.02; Mean_AS_ = 35.90; Mean_AS+LV-sh-NC_ = 37.44; Mean_AS+LV-sh-U2AF2_ = 20.70; 95% CI_Control/AS_ [13.82, 33.94]; 95% CI_AS+LV-sh-NC/AS+LV-sh-U2AF2_ [− 26.71, − 6.775]. **H** Oil Red O staining was performed to analyze lipid accumulation in the plaques. Mean_Control_ = 0; Mean_AS_ = 16.88; Mean_AS+LV-sh-NC_ = 16.22; Mean_AS+LV-sh-U2AF2_ = 8.845; 95% CI_Control/AS_ [9.303, 24.45]; 95% CI_AS+LV-sh-NC/AS+LV-sh-U2AF2_ [− 16.13, 0.6699]. **I** Kits were used to detect the levels of TC, TG, LDL-C and HDL-C in plasma. HDL-C: Mean_Control_ = 4.355; Mean _AS_ = 2.658; Mean_AS+LV-sh-NC_ = 2.753; Mean _AS+LV-sh-U2AF2_ = 3.952; 95% CI_Control/AS_ [− 2.939, − 0.4539]; 95% CI_AS+LV-sh-NC/AS+LV-sh-U2AF2_ [− 0.551, 2.342]; LDL-C: Mean_Control_ = 2.325; Mean_AS_ = 5.388; Mean_AS+LV-sh-NC_ = 5.287; Mean_AS+LV-sh-U2AF2_ = 2.865; 95% CI_Control/AS_ [1.962, 4.165]; 95% CI_AS+LV-sh-NC/AS+LV-sh-U2AF2_ [− 3.365, − 1.487]; TC: Mean_Control_ = 4.147; Mean_AS_ = 11.73; Mean_AS+LV-sh-NC_ = 10.72; Mean_AS+LV-sh-U2AF2_ = 6.462; 95% CI_Control/AS_ [5.343, 9.814]; 95% CI_AS+LV-sh-NC/AS+LV-sh-U2AF2_ [− 6.859, − 1.655]; TG:Mean_Control_ = 2.608; Mean_AS_ = 8.608; Mean_AS+LV-sh-NC_ = 7.963; Mean_AS+LV-sh-U2AF2_ = 4.275; 95% CI_Control/AS_ [4.362, 7.638]; 95% CI_AS+LV-sh-NC/AS+LV-sh-U2AF2_ [− 6.039, − 1.338]. *p < 0.05; **p < 0.01; ***p < 0.001 versuss Control/AS + LV-sh-NC. NC means negative control of LV-sh-U2AF2

An AS model was established using ApoE^−/−^ mice, ApoE^−/−^ Male mice were injected with sh-U2AF2 lentivirus via tail vein and knockdown efficiency was validated (Supplementary Fig. 6G, H). qRT-PCR and immunohistochemistry assays revealed that, compared to the Control group, U2AF2 and C1qbp expression levels were significantly elevated in the AS group, while LV-sh-U2AF2 treatment reversed these trends (Fig. 5A–C). TUNEL staining further demonstrated that apoptosis was reduced in the AS + LV-sh-U2AF2 group compared to the AS + LV-sh-NC group (Fig. 5D). Immunohistochemistry showed that LV-sh-U2AF2 treatment reduced CD68 macrophage infiltration and increased α-SMA smooth muscle cell content in plaques (Fig. 5E). Furthermore, immunofluorescence double-staining experiments revealed colocalization between CD68 and α-SMA (Supplementary Fig. 5). HE, Masson and Oil Red O staining analyses indicated that, compared to the Control group, the AS group exhibited thickened vascular walls (Fig. 5F), increased collagen deposition (Fig. 5G) and increased lipid accumulation in plaques (Fig. 5H), whereas AS + LV-sh-U2AF2 treatment ameliorated these changes. Additionally, compared to the Control group, serum levels of TC, TG and LDL-C were significantly elevated and HDL-C was reduced in the AS group; AS + LV-sh-U2AF2 treatment reversed these effects (Fig. 5I). These results demonstrate that targeted silencing of U2AF2 significantly improves AS and delays disease progression.

## Discussion

AS, as the primary pathological foundation of atherosclerosis-related cardiovascular and cerebrovascular diseases, involves complex and diverse mechanisms, including lipid metabolism disorders, inflammatory responses, oxidative stress and cell death [21]. In recent years, the role of copper death in AS has gradually gained attention. This study systematically revealed the critical role of copper death in AS development using an HFD-induced ApoE^−/−^ mice model and explored the molecular mechanisms of the C1qbp-DLAT axis and the U2AF2-NEAT1 regulatory network [22]. Although copper death, as a novel form of regulated cell death, has been extensively studied in oncology, its role in atherosclerosis-related cardiovascular and cerebrovascular diseases remains unclear.

In this study, we first demonstrated that copper ion levels were significantly elevated in the AS model, with abnormal expression of copper death related genes. The copper ion carrier ES-Cu^2+^ induced copper death in HAVSMCs, accompanied by increased ROS and mitochondrial dysfunction. Our findings revealed that serum copper levels were significantly elevated in AS patients and ApoE^⁻/⁻^ mice and excessive copper accumulation accelerated plaque formation by promoting inflammation and oxidative stress [4]. Additionally, copper generates ROS through the Fenton reaction, leading to endothelial cell damage and lipid peroxidation, further exacerbating AS [23]. Further studies showed that C1qbp regulated copper death by promoting the oligomerization modification of DLAT protein, while U2AF2 enhanced this process by stabilizing C1qbp mRNA. LncRNA NEAT1 acted as a molecular scaffold to recruit U2AF2 to C1qbp mRNA, forming a regulatory axis. U2AF2 interacted with hnRNP U and hnRNP L, playing a crucial role in splicing regulation. Moreover, U2AF2 stabilizes specific mRNAs (HMGCR and EXO1) by binding to lncRNAs (ZFAS1 and CECR7), thereby affecting tumor development [24]. Finally, targeted inhibition of U2AF2 significantly improved AS pathological features and delayed disease progression. These findings provide new therapeutic targets and theoretical support for AS treatment.

Furthermore, this study is the first to systematically elucidate the activation of copper death in AS and its pathogenic mechanisms. Elevated copper ion levels and upregulated expression of copper death-related genes (SLC31A1) were observed in the AS model, while key proteins involved in copper metabolism (DLAT, ATP7A) were downregulated, indicating that copper homeostasis imbalance is a hallmark of AS. Previous studies have shown that SLC31A1 knockout models exhibit mitochondrial copper depletion, but its upregulation in AS exacerbates copper toxicity [10]. The copper ion carrier ES-Cu^2+^ significantly induced copper death in HAVSMCs, characterized by reduced cell proliferation, increased apoptosis, elevated ROS levels, and impaired mitochondrial function [25]. These findings align with prior reports on copper-induced cytotoxicity [26], but our study further linked copper death directly to AS pathological features such as plaque formation and lipid deposition, revealing its specific role in AS progression. Our study indicated that Hoechst/PI staining revealed diffuse nuclear PI uptake, distinct from apoptotic nuclear condensation or pyroptotic membrane blebbing, supporting cuproptosis as the predominant death mode. However, copper-induced ROS may interact with pyroptosis through NLRP3 activation, thereby exacerbating plaque inflammation [2]. Although our data showed that DLAT oligomerization was a key marker of copper death, its specificity in atherosclerosis and other copper overload diseases still requires further investigation. Furthermore, whether cell death initiation shifts from cuproptosis to secondary necrosis/pyroptosis in advanced plaques requires lineage-tracing studies.

Second, under cholesterol stimulation, C1qbp expression was significantly upregulated and promoted the oligomerization modification of DLAT protein through direct binding, leading to reduced DLAT protein stability and activity. As a key enzyme in mitochondrial copper metabolism, DLAT's oligomerization may impair copper ion transport and metabolism, thereby exacerbating copper death [20]. This mechanism explains the abnormal expression of copper death-related genes in AS and highlights the pivotal role of C1qbp in regulating copper death. Additionally, knockdown of C1qbp reversed copper death phenotypes, further confirming the functionality of this axis. Studies showed that in AS cell models, C1qbp knockdown reduced DLAT enzyme activity, decreased copper-dependent protein aggregation, increased MRE11 degradation and impaired DNA repair capacity, but unexpectedly lowered copper death sensitivity [27]. U2AF2, as an RNA-binding protein, enhanced C1qbp mRNA stability by directly binding to it, while lncRNA NEAT1 recruited U2AF2 to C1qbp mRNA as a molecular scaffold, forming a positive feedback loop. This regulatory network was significantly activated in AS, characterized by elevated U2AF2 and NEAT1 expression, which correlated with increased C1qbp mRNA stability. Research indicated that U2AF2 directly binds to the 3'UTR or specific domains of C1qbp mRNA in AS progression, inhibiting the access of degradation enzymes (exosome complex) and enhancing mRNA stability [26, 28]. Targeted inhibition of U2AF2 or NEAT1 significantly reduced C1qbp expression, alleviated copper death related mitochondrial dysfunction and cell death, highlighting the central role of this network in AS pathogenesis.

Although the association between copper ions and atherosclerosis-related cardiovascular and cerebrovascular diseases has been reported, the specific role and mechanism of copper in AS remained unclear before this study. This research is the first to directly link copper death to pathological features of AS, such as plaque formation, lipid deposition, apoptosis and elucidate the intrinsic mechanisms of copper death-related gene expression changes. Unlike previous studies focusing on traditional cell death pathways (apoptosis, necrosis), this work provides a novel perspective on AS pathogenesis. C1qbp, a newly discovered regulator of copper death, modulates copper death by promoting DLAT oligomerization, representing an innovative mechanism [29]. While prior research primarily emphasized DLAT's enzymatic activity in copper metabolism, this study revealed its non-enzymatic role in oligomerization during copper death, expanding the understanding of DLAT’s functions. DLAT oligomerization is a key molecular mechanism of copper death. Copper ions promote pathological DLAT oligomerization through direct binding to lipoylated DLAT, resulting in proteotoxic stress and subsequent cell death [30]. Finally, the ApoE^−/−^ mouse AS model was constructed. In the sh-U2AF2 treatment group, the infiltration of CD68 macrophages and the content of α-SMA smooth muscle cells decreased in the plaques of mice. Studies have shown that in HUVECs treated with ox-LDL, the expression of α-SMA is significantly upregulated, while USF1 knockout or USP14 inhibition can reverse this upregulation trend [31]. This study provided the first demonstration that U2AF2 promotes cuproptosis through stabilizing C1qbp mRNA, and revealed a novel regulatory mechanism whereby lncRNA NEAT1 serves as a molecular scaffold to recruit U2AF2. Mechanistically, U2AF2 bound to polypyrimidine tracts within the 3′UTR of C1qbp mRNA, thereby protecting it from degradation and prolonging its half-life [24]. This finding not only explains the enhanced stability of C1qbp mRNA but also adds new complexity to the molecular regulatory network of AS. Unlike previous studies where lncRNAs mainly functioned as competing endogenous RNAs (ceRNAs), this research reveals NEAT1's structural role in RNA–protein interactions.

This study utilized ApoE^−/−^ mice to establish an AS model, which could mimic the basic characteristics of human AS but could not fully replicate the complexity of the disease. However, this research still has some limitations. Future studies should combine other animal models (Western diet-induced AS models) or in vitro human samples for validation [32]. U2AF2 and NEAT1, as potential therapeutic targets, require extensive research to develop specific inhibitors and assess their safety. It is necessary to integrate transcriptomics, proteomics and metabolomics sequencing to systematically dissect the overall regulatory network of copper death in AS and identify more potential targets [33]. Exploring the synergistic effects of copper death with apoptosis, proptosis and other cell death pathways in AS will reveal a more comprehensive pathological mechanism. Future research can further expand these aspects to provide more effective clinical treatment strategies.

## Conclusion

This study systematically elucidates a novel mechanism by which the RNA-binding protein U2AF2 promotes AS progression through post-transcriptional regulation mediated by lncRNA NEAT1 in VSMCs. The research found that under pathological conditions of AS, U2AF2 forms a molecular complex with lncRNA NEAT1, stabilizing C1qbp mRNA and thereby upregulating C1qbp expression. Elevated C1qbp protein directly binds to the key copper death related protein DLAT, inducing its oligomerization and ultimately triggering the copper death program in VSMCs. Animal experiments confirmed that targeted inhibition of U2AF2 significantly reduced aortic plaque area, improved lipid metabolism and enhanced plaque stability. This study is the first to clarify the role of the “U2AF2-NEAT1-C1qbp-DLAT” regulatory axis in AS, providing not only a new perspective for understanding VSMC death but also potential therapeutic targets for precise AS treatment, particularly intervention strategies targeting the copper death pathway.

## Supplementary Information

Below is the link to the electronic supplementary material.

Supplementary Material 1

Supplementary Material 2: Fig. S1. Increased Cuproptosis in the AS model. ApoE^−/−^ male mice were fed an HFD for 12 weeks and the aorta was collected for subsequent experiments. (A) Masson’s trichrome staining was used to measure collagen content. Mean_Control_=9.453; Mean_AS_=22.27; 95%CI[5.498, 20.13]. (B) TUNEL staining was performed to show dead cells in the fibrous cap of the plaque surface. ApoE^−/−^ male mice were fed an HFD for 12 weeks and treated with Ethanol + Cholesterol + ES-Cu^2+^. Mean_Control_=7.353; Mean_AS_=49.87; 95%CI[20.86, 64.18]. (C) Multiplex immunofluorescence for detecting CD68 and α-SMA in the abdominal aorta. Verification experiment demonstrating that the addition of ethanol has no effect on cells. (D) CCK8 assay was used to detect proliferation. (E-F) The TUNEL assay was used to detect apoptosis. HAVSMCs were selected and treated with the copper ionophore ES-Cu^2+^. (G-H) JC-1 staining was used to analyze mitochondrial membrane potential. (I) qRT-PCR was performed to detect the expression of DLAT and SLC31A1. *p < 0.05, **p < 0.01, ***p < 0.001 versus Control/Ctrl/Ctrl + Ethanol/Ethanol + ES-Cu^2+^/Ethanol/Ethanol + Cholesterol. ns means no significant difference versus Ctrl/ES-Cu^2+^

Supplementary Material 3: Fig. S2. Knocking down C1qbp affects DLAT expression. HAVSMC cells were treated with ethanol, ethanol + cholesterol, ethanol + ES-Cu^2+^, and ethanol + cholesterol + ES-Cu^2+^, respectively. (A) qRT-PCR was used to detect the expression level of C1qbp mRNA. (B) WB was performed to detect DLST and Lipo-DLST protein expression. HAVSMC cells were co-treated with cholesterol and ES-Cu^2+^, followed by transfection with si-C1qbp. (C) qRT-PCR was performed to detect the expression levels of DLAT and C1qbp mRNA. (D) WB was used to detect DLST and Lipo-DLST protein expression. *p < 0.05, **p < 0.01, ***p < 0.001 versus Ethanol/Ethanol + Cholesterol/Ethanol + ES-Cu^2+^/Model + si-NC. NC means negative control of si-C1qbp

Supplementary Material 4: Fig. S3. U2AF2 was increased in atherosclerosis. ApoE^−/−^ male mice were fed an HFD for 12 weeks. **A** qRT-PCR was used to detect the expression of U2AF2 in the aortic tissues of the mice. Mean_Control_ = 1.009; Mean_AS_ = 2.076; 95% CI [0.7757, 1.358]. The HAVSMC cell line was then selected and subsequently treated with Ethanol + Cholesterol + ES-Cu^2+^. **B** qRT-PCR was used to detect the expression of U2AF2 in HAVSMC cells. HAVSMC cells were treated with ES-Cu^2+^, followed by si-NC, si-U2AF2, OE-NC and OE-U2AF2 treatments. **C** qRT-PCR detected the expression of U2AF2 and C1qbp in HAVSMC cells. **D**, **E** WB detection of U2AF2 and C1qbp protein expression. **F** Actinomycin D detection of C1qbp mRNA stability. HAVSMC cells were co-treated with cholesterol and ES-Cu^2+^, followed by si-U2AF2 and OE-U2AF2 treatments. **G** qRT-PCR was used to detect the mRNA expression of U2AF2 in HAVSMC cells. *p < 0.05, **p < 0.01, ***p < 0.001 versus Control/Ethanol + ES-Cu^2+^/ES-Cu^2+^+si-NC/ES-Cu^2+^+OE-NC/Model + si-NC/Model + OE-NC. NC means negative control of si-U2AF2/OE-U2AF2

Supplementary Material 5: Fig. S4. U2AF2 recruits to C1qbp mRNA via LncRNA NEAT1 to regulate mRNA stability. The HAVSMC cell line was selected and subsequently treated with si-lncRNA NEAT1. **A** qRT-PCR was used to detect the knockdown efficiency of lncRNA NEAT1 in HAVSMC cells. The HAVSMC cell line was then selected and subsequently treated with OE-U2AF2 and OE-U2AF2 + si-lncRNA NEAT1. **B**, **C** qRT-PCR was used to detect the expression of U2AF2 and C1qbp in HAVSMC cells. *p < 0.05, **p < 0.01, ***p < 0.001 versus Model + si-lncRNA NC/Model + OE-NC/Model + OE-U2AF2 + si-lncRNA NC. NC means negative control of OE-U2AF2/si-lncRNA NEAT1

Supplementary Material 6: Fig. S5. CD68 and α-SMA Co-localization detection. Immunofluorescence double staining was used to detect the colocalization of CD68 and α-SMA in aortic tissues from the Control, AS, AS + LV-sh-NC, and AS + LV-sh-U2AF2 groups. NC means negative control of sh-U2AF2

Supplementary Material 7: Fig. S6. Efficiency verification. **A**,** B** qRT-PCR and Western blot analysis of C1qbp expression in the si-NC, si-C1qbp-1, and si-C1qbp-2 groups. **C**, **D** qRT-PCR and Western blot analysis of U2AF2 expression in the si-NC, si-U2AF2 -1, si-U2AF2 -2 groups. (E-F) qRT-PCR and Western blot analysis of U2AF2 expression in the OE-NC and OE-U2AF2 groups. **G**, **H** qRT-PCR and WB analysis of U2AF2 expression in the sh-NC, sh-U2AF2-1, sh-U2AF2 -2 groups. *p < 0.05, **p < 0.01, ***p < 0.001 versus sh-NC/si-NC/OE-NC. NC means negative control of si-C1qbp/si-U2AF2/OE-U2AF2/sh-U2AF2

## Data Availability

All data generated and/or analyzed during the current study are available from the corresponding author on reasonable request.

## Abbreviations

AS: Atherosclerosis
CO-IP: Co-immunoprecipitation
C1qBP: Complement component 1Q subcomponent-binding protein
DLAT: Dihydrolipoamide S-acetyltransferaseDihydrolipoamide acyltransferase
DLST: Dihydrolipoamide S-succinyltransferase
DMEM: Dulbecco’s Modified Eagle Medium
ES-Cu^2+^: Elesclomol-Cu^2+^
HFD: High-fat diet
LDL: Low-density lipoprotein
LDL-C: Low-density lipoprotein cholesterol
HAVSMCs: Human aortic vascular smooth muscle cells
HDL-C: High-density lipoprotein cholesterol
OXPHOS: Oxidative phosphorylation
qRT-PCR: Quantitative real-time polymerase chain reaction
ROS: Reactive oxygen species
SPF: Specific pathogen-free
TC: Total cholesterol
TCA: Tricarboxylic acid cycle
TG: Total triglyceride
TUNEL: Terminal deoxynucleotidyl transferase dUTP nick end labeling
U2AF2: U2 small nuclear RNA auxiliary factor 2
VSMCs: Vascular smooth muscle cells
WB: Western blotting
