# Supplementary material for: Vascular smooth muscle cell RNA-binding protein U2AF2 induces copper death by regulating C1qbp expression, delaying development of atherosclerosise
Source: Biol Res. 2026 Jan 28;59:13. doi: 10.1186/s40659-026-00672-3 (PMC12924209; doi:10.1186/s40659-026-00672-3)
Supplement: Supplementary file 1 — Supplementary Material 1 [file 40659_2026_672_MOESM1_ESM.pptx]

## Slide 1
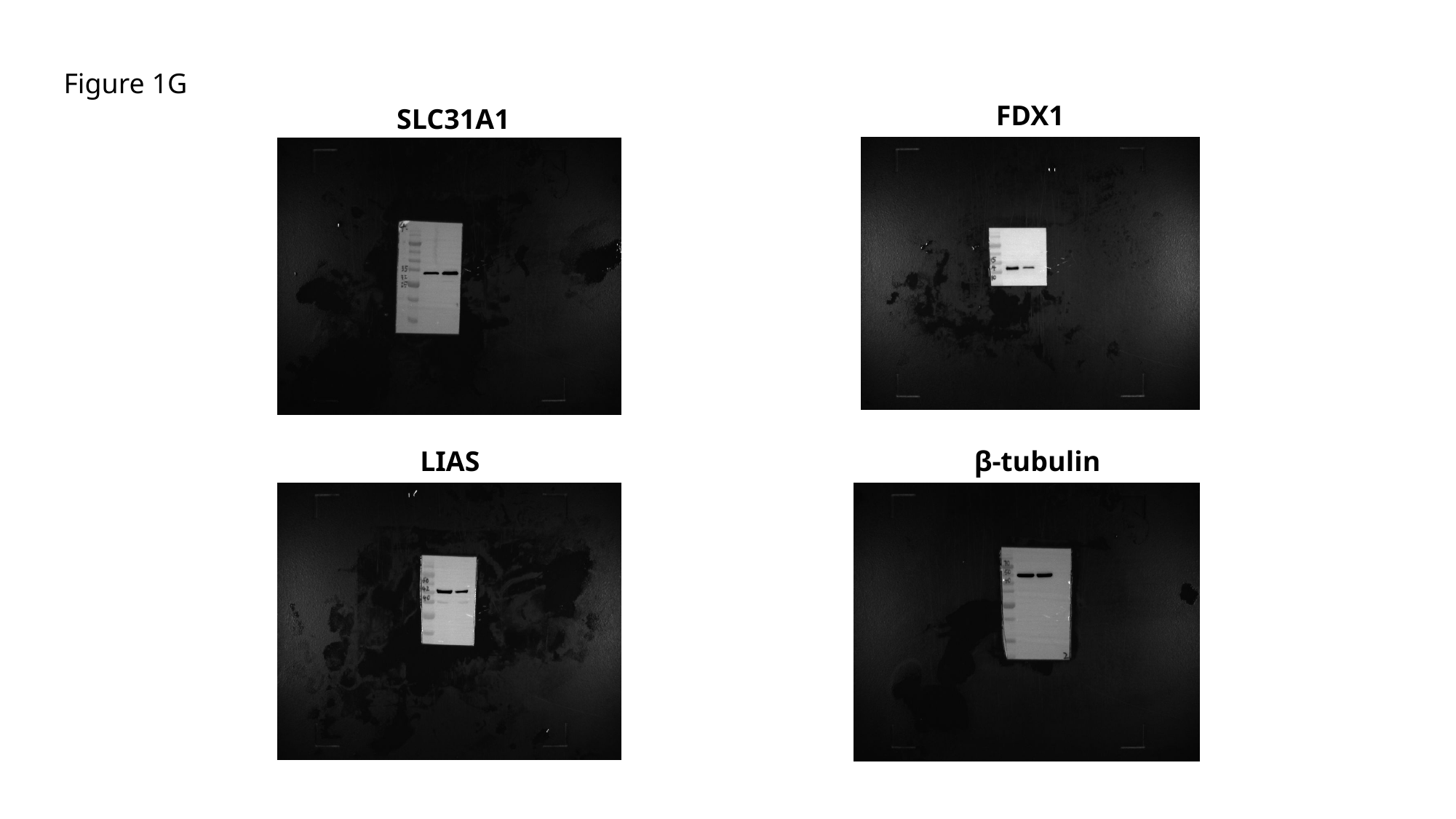

Figure 1G
FDX1
SLC31A1
LIAS
β-tubulin

## Slide 2
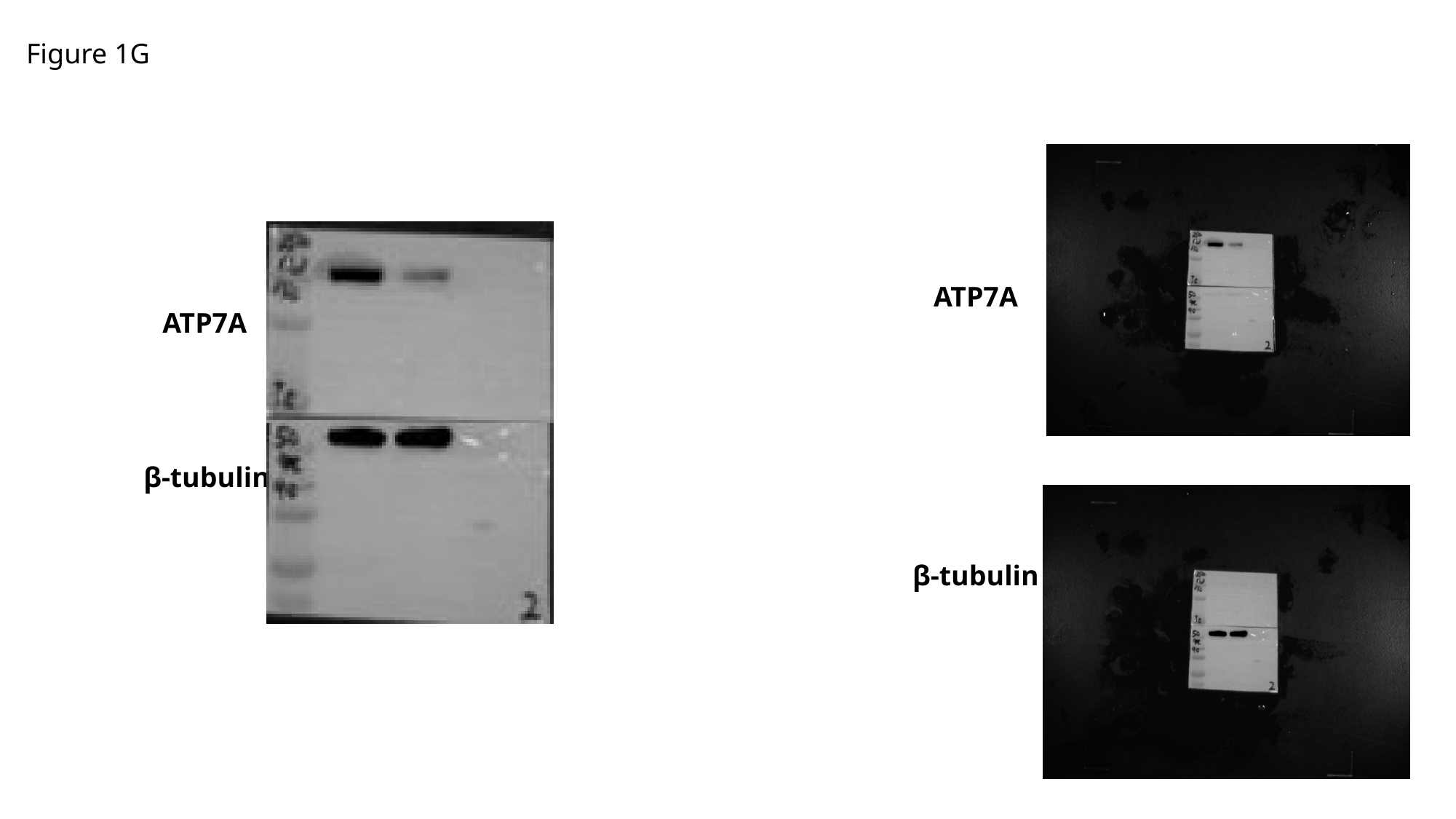

Figure 1G
ATP7A
ATP7A
β-tubulin
β-tubulin

## Slide 3
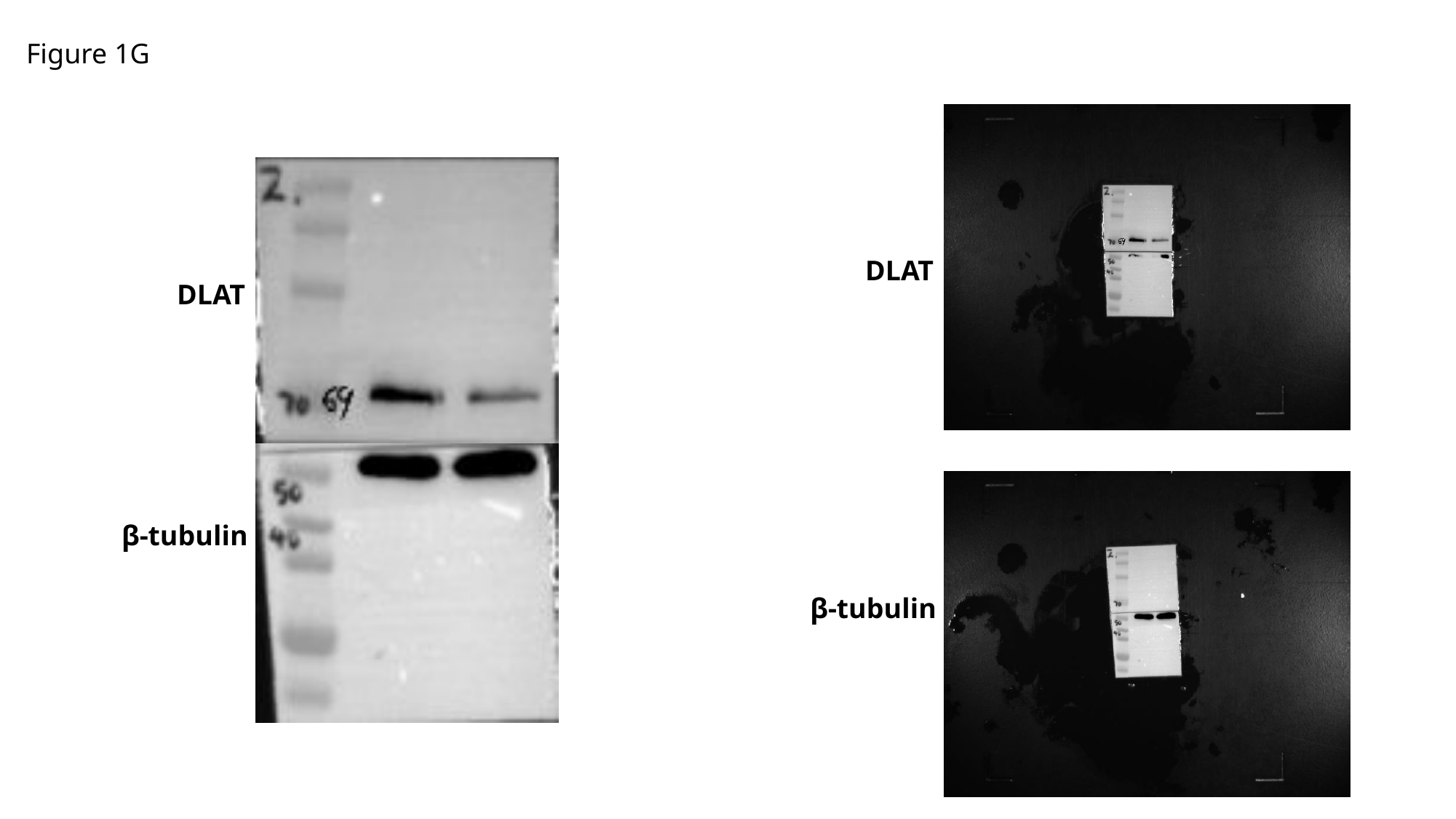

Figure 1G
DLAT
DLAT
β-tubulin
β-tubulin

## Slide 4
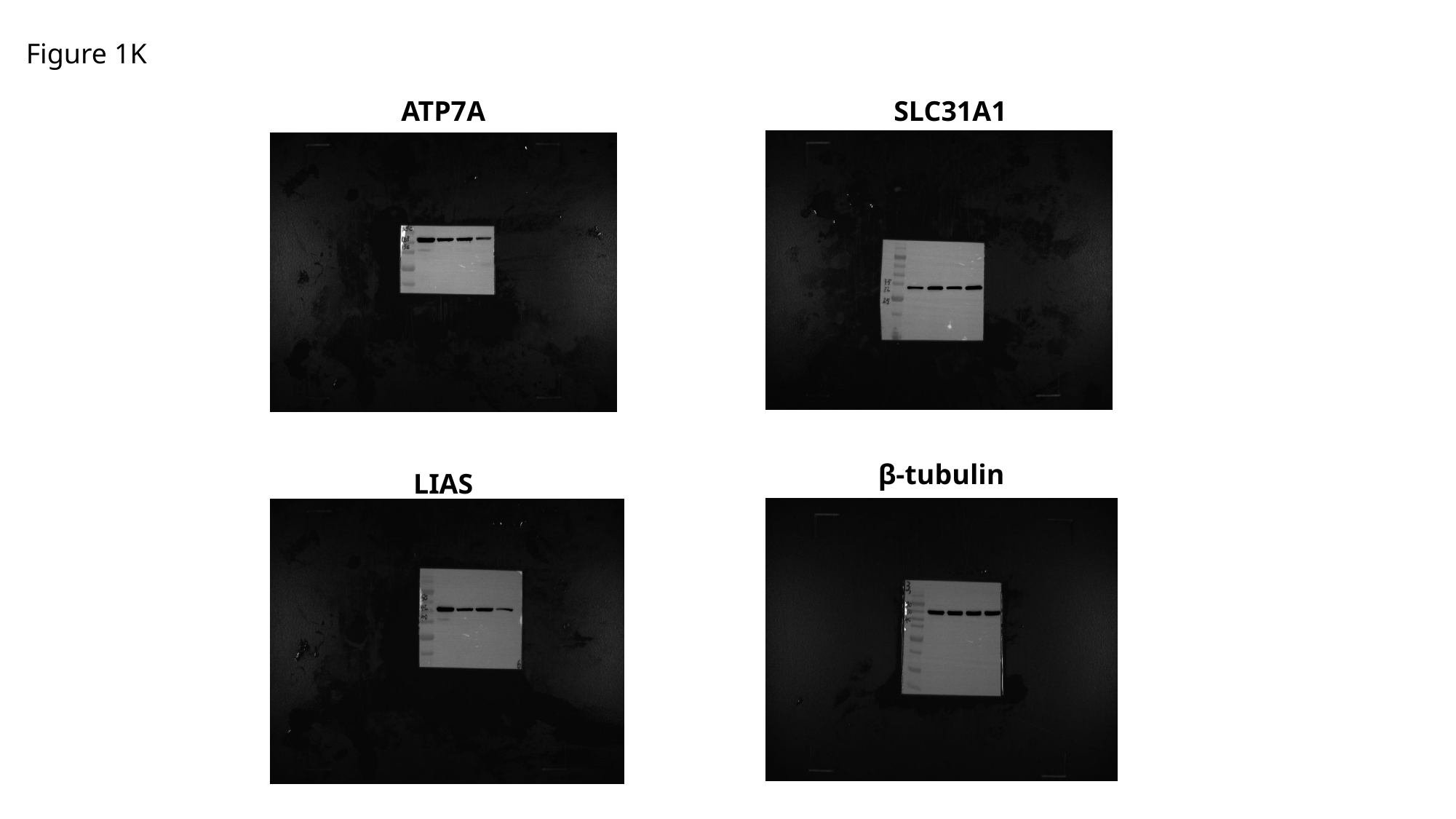

Figure 1K
SLC31A1
ATP7A
β-tubulin
LIAS

## Slide 5
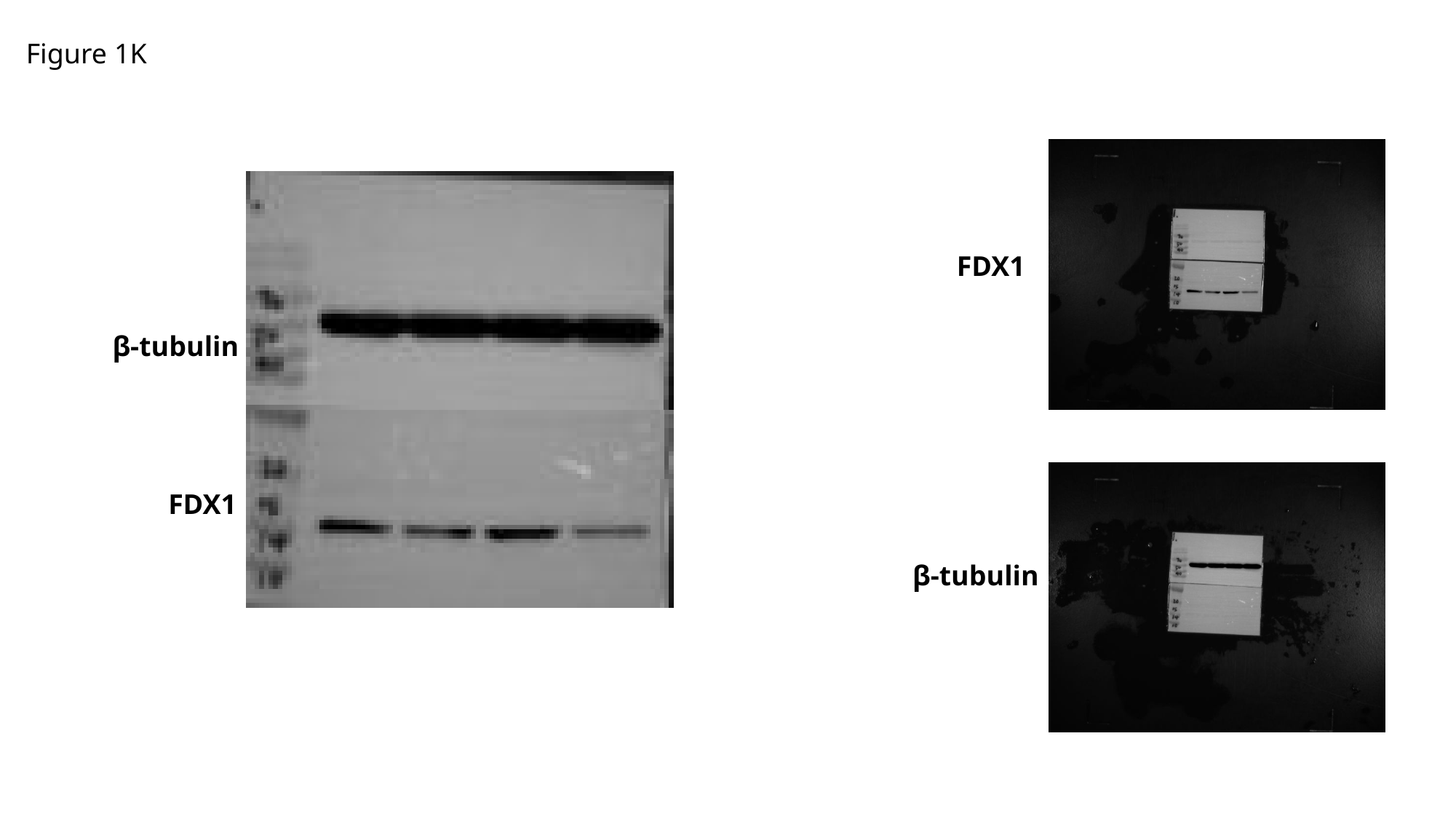

Figure 1K
FDX1
β-tubulin
FDX1
β-tubulin

## Slide 6
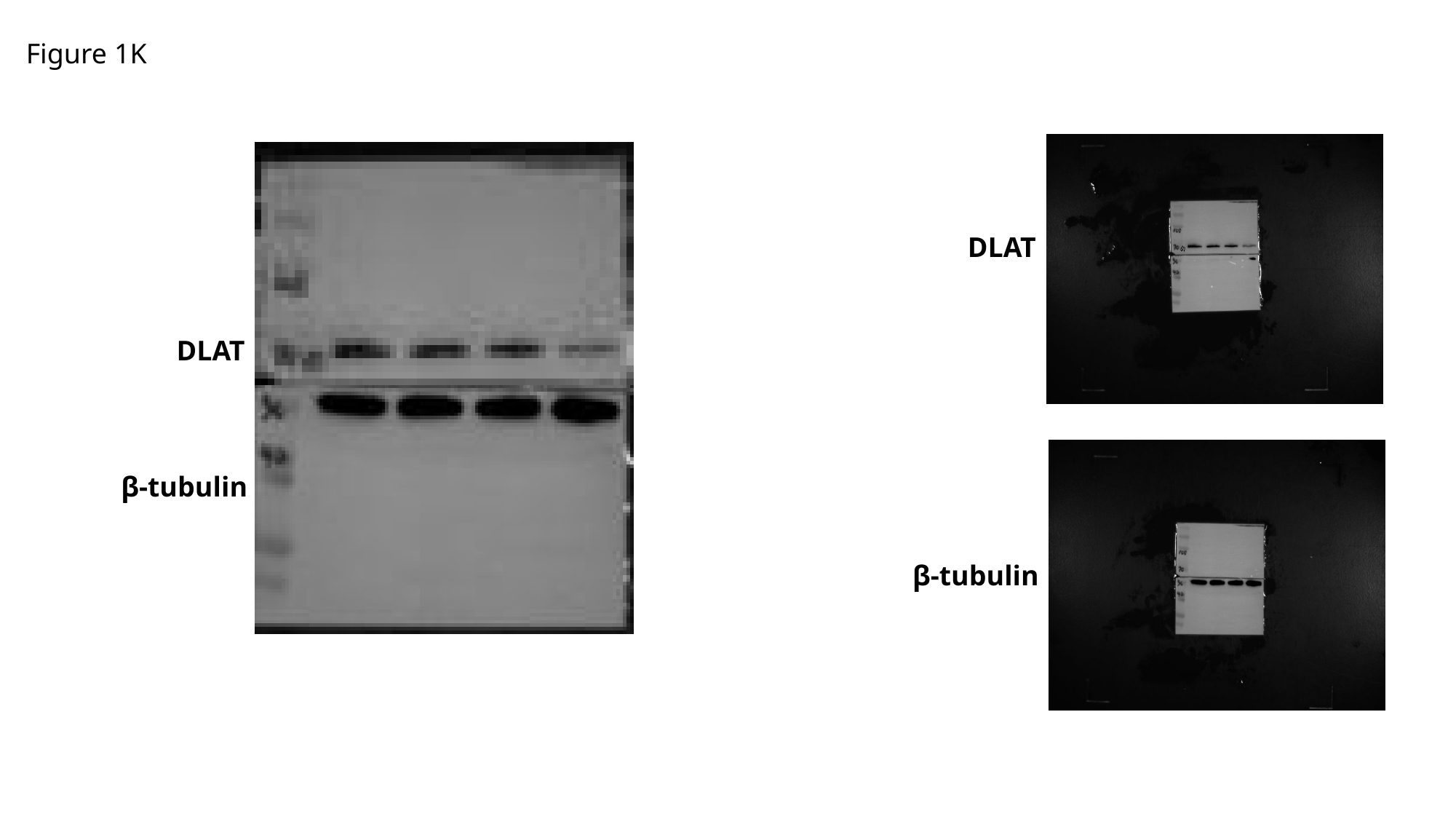

Figure 1K
DLAT
DLAT
β-tubulin
β-tubulin

## Slide 7
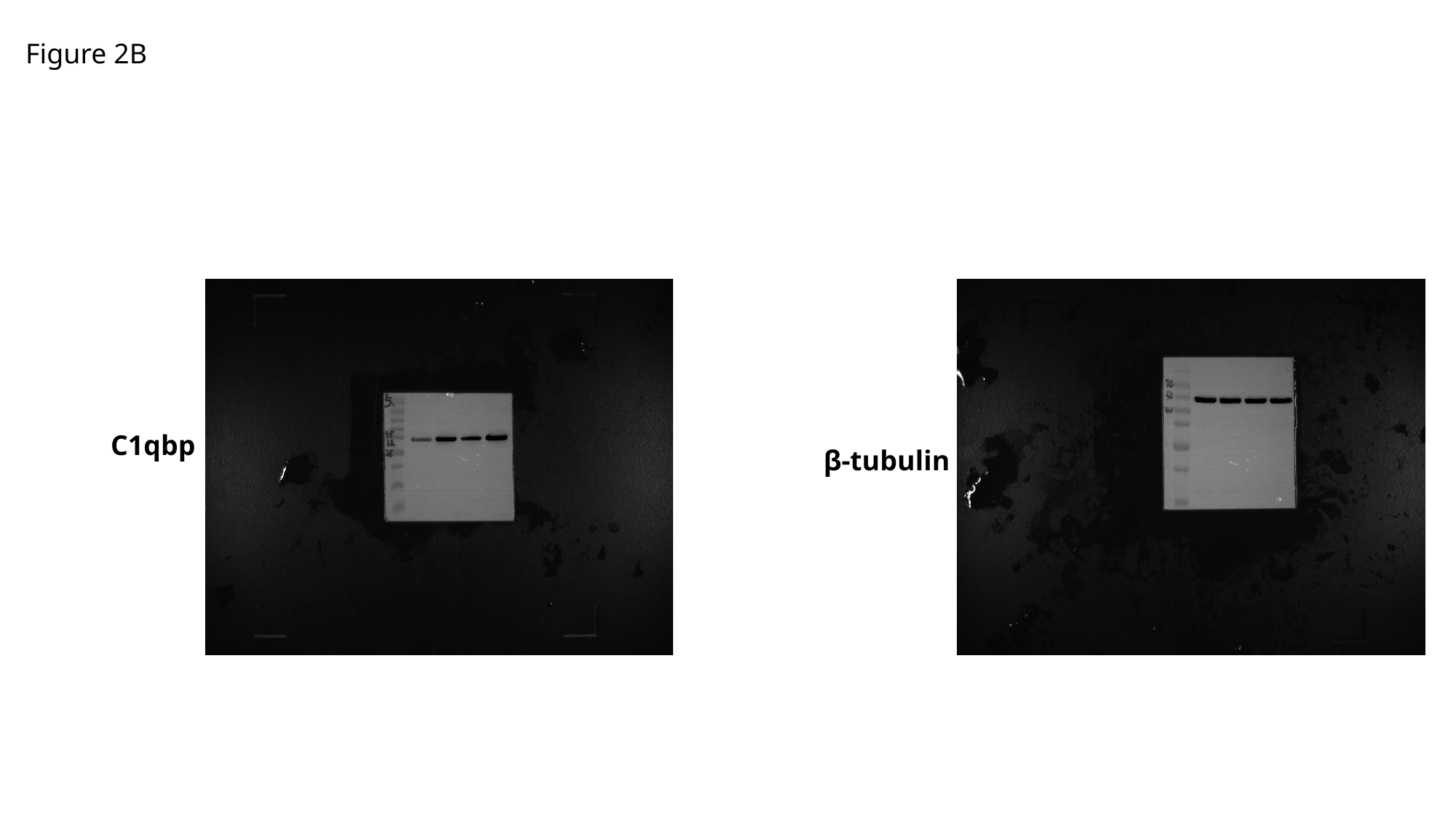

Figure 2B
C1qbp
β-tubulin

## Slide 8
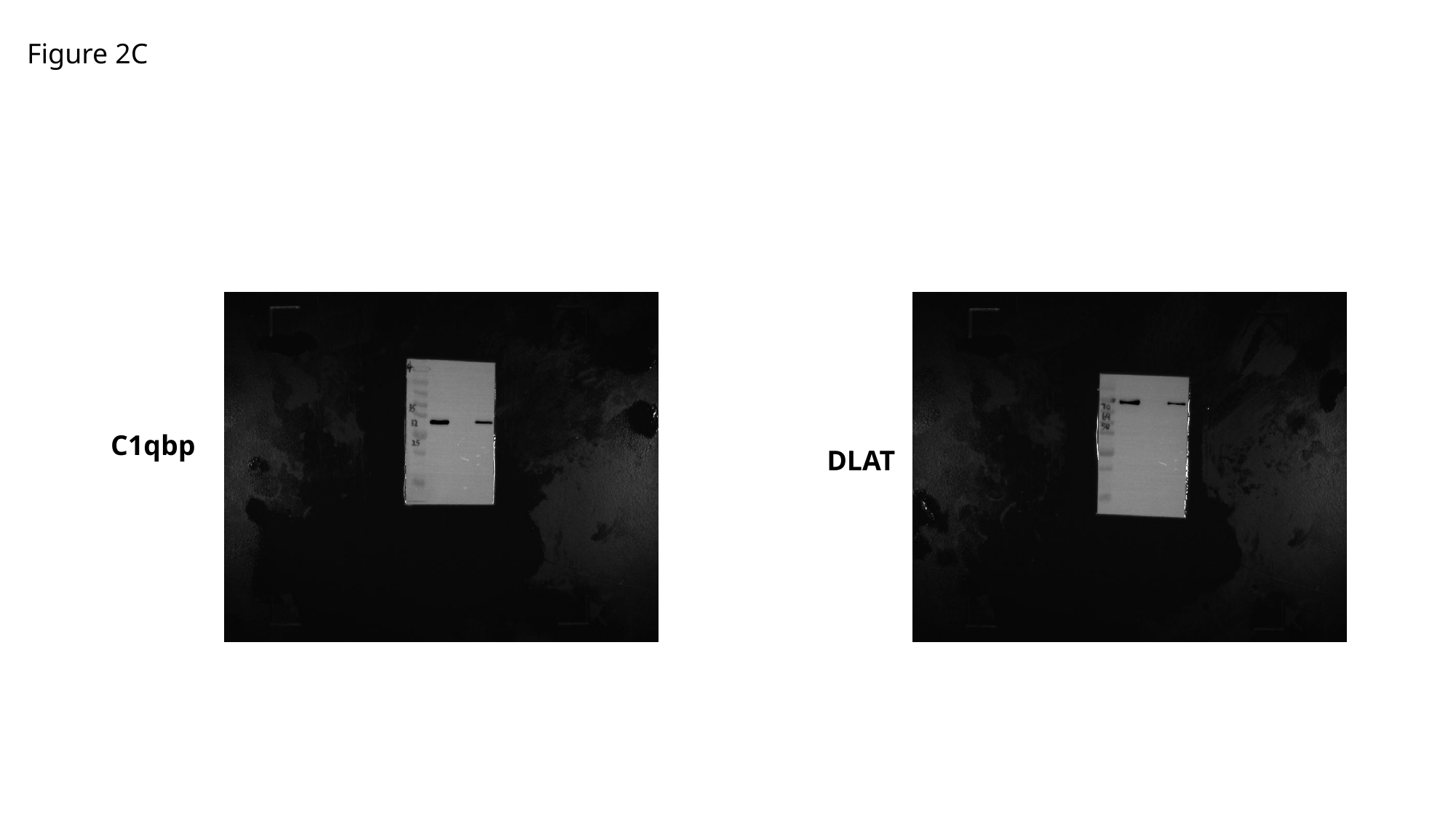

Figure 2C
C1qbp
DLAT

## Slide 9
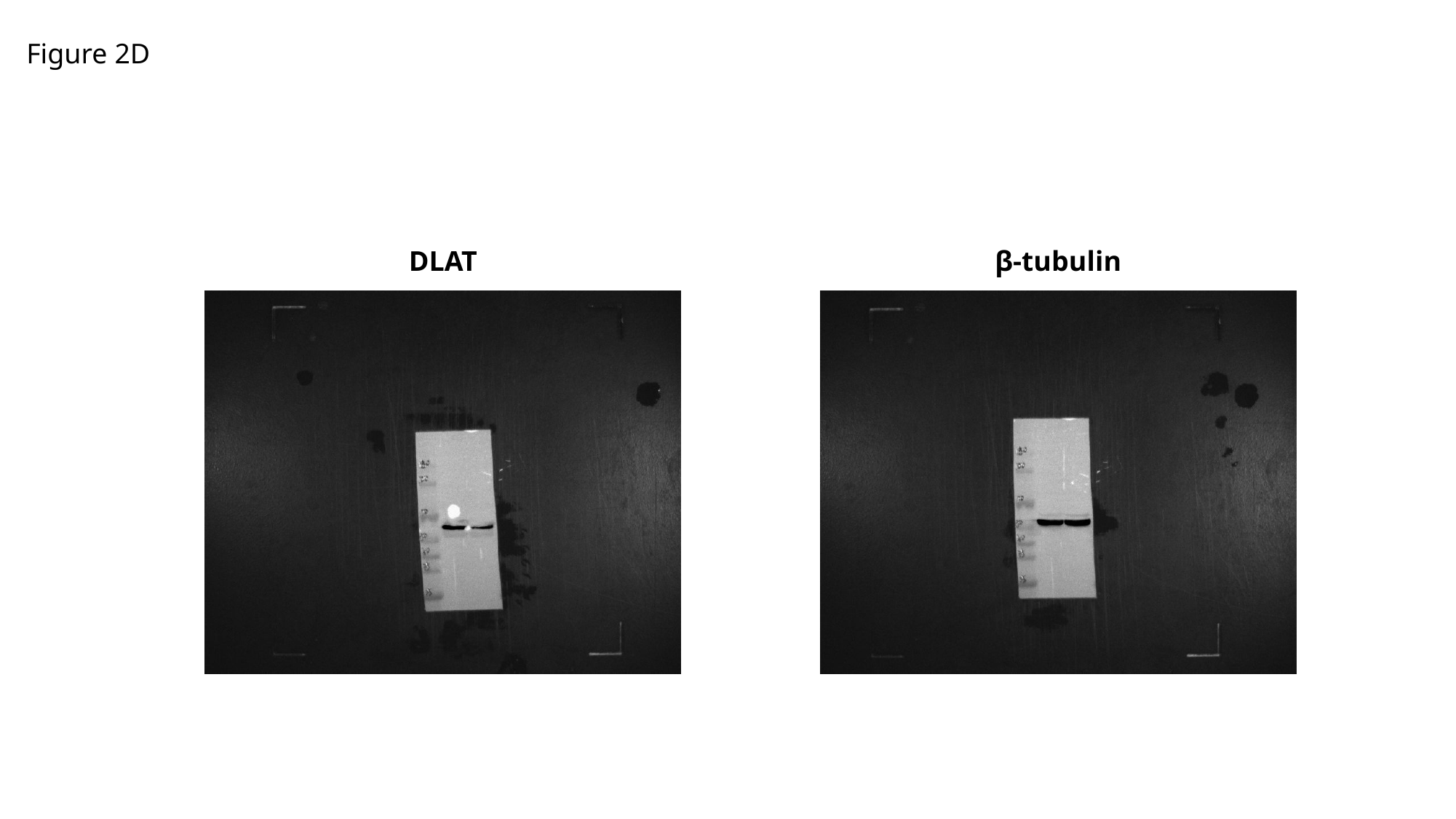

Figure 2D
DLAT
β-tubulin

## Slide 10
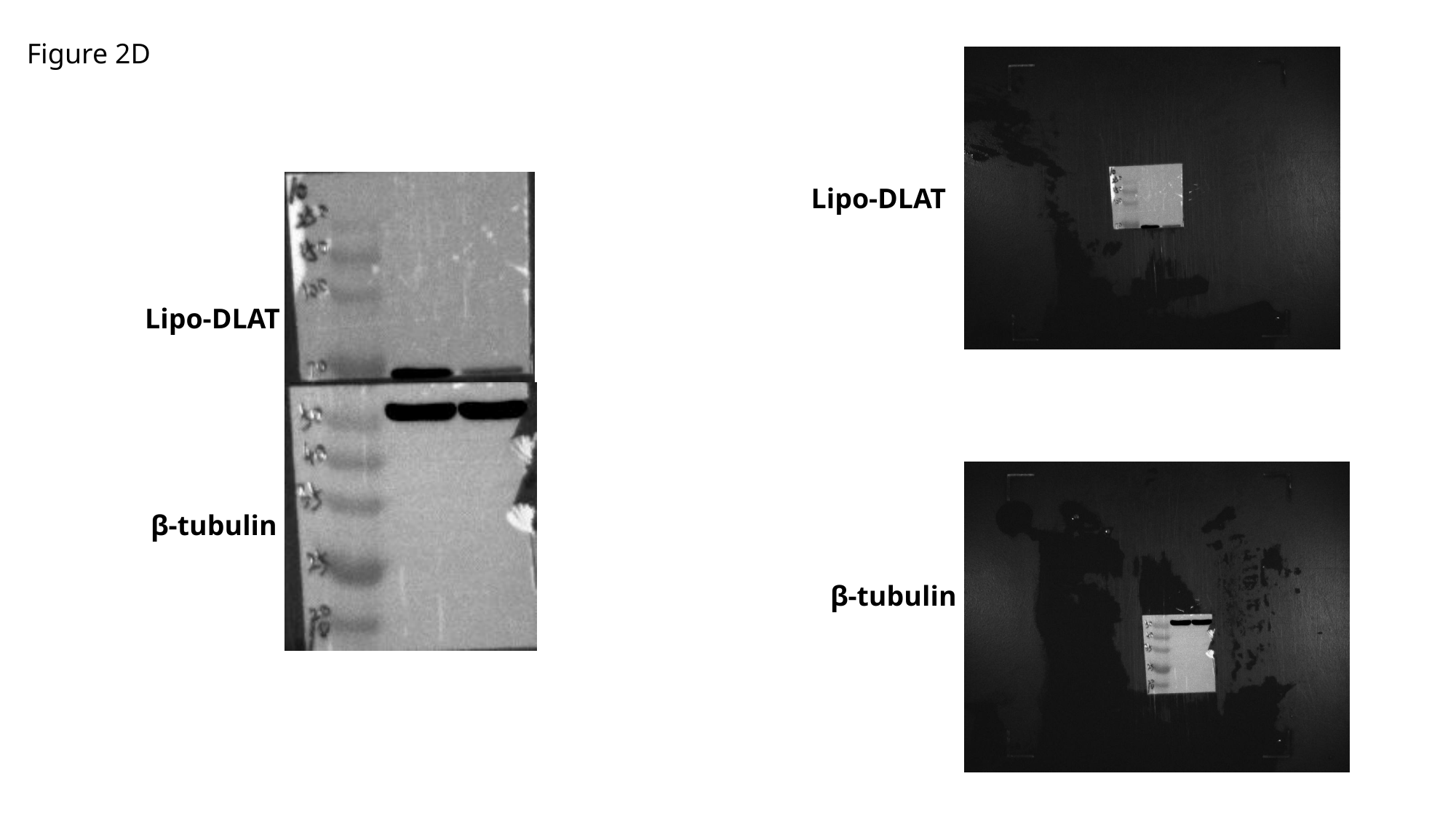

Figure 2D
Lipo-DLAT
Lipo-DLAT
β-tubulin
β-tubulin

## Slide 11
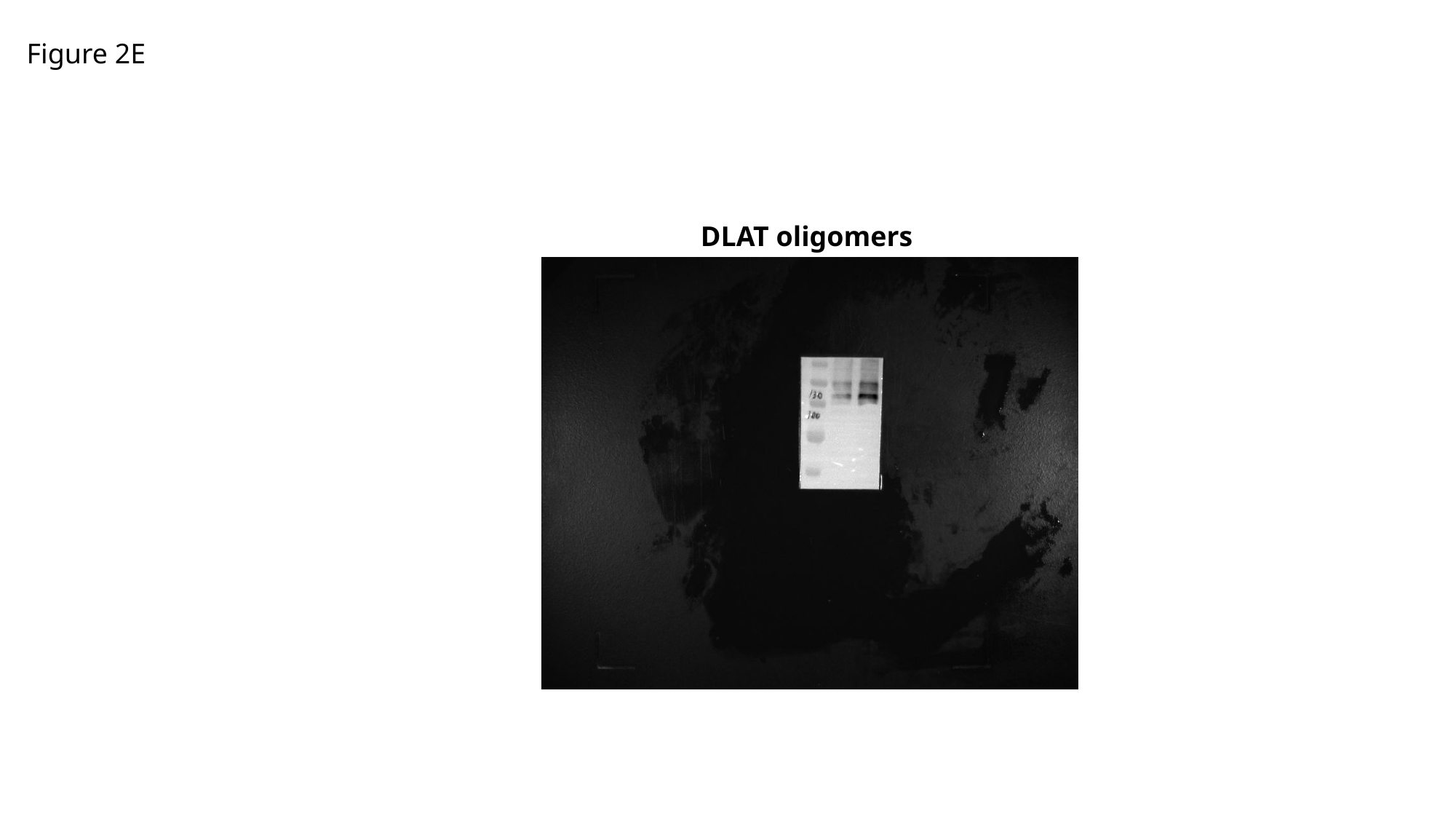

Figure 2E
DLAT oligomers

## Slide 12
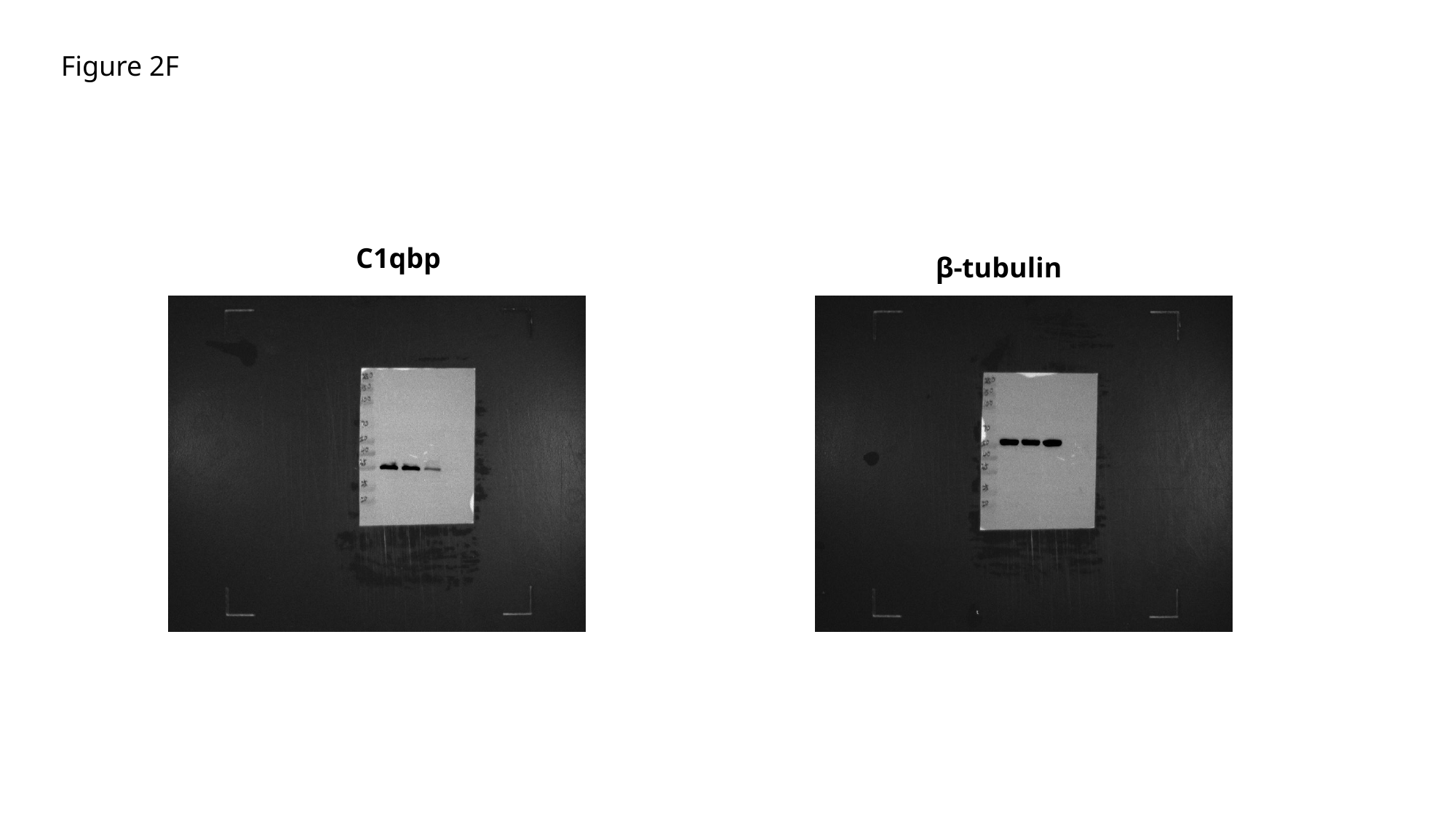

Figure 2F
C1qbp
β-tubulin

## Slide 13
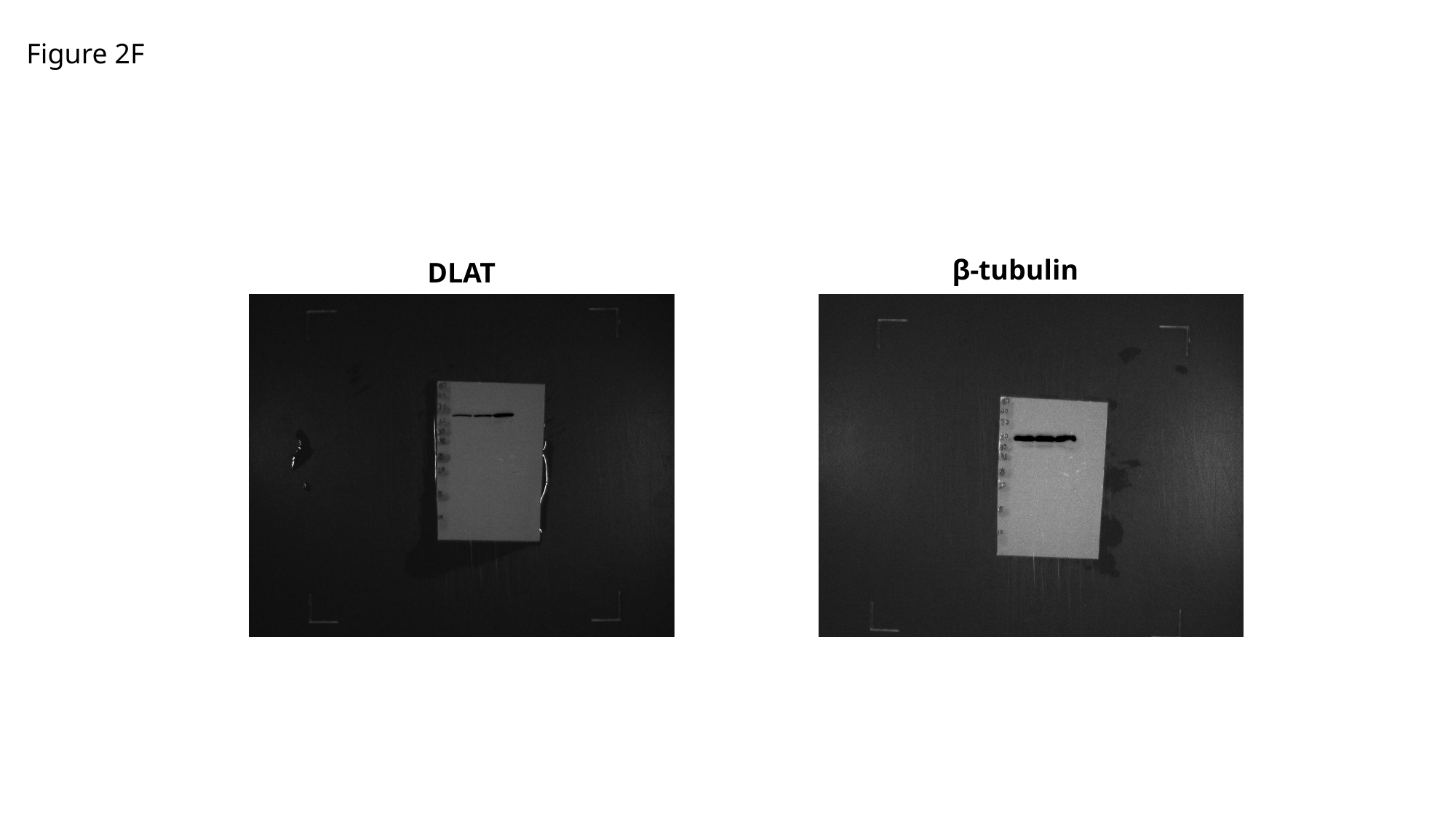

Figure 2F
β-tubulin
DLAT

## Slide 14
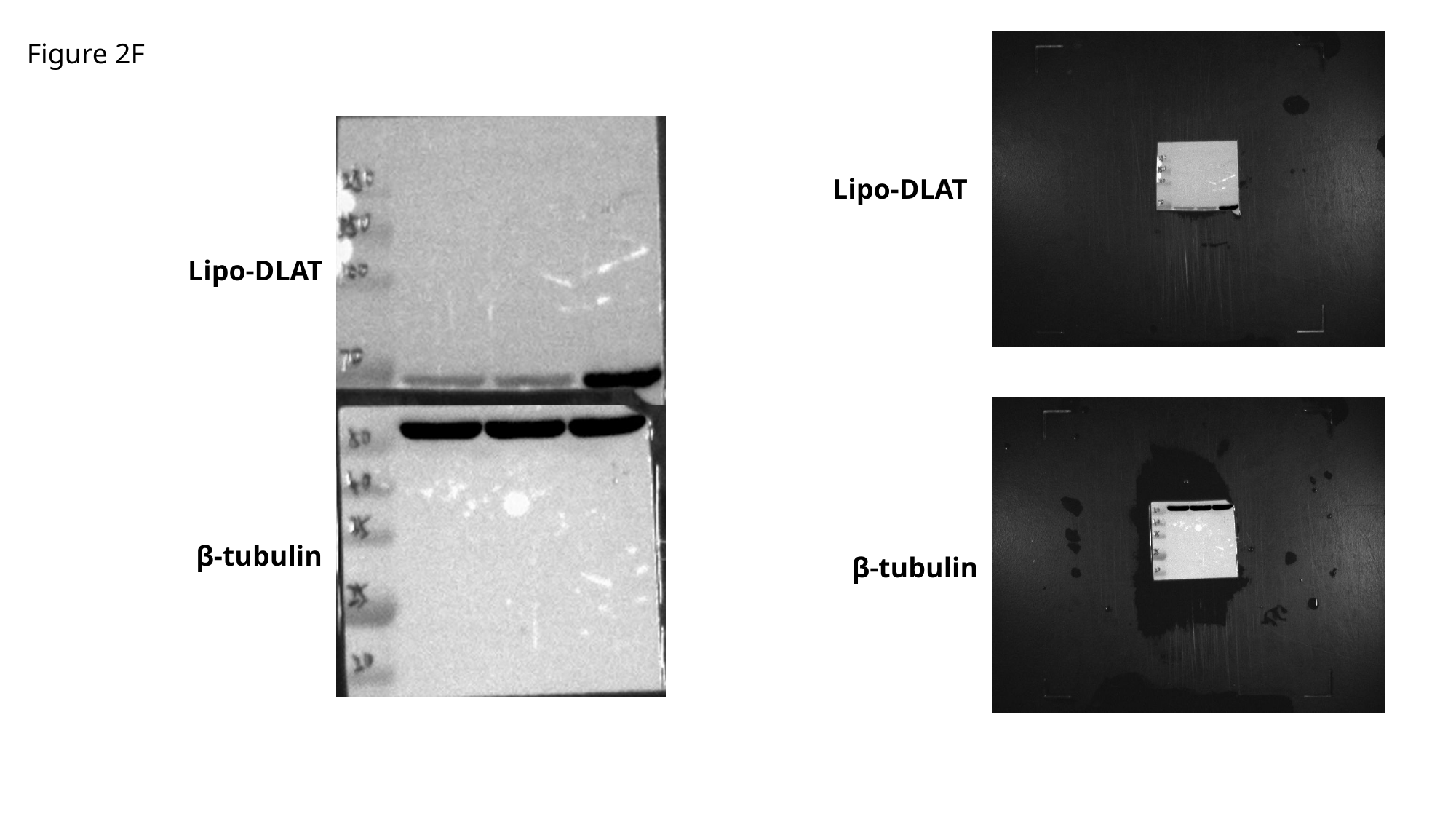

Figure 2F
Lipo-DLAT
Lipo-DLAT
β-tubulin
β-tubulin

## Slide 15
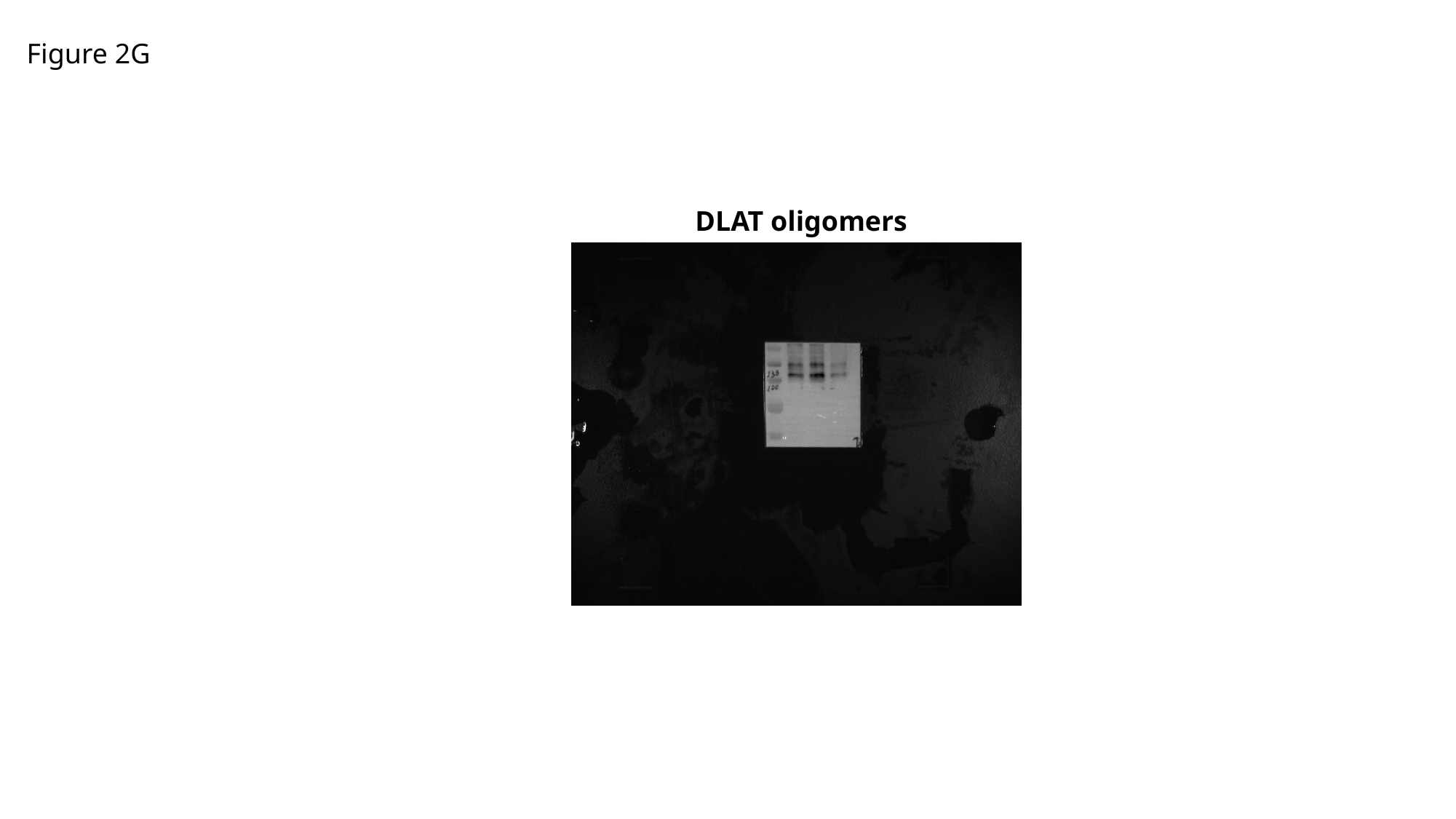

Figure 2G
DLAT oligomers

## Slide 16
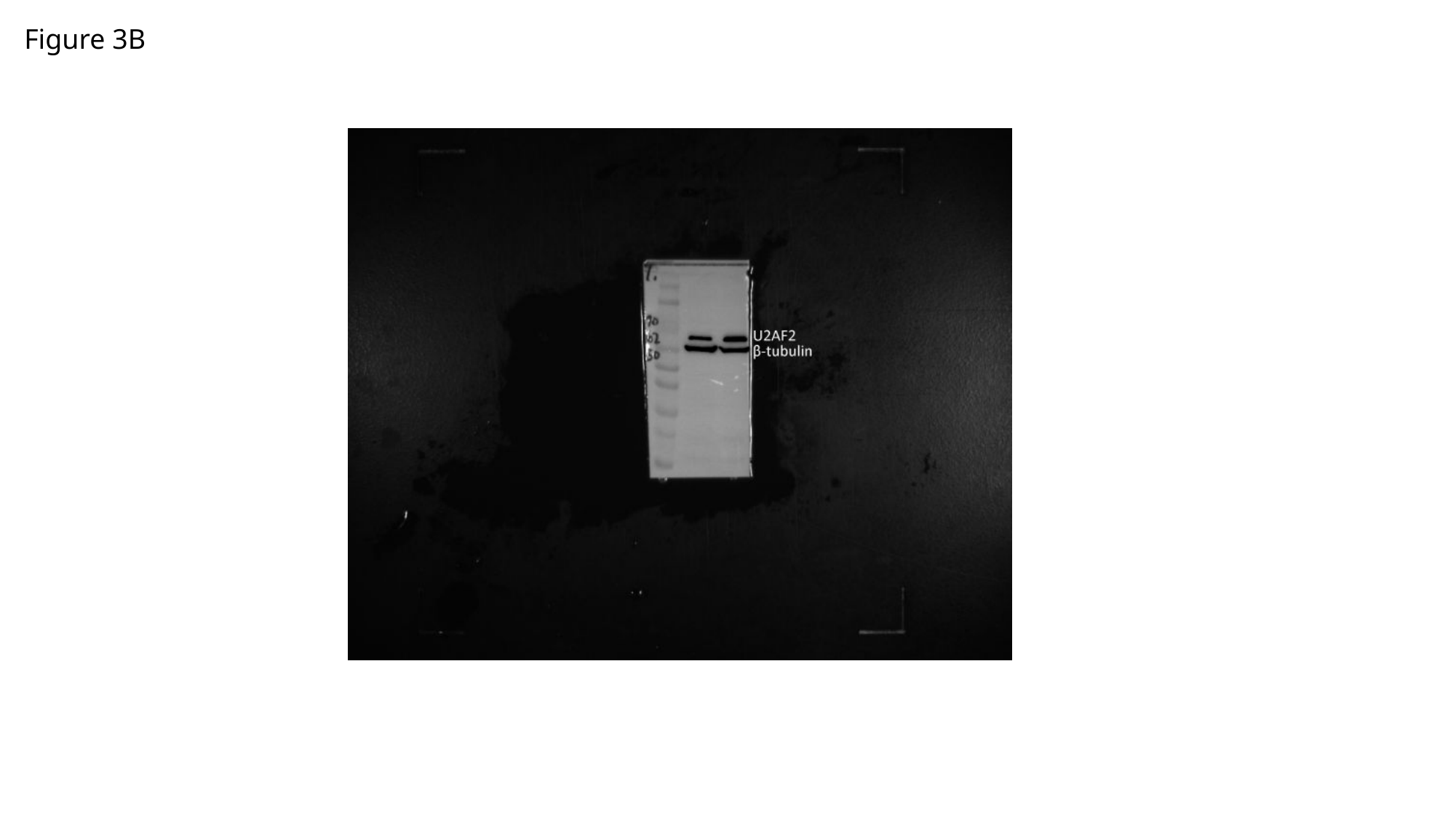

Figure 3B

## Slide 17
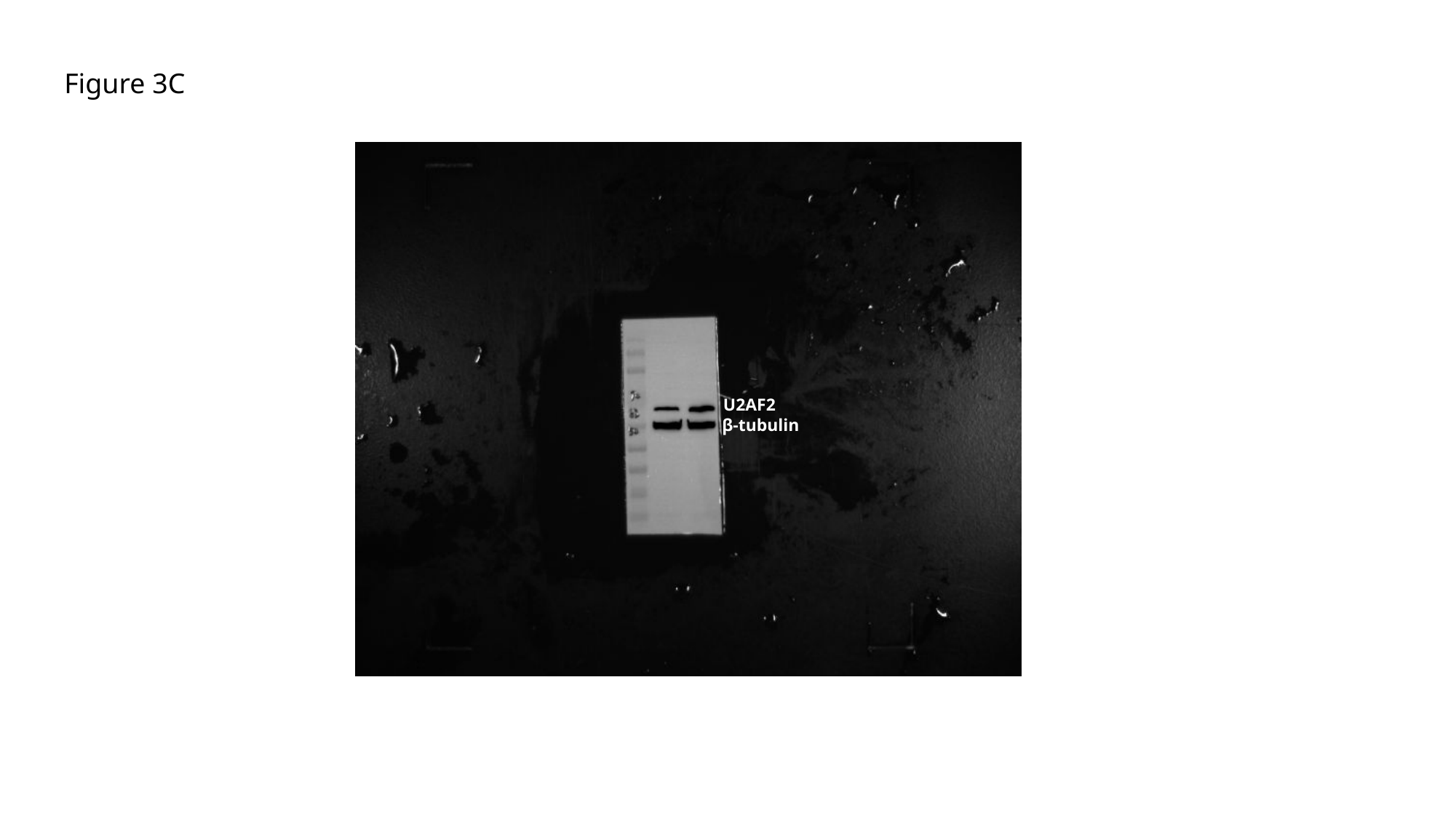

Figure 3C
U2AF2
β-tubulin

## Slide 18
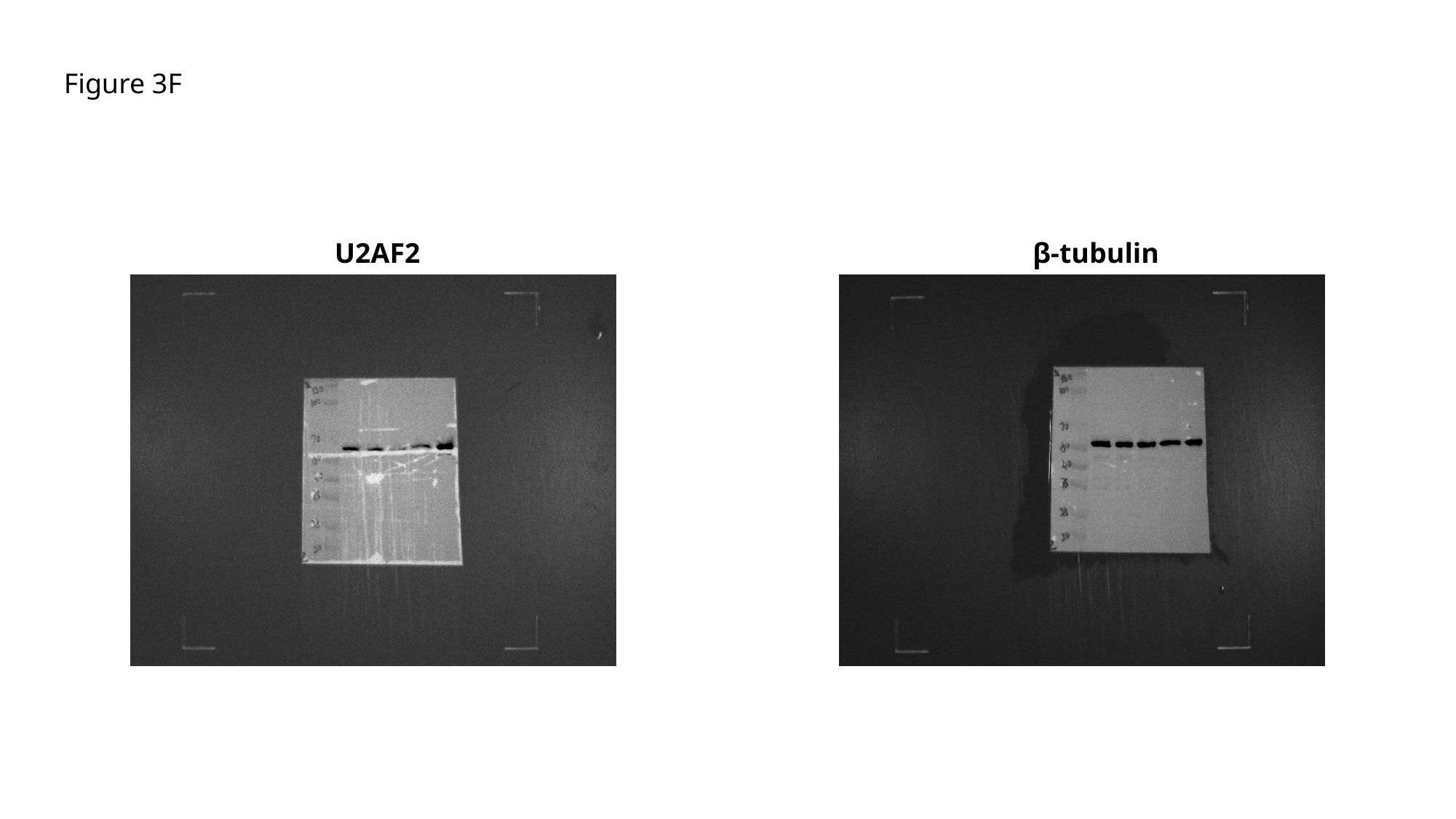

Figure 3F
U2AF2
β-tubulin

## Slide 19
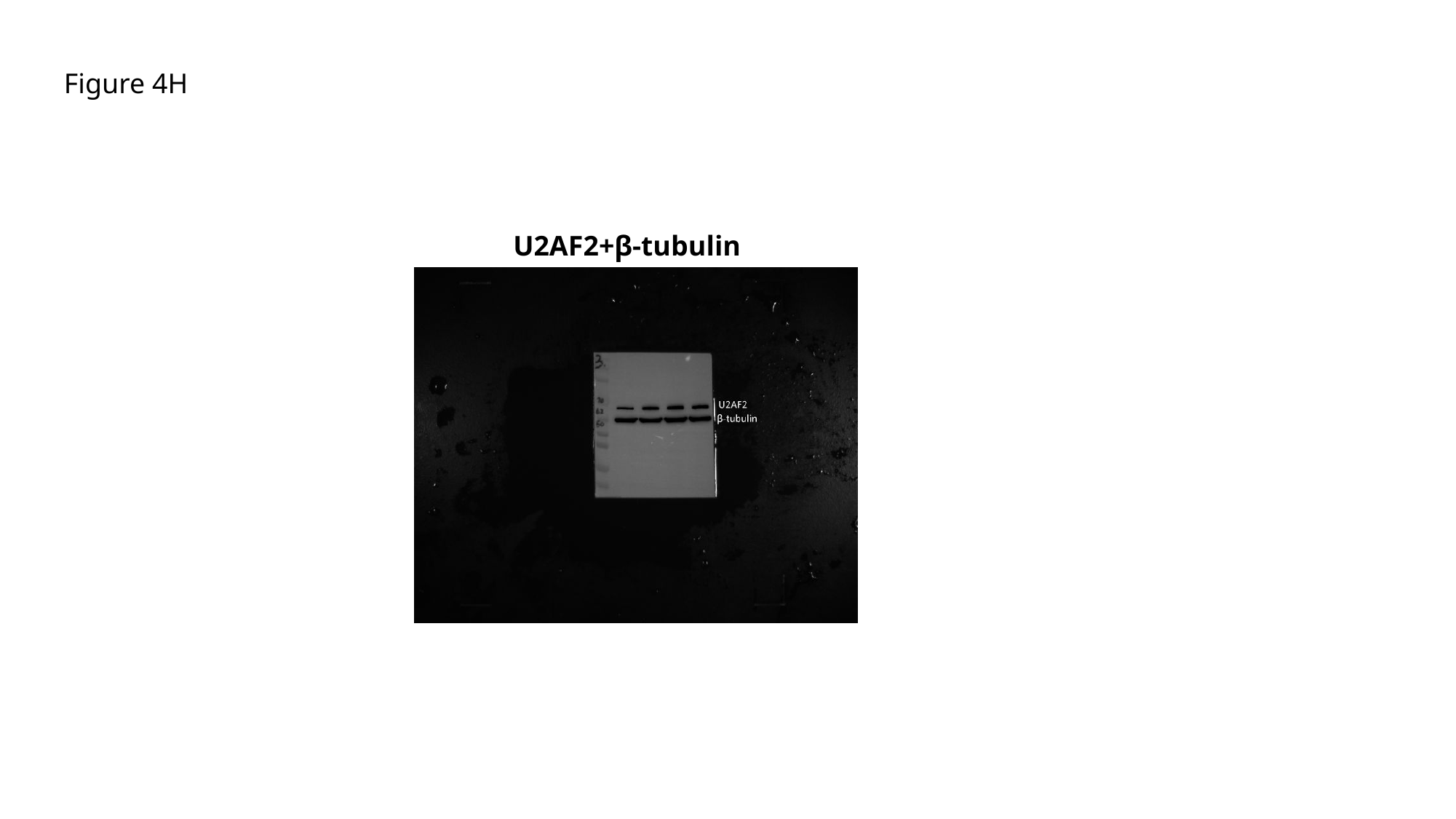

Figure 4H
U2AF2+β-tubulin

## Slide 20
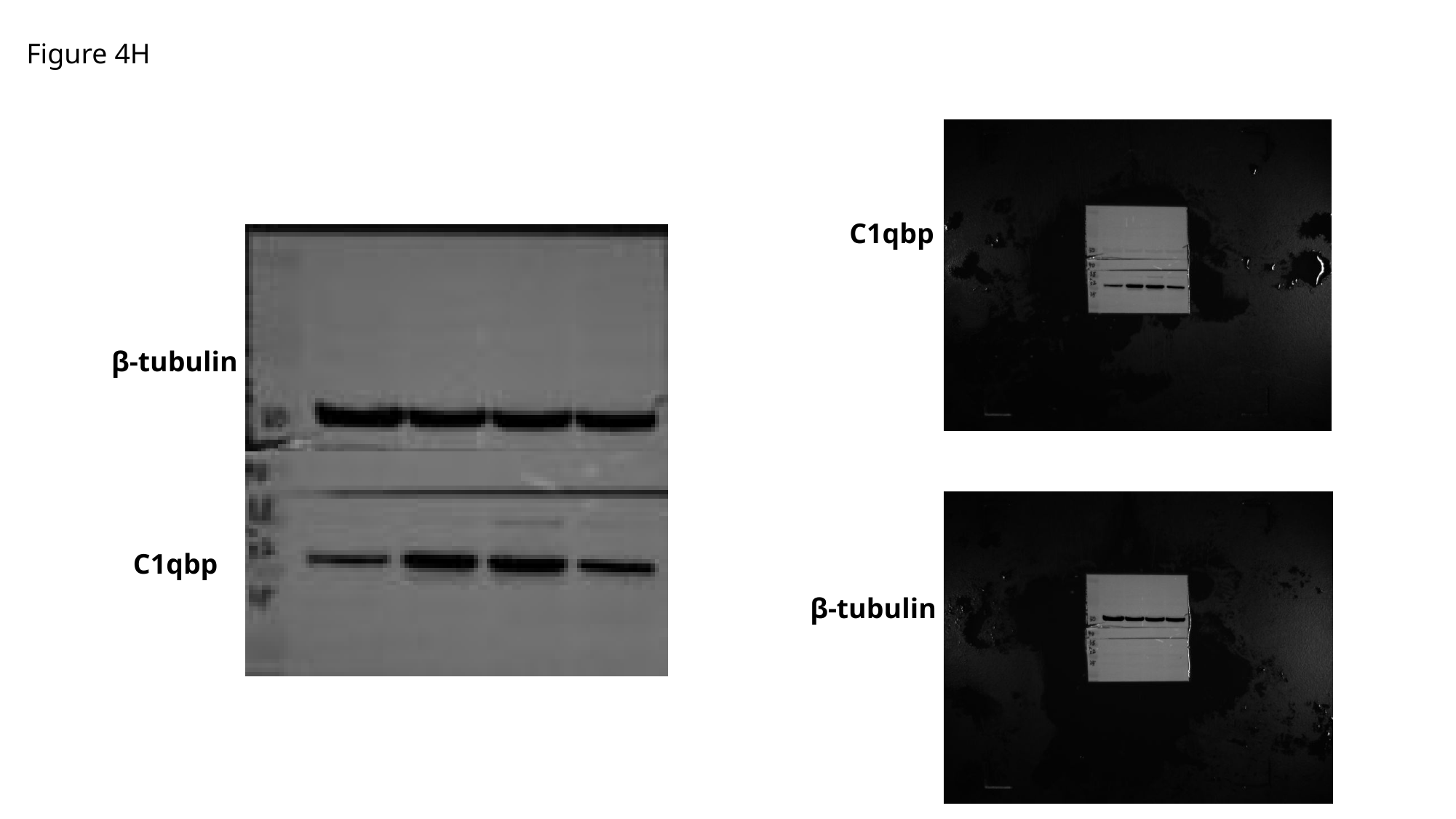

Figure 4H
C1qbp
β-tubulin
C1qbp
β-tubulin

## Slide 21
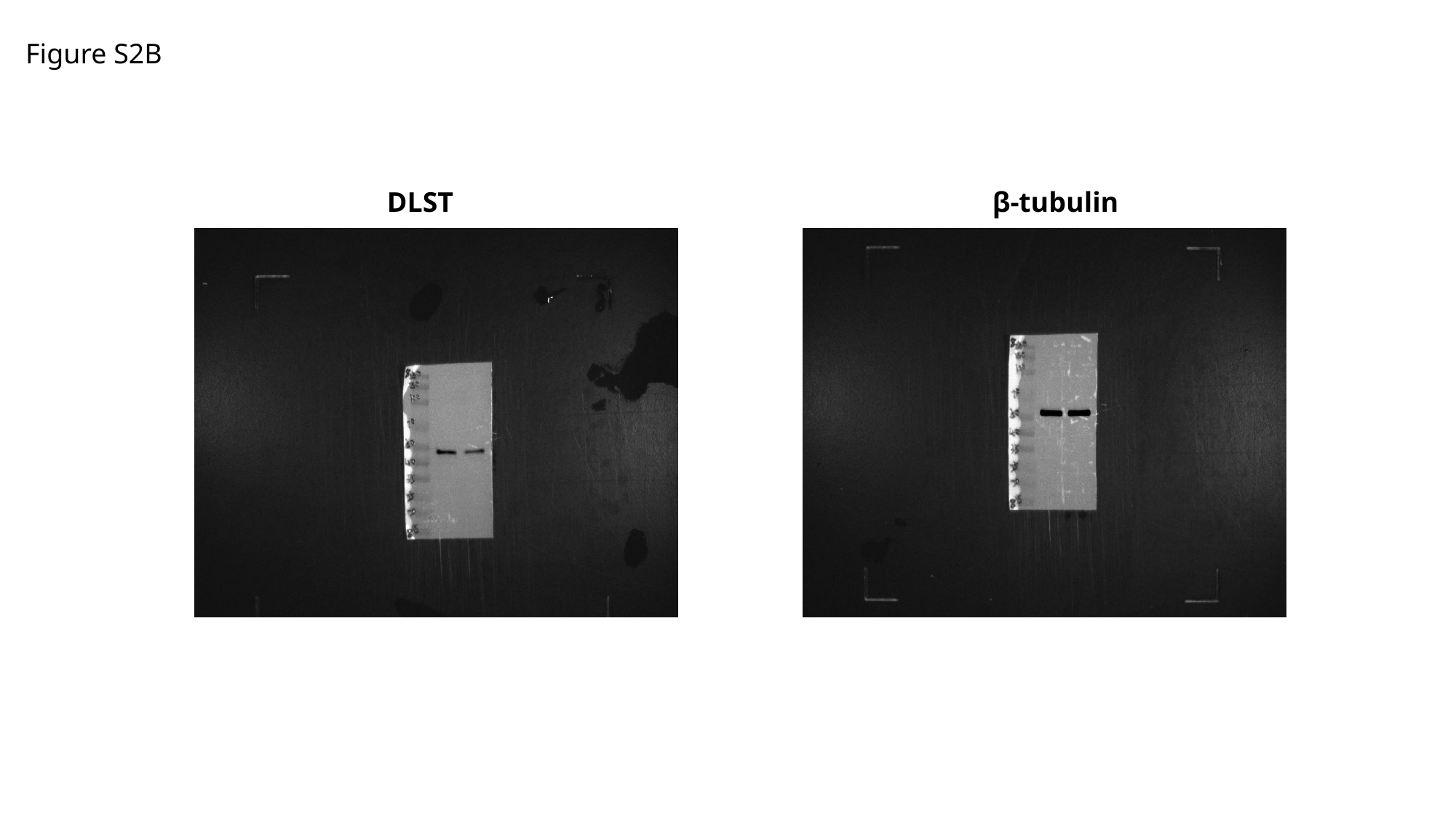

Figure S2B
DLST
β-tubulin

## Slide 22
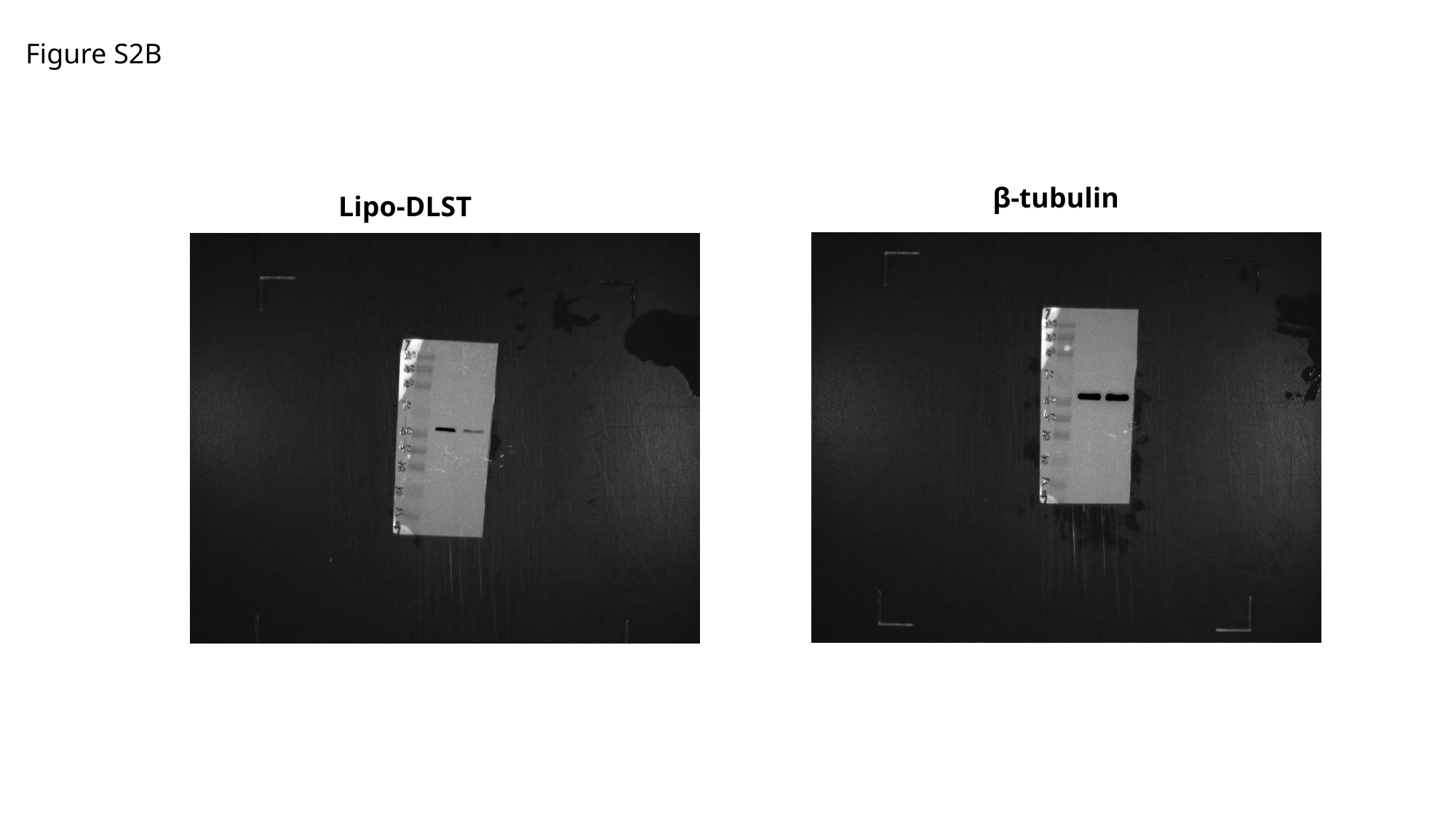

Figure S2B
β-tubulin
Lipo-DLST

## Slide 23
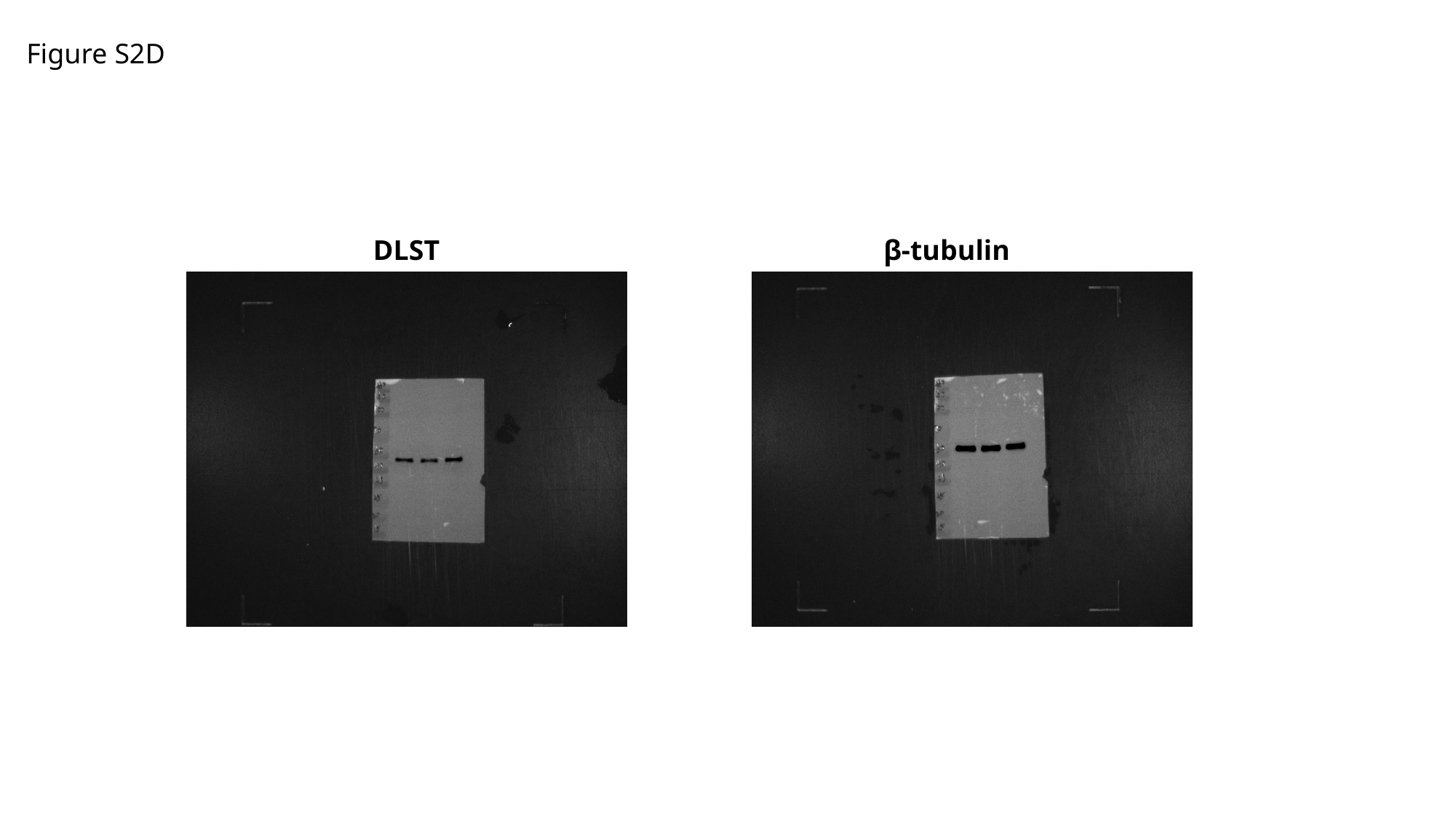

Figure S2D
DLST
β-tubulin

## Slide 24
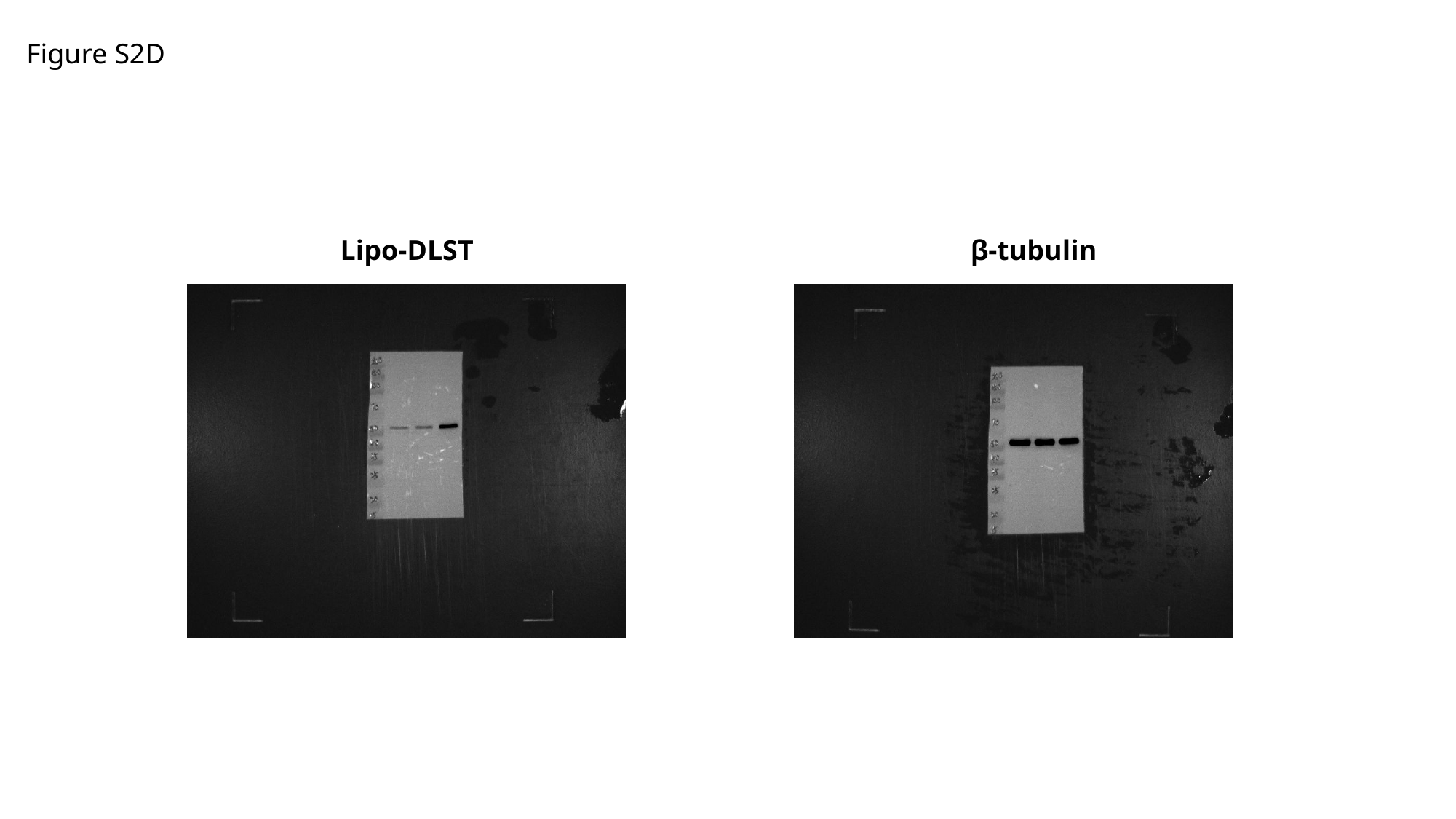

Figure S2D
Lipo-DLST
β-tubulin

## Slide 25
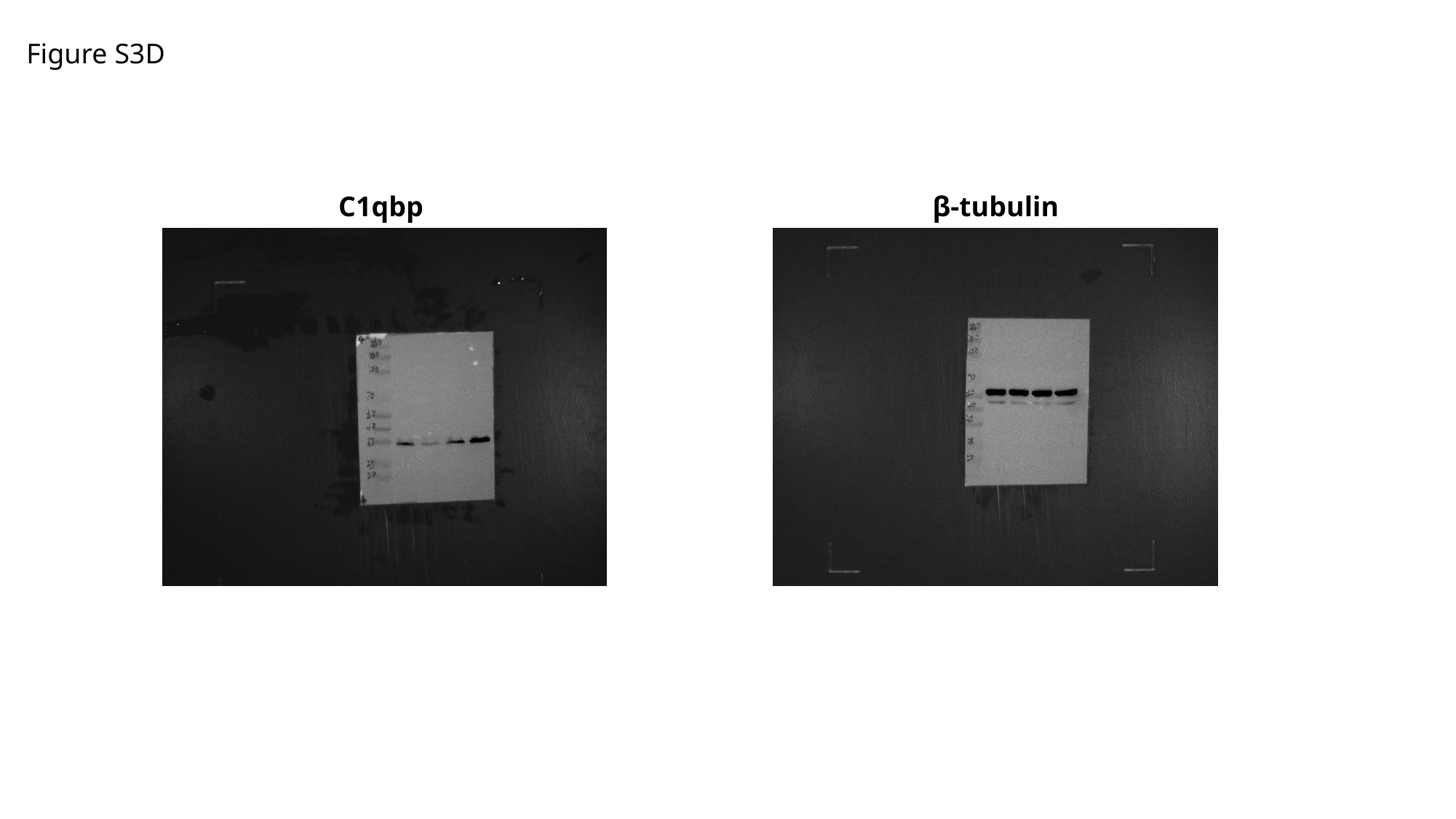

Figure S3D
C1qbp
β-tubulin

## Slide 26
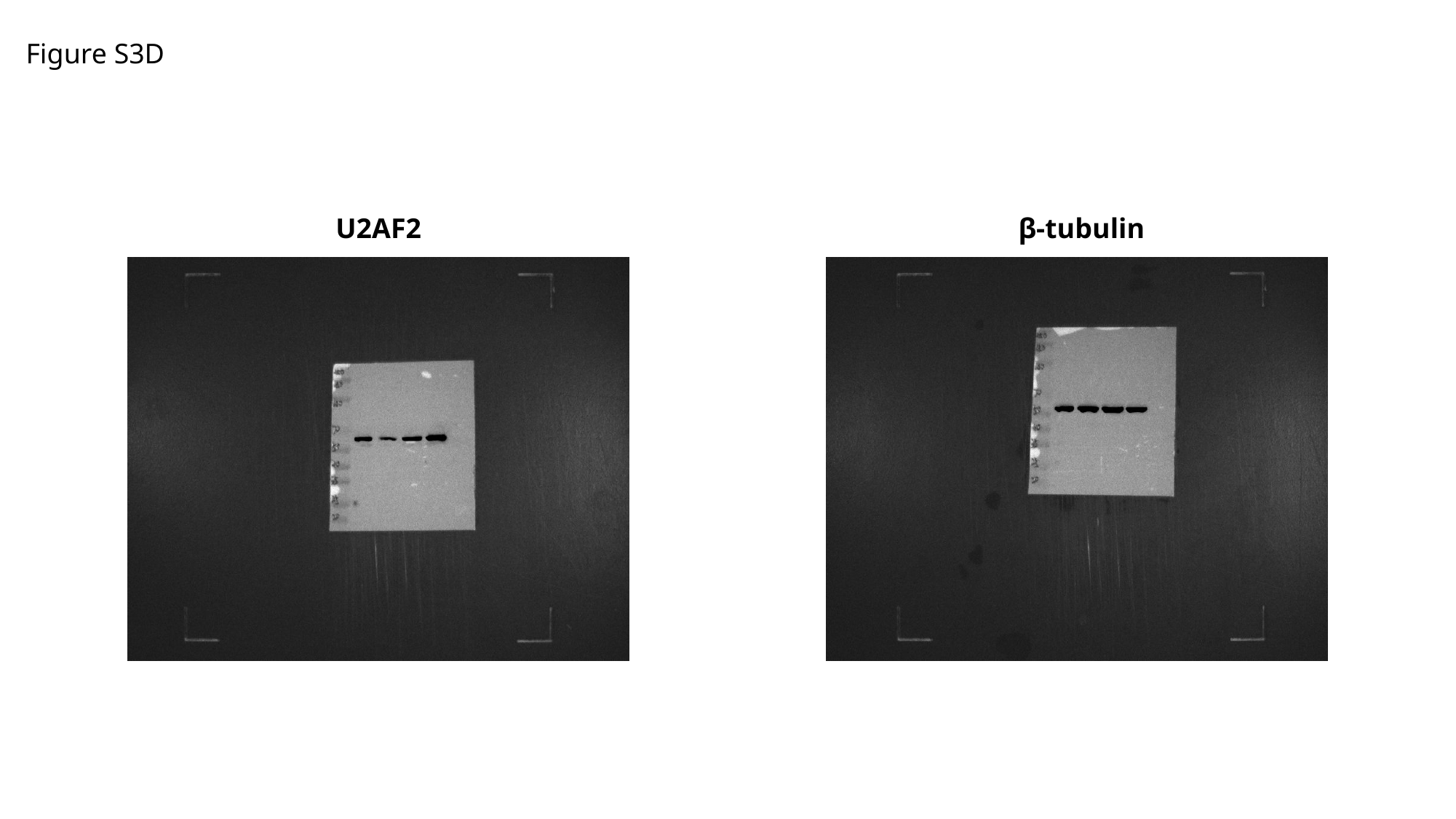

Figure S3D
U2AF2
β-tubulin

## Slide 27
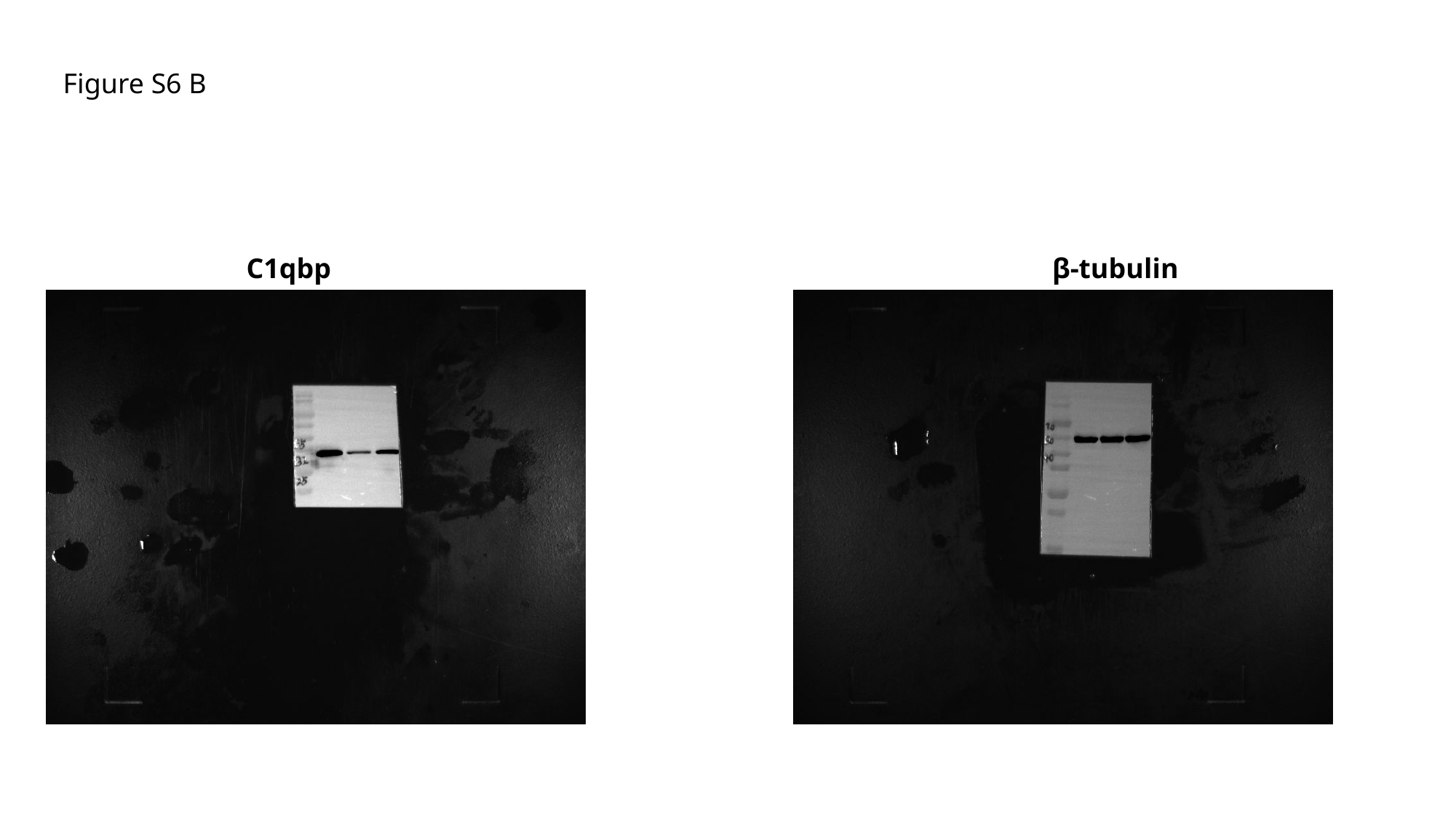

Figure S6 B
C1qbp
β-tubulin

## Slide 28
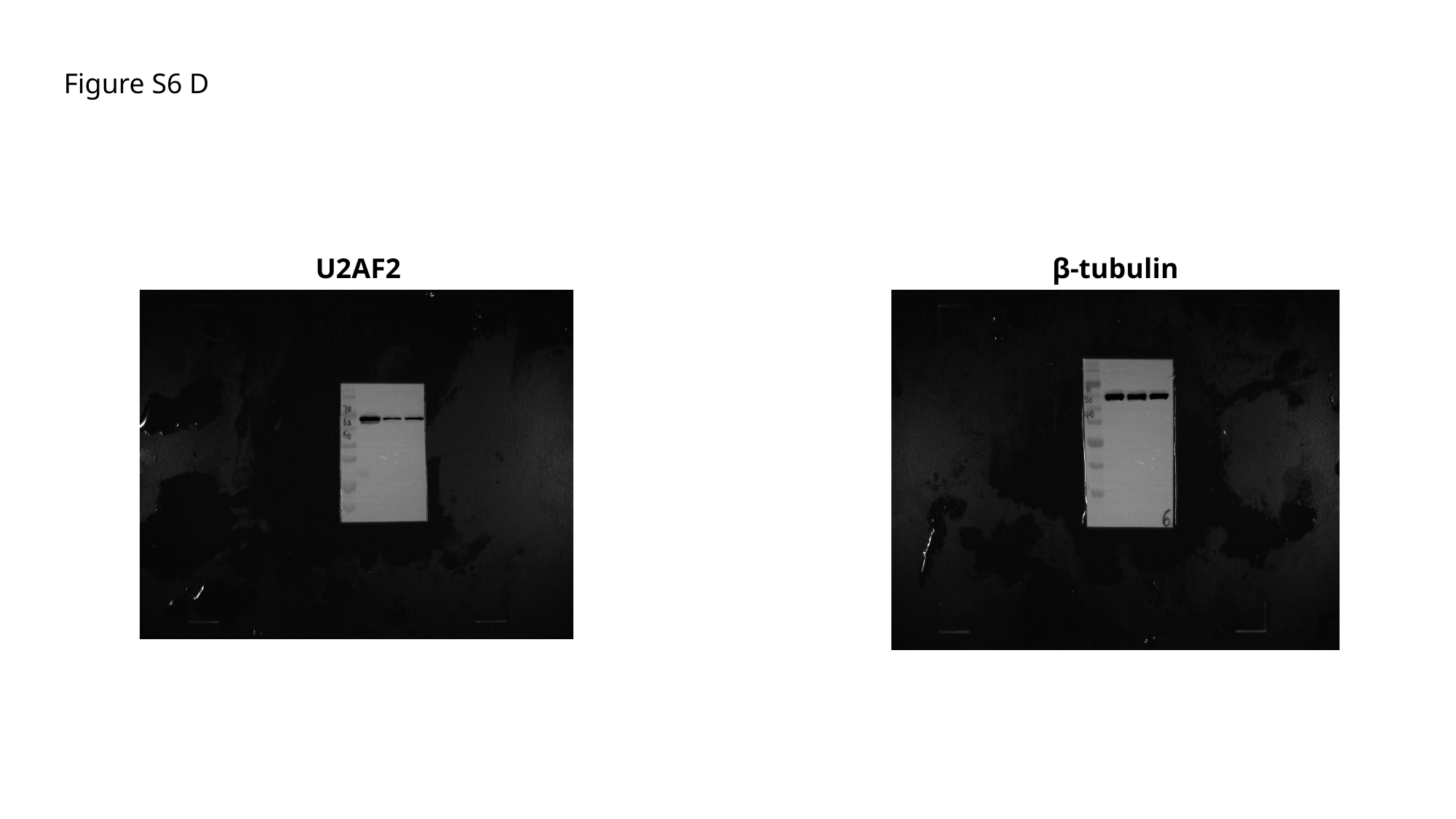

Figure S6 D
U2AF2
β-tubulin

## Slide 29
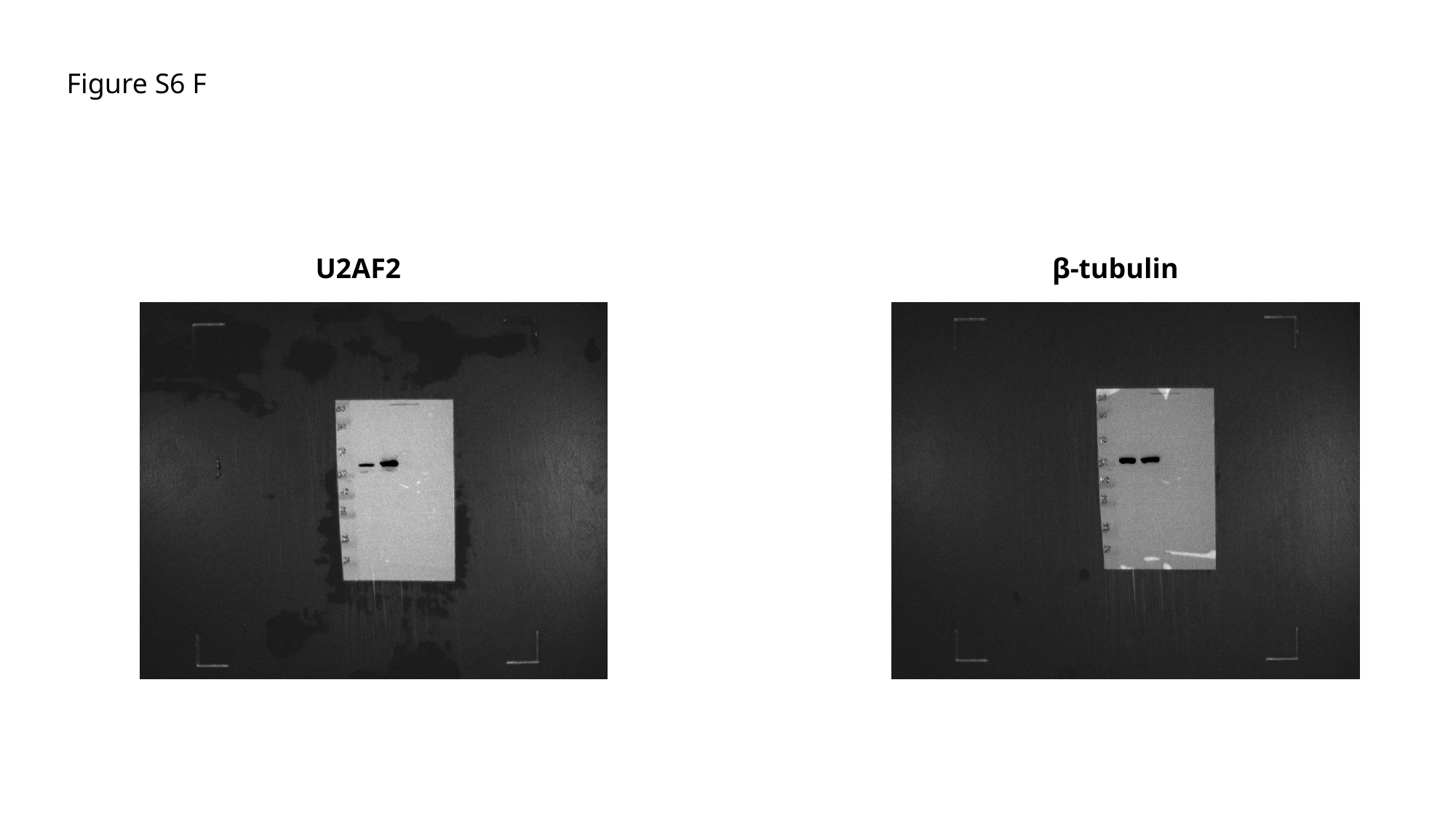

Figure S6 F
U2AF2
β-tubulin

## Slide 30
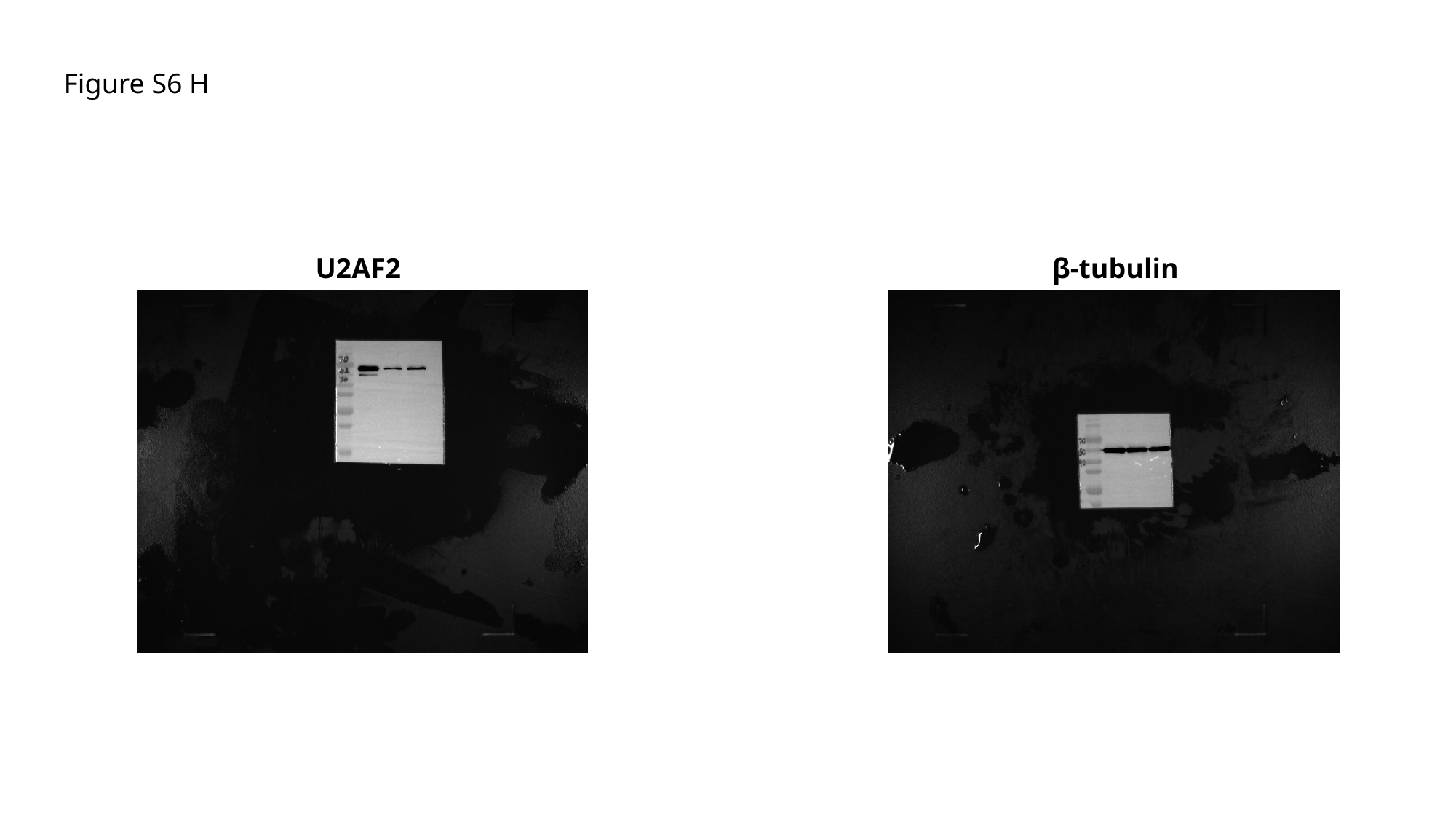

Figure S6 H
U2AF2
β-tubulin
