# Supplementary figures and images for: Vascular smooth muscle cell RNA-binding protein U2AF2 induces copper death by regulating C1qbp expression, delaying development of atherosclerosise
Source: Biol Res. 2026 Jan 28;59:13. doi: 10.1186/s40659-026-00672-3 (PMC12924209; doi:10.1186/s40659-026-00672-3)

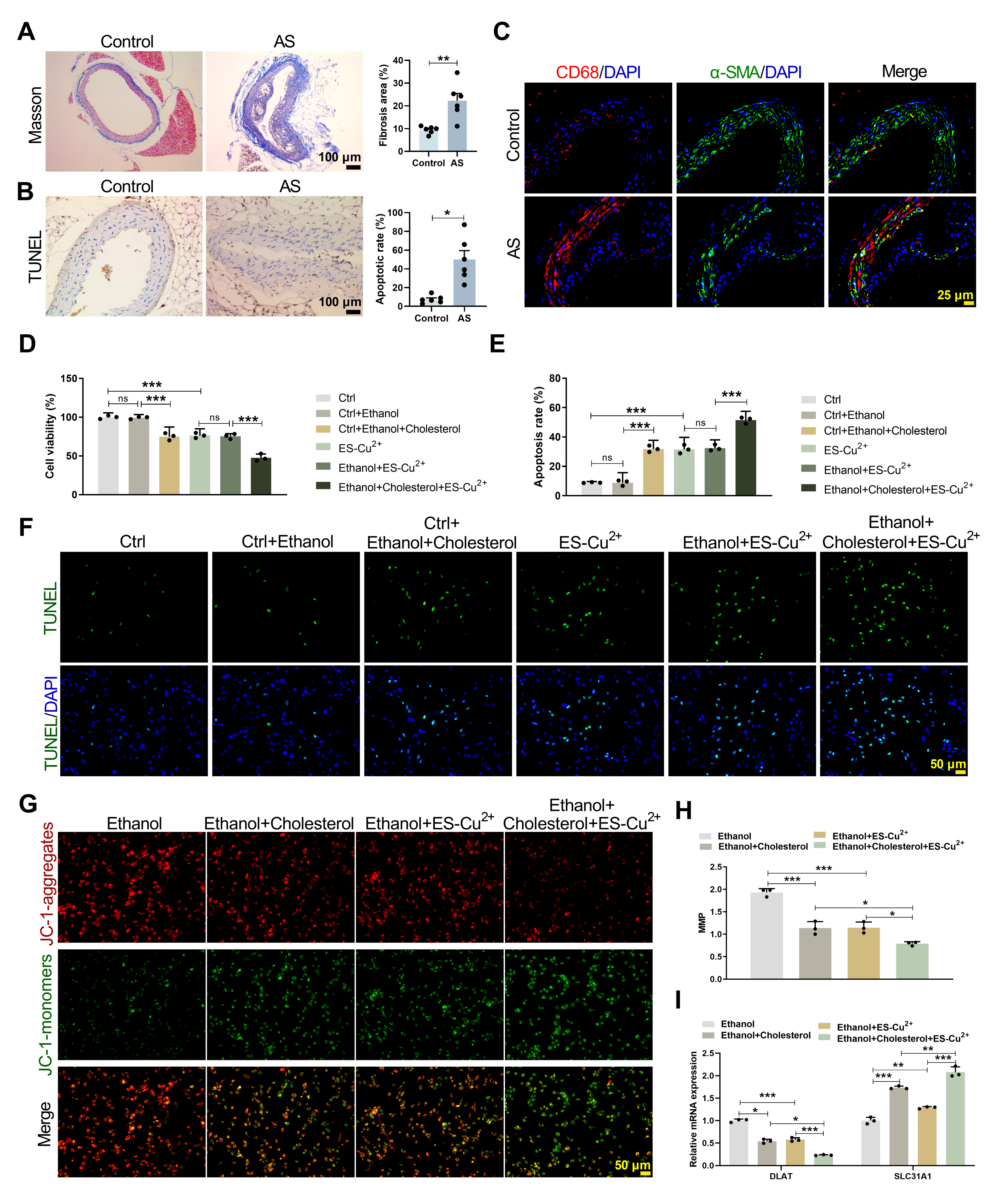

Supplement: Supplementary file 2 — Supplementary Material 2: Fig. S1. Increased Cuproptosis in the AS model. ApoE−/− male mice were fed an HFD for 12 weeks and the aorta was collected for subsequent experiments. (A) Masson’s trichrome staining was used to measure collagen content. MeanControl=9.453; MeanAS=22.27; 95%CI[5.498, 20.13]. (B) TUNEL staining was performed to show dead cells in the fibrous cap of the plaque surface. ApoE−/− male mice were fed an HFD for 12 weeks and treated with Ethanol + Cholesterol + ES-Cu2+. MeanControl=7.353; MeanAS=49.87; 95%CI[20.86, 64.18]. (C) Multiplex immunofluorescence for detecting CD68 and α-SMA in the abdominal aorta. Verification experiment demonstrating that the addition of ethanol has no effect on cells. (D) CCK8 assay was used to detect proliferation. (E-F) The TUNEL assay was used to detect apoptosis. HAVSMCs were selected and treated with the copper ionophore ES-Cu2+. (G-H) JC-1 staining was used to analyze mitochondrial membrane potential. (I) qRT-PCR was performed to detect the expression of DLAT and SLC31A1. *p < 0.05, **p < 0.01, ***p < 0.001 versus Control/Ctrl/Ctrl + Ethanol/Ethanol + ES-Cu2+/Ethanol/Ethanol + Cholesterol. ns means no significant difference versus Ctrl/ES-Cu2+ [file 40659_2026_672_MOESM2_ESM.tif]

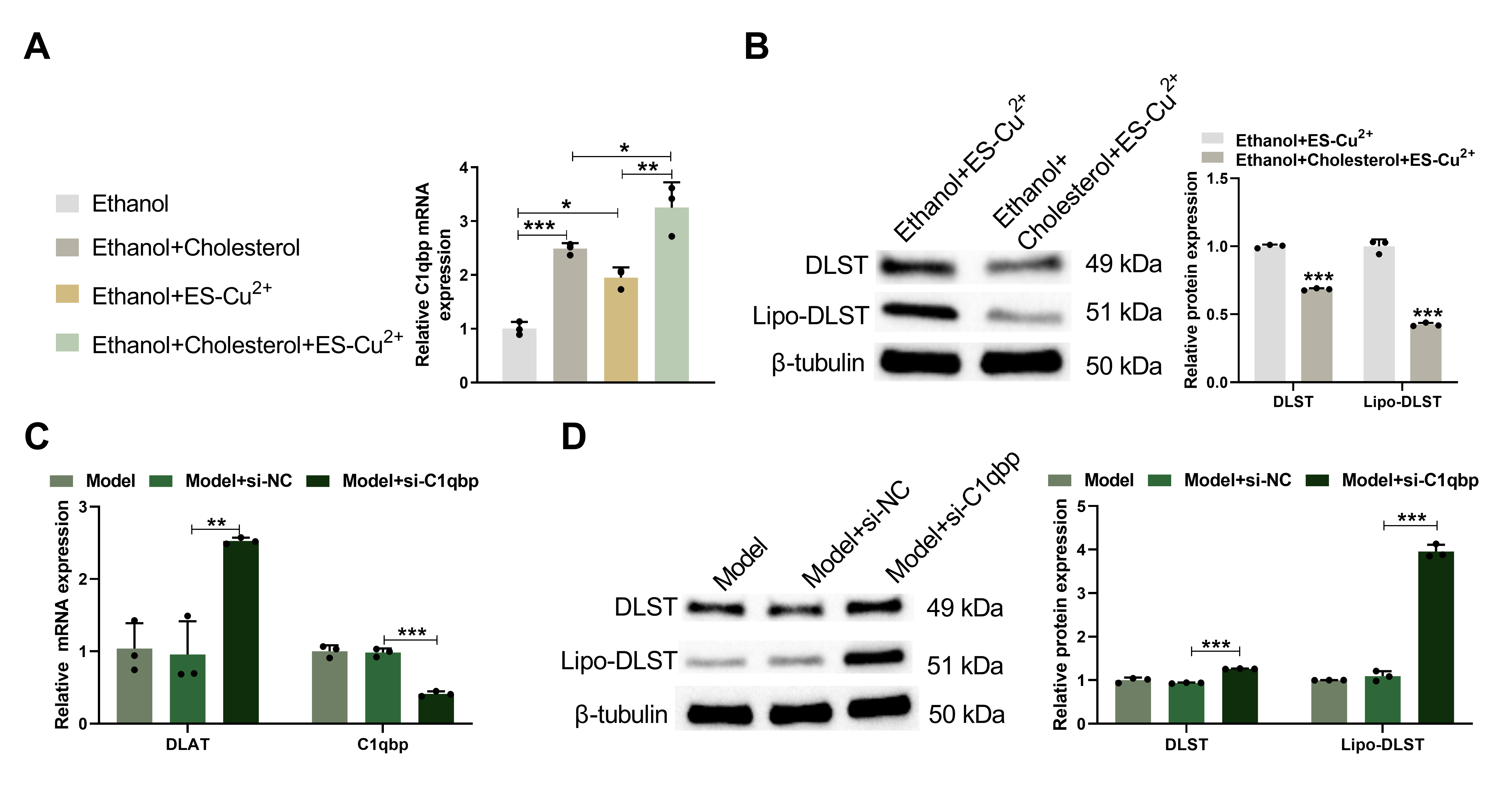

Supplement: Supplementary file 3 — Supplementary Material 3: Fig. S2. Knocking down C1qbp affects DLAT expression. HAVSMC cells were treated with ethanol, ethanol + cholesterol, ethanol + ES-Cu2+, and ethanol + cholesterol + ES-Cu2+, respectively. (A) qRT-PCR was used to detect the expression level of C1qbp mRNA. (B) WB was performed to detect DLST and Lipo-DLST protein expression. HAVSMC cells were co-treated with cholesterol and ES-Cu2+, followed by transfection with si-C1qbp. (C) qRT-PCR was performed to detect the expression levels of DLAT and C1qbp mRNA. (D) WB was used to detect DLST and Lipo-DLST protein expression. *p < 0.05, **p < 0.01, ***p < 0.001 versus Ethanol/Ethanol + Cholesterol/Ethanol + ES-Cu2+/Model + si-NC. NC means negative control of si-C1qbp [file 40659_2026_672_MOESM3_ESM.tif]

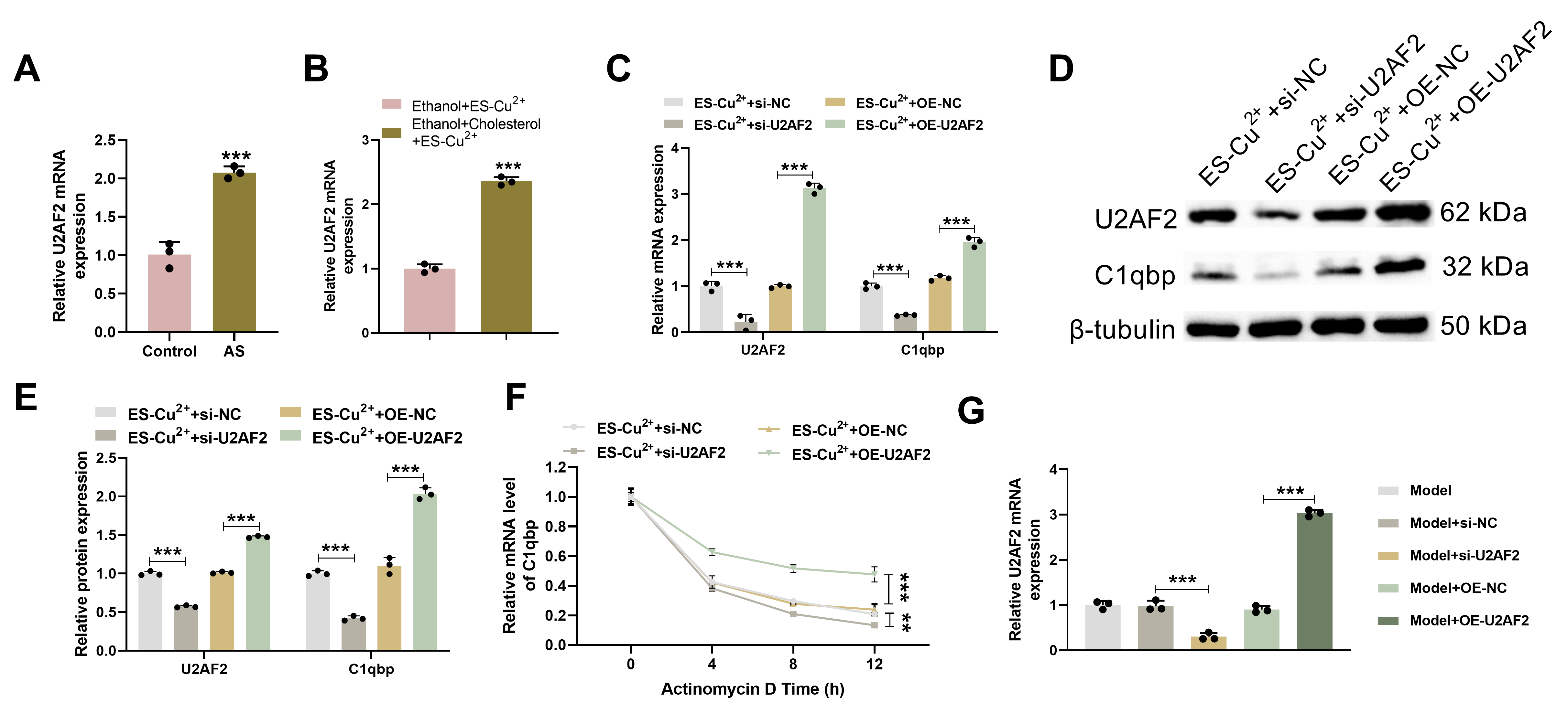

Supplement: Supplementary file 4 — Supplementary Material 4: Fig. S3. U2AF2 was increased in atherosclerosis. ApoE−/− male mice were fed an HFD for 12 weeks. A qRT-PCR was used to detect the expression of U2AF2 in the aortic tissues of the mice. MeanControl = 1.009; MeanAS = 2.076; 95% CI [0.7757, 1.358]. The HAVSMC cell line was then selected and subsequently treated with Ethanol + Cholesterol + ES-Cu2+. B qRT-PCR was used to detect the expression of U2AF2 in HAVSMC cells. HAVSMC cells were treated with ES-Cu2+, followed by si-NC, si-U2AF2, OE-NC and OE-U2AF2 treatments. C qRT-PCR detected the expression of U2AF2 and C1qbp in HAVSMC cells. D, E WB detection of U2AF2 and C1qbp protein expression. F Actinomycin D detection of C1qbp mRNA stability. HAVSMC cells were co-treated with cholesterol and ES-Cu2+, followed by si-U2AF2 and OE-U2AF2 treatments. G qRT-PCR was used to detect the mRNA expression of U2AF2 in HAVSMC cells. *p < 0.05, **p < 0.01, ***p < 0.001 versus Control/Ethanol + ES-Cu2+/ES-Cu2++si-NC/ES-Cu2++OE-NC/Model + si-NC/Model + OE-NC. NC means negative control of si-U2AF2/OE-U2AF2 [file 40659_2026_672_MOESM4_ESM.tif]

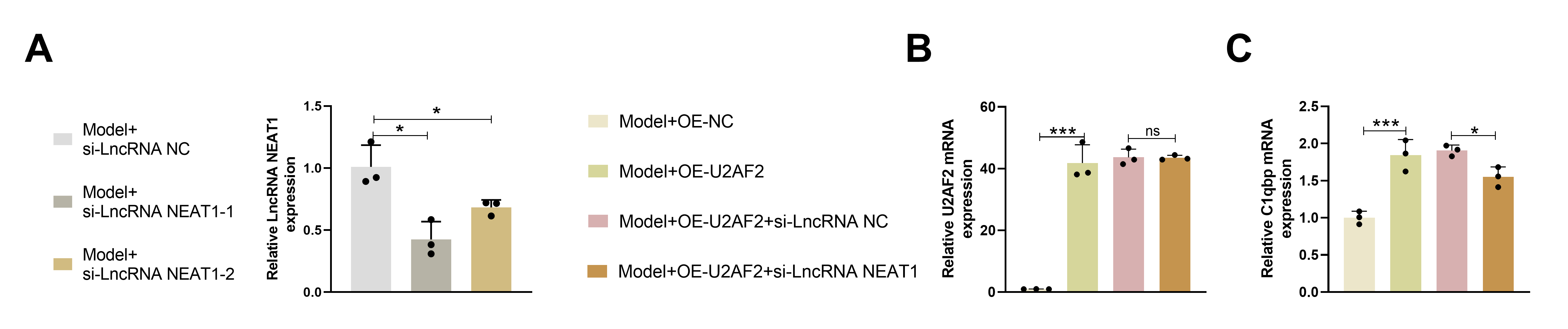

Supplement: Supplementary file 5 — Supplementary Material 5: Fig. S4. U2AF2 recruits to C1qbp mRNA via LncRNA NEAT1 to regulate mRNA stability. The HAVSMC cell line was selected and subsequently treated with si-lncRNA NEAT1. A qRT-PCR was used to detect the knockdown efficiency of lncRNA NEAT1 in HAVSMC cells. The HAVSMC cell line was then selected and subsequently treated with OE-U2AF2 and OE-U2AF2 + si-lncRNA NEAT1. B, C qRT-PCR was used to detect the expression of U2AF2 and C1qbp in HAVSMC cells. *p < 0.05, **p < 0.01, ***p < 0.001 versus Model + si-lncRNA NC/Model + OE-NC/Model + OE-U2AF2 + si-lncRNA NC. NC means negative control of OE-U2AF2/si-lncRNA NEAT1 [file 40659_2026_672_MOESM5_ESM.tif]

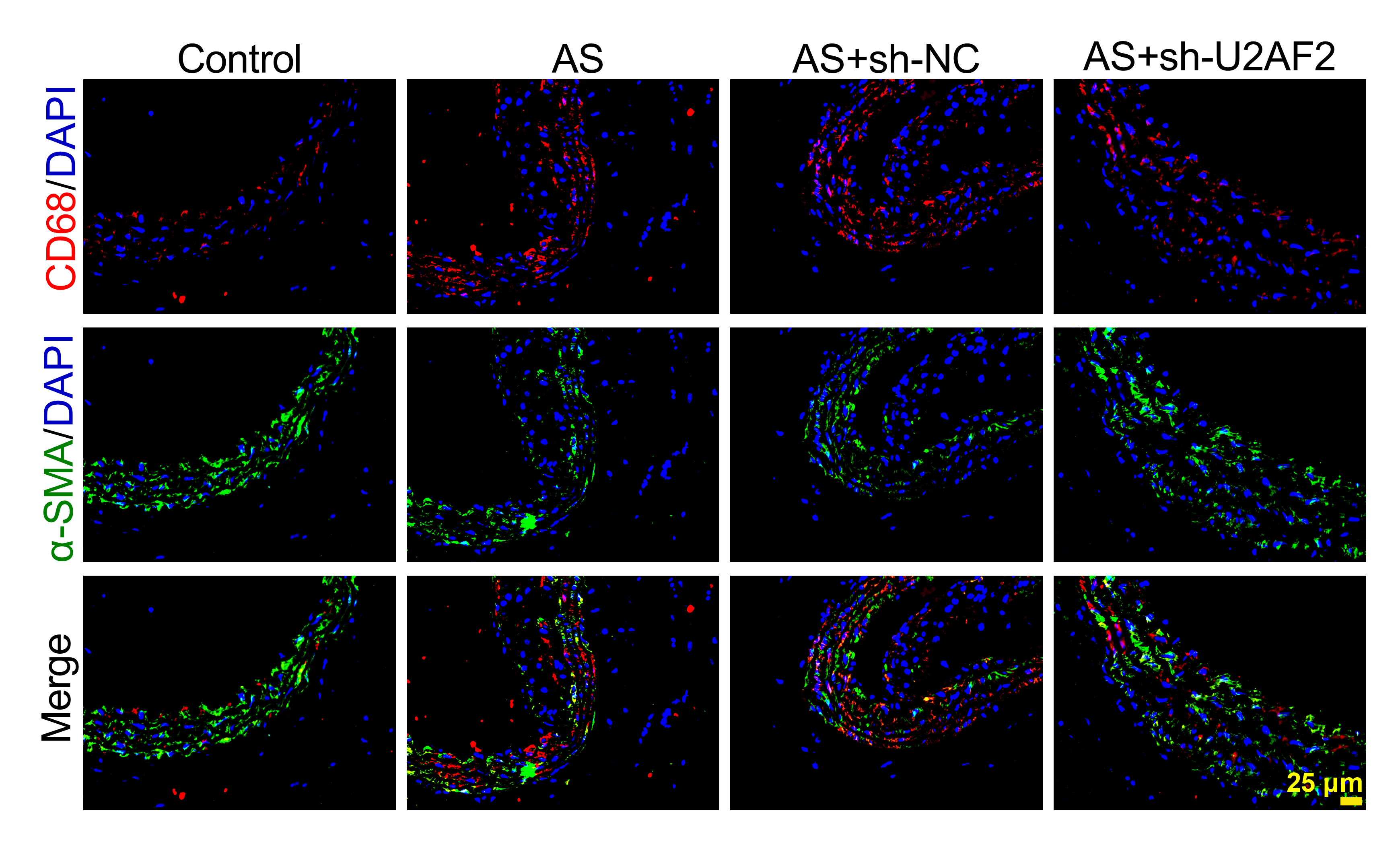

Supplement: Supplementary file 6 — Supplementary Material 6: Fig. S5. CD68 and α-SMA Co-localization detection. Immunofluorescence double staining was used to detect the colocalization of CD68 and α-SMA in aortic tissues from the Control, AS, AS + LV-sh-NC, and AS + LV-sh-U2AF2 groups. NC means negative control of sh-U2AF2 [file 40659_2026_672_MOESM6_ESM.tif]

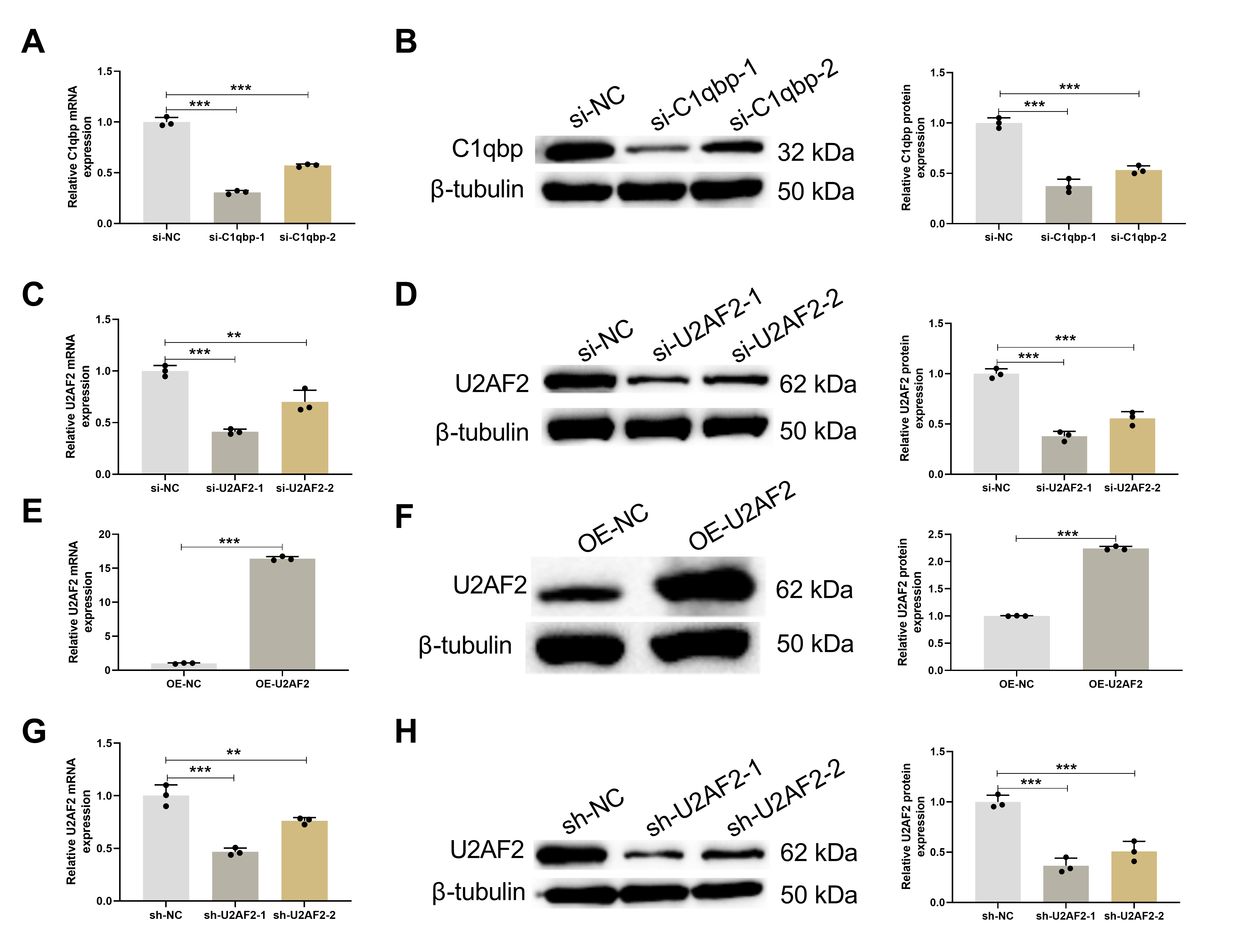

Supplement: Supplementary file 7 — Supplementary Material 7: Fig. S6. Efficiency verification. A, B qRT-PCR and Western blot analysis of C1qbp expression in the si-NC, si-C1qbp-1, and si-C1qbp-2 groups. C, D qRT-PCR and Western blot analysis of U2AF2 expression in the si-NC, si-U2AF2 -1, si-U2AF2 -2 groups. (E-F) qRT-PCR and Western blot analysis of U2AF2 expression in the OE-NC and OE-U2AF2 groups. G, H qRT-PCR and WB analysis of U2AF2 expression in the sh-NC, sh-U2AF2-1, sh-U2AF2 -2 groups. *p < 0.05, **p < 0.01, ***p < 0.001 versus sh-NC/si-NC/OE-NC. NC means negative control of si-C1qbp/si-U2AF2/OE-U2AF2/sh-U2AF2 [file 40659_2026_672_MOESM7_ESM.tif]
